# Analytical Similarity Assessment of Biosimilars: Global Regulatory Landscape, Recent Studies and Major Advancements in Orthogonal Platforms

**DOI:** 10.3389/fbioe.2022.832059

**Published:** 2022-02-09

**Authors:** Neh Nupur, Srishti Joshi, Davy Gulliarme, Anurag S. Rathore

**Affiliations:** ^1^ Department of Chemical Engineering, IIT Delhi, Hauz Khas, New Delhi, India; ^2^ Institute of Pharmaceutical Sciences of Western Switzerland (ISPSO), University of Geneva, Geneva, Switzerland; ^3^ School of Pharmaceutical Sciences, University of Geneva, Geneva, Switzerland

**Keywords:** analytical similarity, biosimilars, critical quality attributes, orthogonal analytical tools, regulatory guidelines

## Abstract

Biopharmaceuticals are one of the fastest-growing sectors in the biotechnology industry. Within the umbrella of biopharmaceuticals, the biosimilar segment is expanding with currently over 200 approved biosimilars, globally. The key step towards achieving a successful biosimilar approval is to establish analytical and clinical biosimilarity with the innovator. The objective of an analytical biosimilarity study is to demonstrate a highly similar profile with respect to variations in critical quality attributes (CQAs) of the biosimilar product, and these variations must lie within the range set by the innovator. This comprises a detailed comparative structural and functional characterization using appropriate, validated analytical methods to fingerprint the molecule and helps reduce the economic burden towards regulatory requirement of extensive preclinical/clinical similarity data, thus making biotechnological drugs more affordable. In the last decade, biosimilar manufacturing and associated regulations have become more established, leading to numerous approvals. Biosimilarity assessment exercises conducted towards approval are also published more frequently in the public domain. Consequently, some technical advancements in analytical sciences have also percolated to applications in analytical biosimilarity assessment. Keeping this in mind, this review aims at providing a holistic view of progresses in biosimilar analysis and approval. In this review, we have summarized the major developments in the global regulatory landscape with respect to biosimilar approvals and also catalogued biosimilarity assessment studies for recombinant DNA products available in the public domain. We have also covered recent advancements in analytical methods, orthogonal techniques, and platforms for biosimilar characterization, since 2015. The review specifically aims to serve as a comprehensive catalog for published biosimilarity assessment studies with details on analytical platform used and critical quality attributes (CQAs) covered for multiple biotherapeutic products. Through this compilation, the emergent evolution of techniques with respect to each CQA has also been charted and discussed. Lastly, the information resource of published biosimilarity assessment studies, created during literature search is anticipated to serve as a helpful reference for biopharmaceutical scientists and biosimilar developers.

## 1 Introduction

Biologics or biotherapeutics are rDNA products used to diagnose, prevent, treat, and cure medical conditions and include a diverse category of products (i.e., proteins, enzymes, peptides, vaccines to name a few). Biotherapeutics are structurally complex compared to small molecules (USFDA, 2018). Currently, biopharmaceuticals are one of the fastest-growing sectors in the biotechnology industry. In the last decade, the landscape for biologics has evolved at an accelerated rate globally with market size of USD 254.9 billion as of 2017 and expected to reach USD 580.5 billion by 2026 at a Compound Annual Growth Rate (CAGR^1^) of 9.5% (2018–2026) (Global, 2018).

**FIGURE 1:**
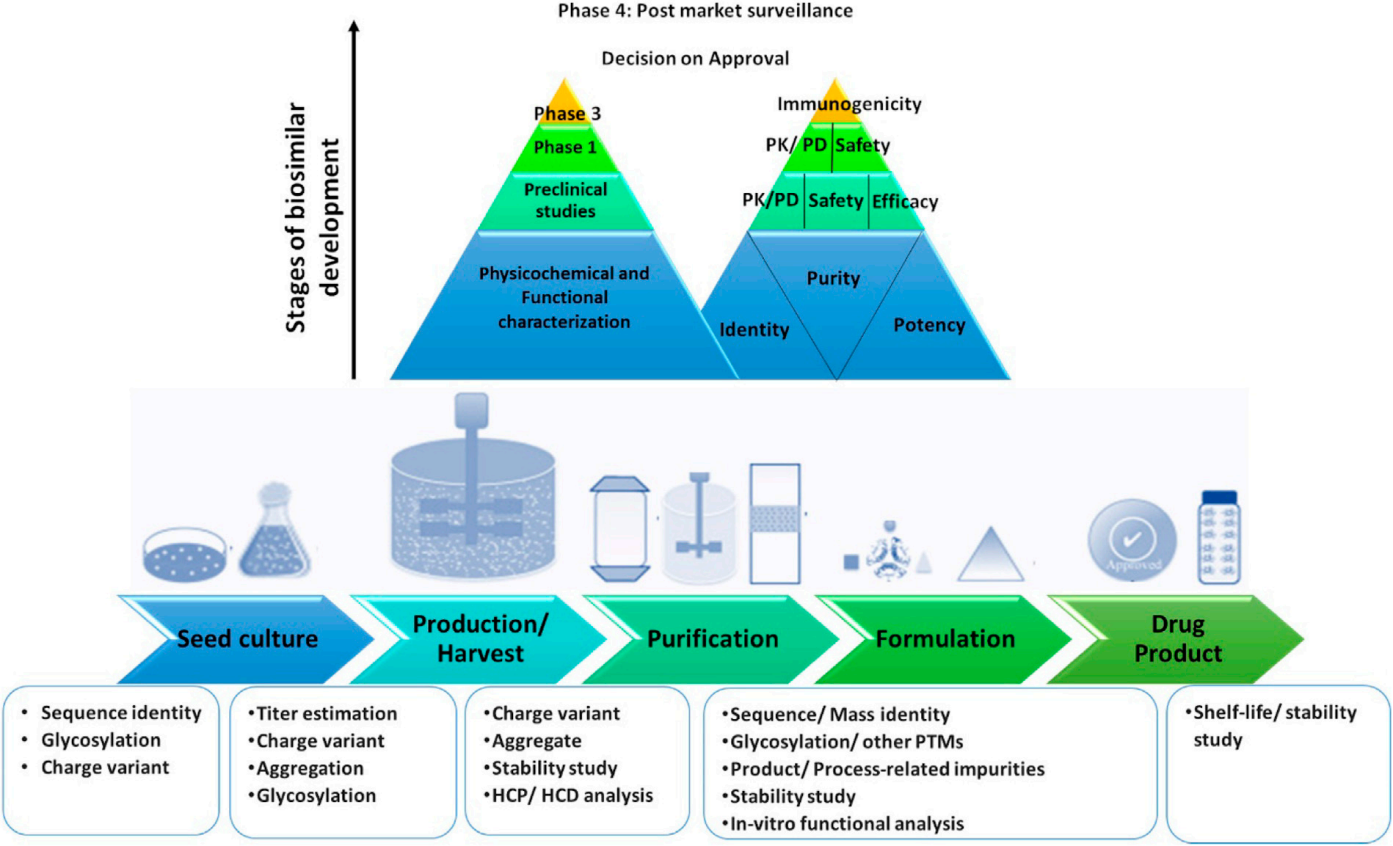
Bioprocess train with multiple stages of development during biosimilar manufacturing. PK, Pharmacokinetics; PD, Pharmacodynamics; PTM, Post translational modifications; HCP, Host Cell Protein; HCD, Host Cell DNA.

As the innovator (aka originator/reference) molecule reaches patent cliff, it paves way for commercialization of biosimilars which are “highly similar” to the innovator in terms of structure and function, notwithstanding minor variations in clinically inactive components and should have no clinically meaningful differences in terms of safety, purity, and potency of the drug product (DP^2^) (USFDA, 2019). This needs to be thoroughly characterized during product development. The product attributes that are critical to the safety, efficacy, and potency of the product are classified as critical quality attributes (CQAs) (Eon-Duval et al., 2012). This exercise that is conducted to establish comparability between the reference product and its intended biosimilar is known as similarity assessment (aka biosimilarity) and comprises detailed comparative physicochemical and functional characterization using appropriate, validated analytical methods (Nupur et al., 2016, 2018; Joshi and Rathore, 2020). As structural attributes are molecule-dependent, CQAs may vary to a certain degree amongst the different modalities. For example, glycosylation is typically a CQA for proteins produced in the eukaryotic systems, such as monoclonal antibodies (mAbs) produced in the mammalian cells. For therapeutics with prokaryotic hosts (such as *Escherichia coli*), glycosylation is not an attribute of concern. Hence, requirements for analytical platforms for characterization and biosimilarity assessments are tailored to be modality-specific and within the larger domain of rDNA products, so are the regulatory requirements. Analytical platforms play a dynamic role in biopharmaceutical and biosimilar manufacturing and in general serves to control/monitor the process. A typical bioprocess train for biosimilar development and associated analysis commonly required at different stages of manufacturing are illustrated in Figure 1.

The requirement of unambiguous biosimilarity demonstration brings us to the concept of orthogonality. Implementation of orthogonal tools (differing in their principle of operation) is invaluable to demonstrate unambiguity in the comparative profiles of CQAs, especially in the cases where the primary technique is qualitative or the CQA is dynamic (i.e., cannot be mapped completely by one technique). The United States Food and Drug Administration (USFDA) non-binding guidance for industry on “Development of Therapeutic Protein Biosimilars: Comparative Analytical Assessment and Other Quality-Related Considerations,” quotes that “methods that use different physicochemical or biological principles to assess the same attribute are especially valuable, because they provide independent data to support the quality of that attribute” and have pointed out the importance of adding orthogonal tools to analytical assessment (USFDA, 2019). A classic example of this is size variants, where orthogonal analytical techniques have been widely employed to 1) cover the breadth of the size range (soluble aggregates < sub visible < visible < insoluble aggregates) and 2) to independently quantify size aggregates in the same size range (orthogonal tools for size variants assessment are discussed in Section 2.2.4.1). Other attributes exemplifying the use of orthogonal tools include higher-order structure (HOS), glycosylation, and charge variants (discussed under Section 4).

Analytical characterization of CQAs for different modalities has been reviewed in some publications (Fekete et al., 2013, 2016; Jacobs et al., 2016, 2017; Santos-Neto et al., 2021). However, the current review focuses on major developments in regulatory approvals and orthogonal analytical platforms for biosimilarity assessment. The global regulatory landscape with respect to biosimilar approvals as well as that in the analytical platforms for similarity studies (till July 2021) has been summarized, with particular focus on progress made in the last 5 years (since 2015). Finally, a discussion on evolution and future trajectory of analytical similarity platforms is also presented. Overall, this review serves as a useful repository of references to help biosimilar manufacturers in designing suitable analytical platforms for similarity studies.

All abbreviations appearing in this publication, including supplementary information have been tabulated as Supplementary Table S1. Definitions and meanings of domain specific terminology have been listed in Supplementary Table S2.

## 2. Global Landscape on Biosimilar Approvals

Following the patent cliff of certain innovator products and the growing support from the major regulatory agencies, there has been a steady increase in the number of biosimilar approvals. At present, region and country-specific biosimilar regulatory pathways and guidance are at different stages of development and implementation (Rathore and Bhargava, 2020; 2021a; 2021b; 2021c). There is a growing interest in increasing global harmonization of the regulatory guidelines for biosimilar development including selection of the reference product, nomenclature, and the design of analytical, non-clinical, or clinical biosimilarity studies. A global agreement on the regulatory requirement for the biosimilars would facilitate standardization of product quality and is likely to positively impact the reception and acceptance of biosimilars worldwide (WHO, 2009, 2013; Kang H.-N. et al., 2020). A brief region and country-wise account of global status on regulatory guidelines for biosimilar approvals are presented in Table 1.

**TABLE 1:** A region/country-wise account of regulatory guidelines for biosimilar approvals to dateJuly, 2021.

Region	Countries	Regulatory agency	Year	Adopted from/Aligned with	Biosimilar approvals (till date)	References
Europe	European Union	European Medical Agency (EMA)	2005	Committee for Medicinal Products for Human Use and International Council for Harmonization (ICH)	69	EMA (1996), EMA (1999),EMA (2005), EMA (2020), CHMP (2005), CHMP (2014)
North America	United States (US)	Food and Drug Administration (FDA)	Initiated in 2010 and finalized in 2015	Biologics Price Competition and Innovation Act, 2010 for Biologic License Application and Section 351(a) and 351(k) of the Public Health Service Act	34	USFDA (2015), USFDA (2019), USFDA (2021)
	Canada	Biologics and Genetic Therapies Directorate under Health Canada	Initiated in 2010 and finalized in 2016	EMA, USFDA, and WHO	26	Canada (2016), Wojtyra (2021)

Asia	Japan	Pharmaceuticals and Medical Devices Agency	2009	EMA	28	PMDA (2020), Rathore and Bhargava (2021a)
	South Korea	Ministry of Food and Drug Safety	Initiated in 2009 and finalized in 2014	EMA and WHO	15	MFDS (2020), Rathore and Bhargava (2021a)
	India	Central Drugs Standard Control Organization and the Review Committee on Genetic Manipulation	Initiated in 2012 and revised in 2016	EMA and USFDA	103	DBT (2016), CDSCO (2020), Rathore and Bhargava (2021a)
	China	Center for Drug Evaluation under National Medical Products Administration	Initiated in 2014 and finalized in 2015	EMA and USFDA	14	GaBI (2021), Rathore and Bhargava (2021a)
	Malaysia	National Pharmaceutical Regulatory Agency	Initiated in 2008 and reframed in 2009	EMA and WHO	25	NPRA (2021), Rathore and Bhargava (2021a)
	Indonesia	National Agency for Drug and Food Control	2015	ICH and EMA	20	Kang et al. (2021), Rathore and Bhargava (2021a)
	Singapore	Health Products Regulation Group	2009	EMA	7	
	Thailand	Food and Drug Administration	2013	WHO	13	
	Iran	Food and Drug Organization under Ministry of Health and Medical Education	2014	WHO	26	
	Jordan	Food and Drug Administration	2015	EMA	9	
	Russia	Министерство здравоохранения Российской Федерации; Rosminzdrav, Minzdrav			34	GaBI (2020), Kang et al. (2021)
Australia	Australia	Therapeutic Goods Administration	Initiated in 2008, finalized in 2013 and revised in 2018	EMA	26	TGA (2018), Health (2021)
Latin America	Argentina	Administracion Nacional de Medicamentos, Alimentos y Tecnologıa Medica	2008	EMA	29	GaBI (2019), Ortiz-Prado et al. (2020), Rathore and Bhargava (2021b)
	Brazil	Agencia Nacional de Vigilancia Sanitaria	2010	WHO	21	
	Mexico	Federal Commission for the Protection against Sanitary Risks	Initiated in 2011 and reframed in 2013		6	
	Peru	Ministerio de Salud	2016	WHO, FDA, and EMA	5	
	Colombia	Ministerio de Salud y Proteccion Social	2013		3	
	Chile	Agencia Nacional de Medicamentos	Initiated in 2011 and finalized in 2014		15	
	Venezuela	Instituto Nacional de Higiene “Rafael Rangel”	2012		1	
	Cuba	Center for State Control on the Quality of Drugs	2011	WHO	16	
Africa	Egypt	Central Administration for Pharmaceutical Affairs with National Organization for Research and Control of Biologics under Egyptian Drug Authority	2013		4	Pategou (2020), Kang et al. (2021)
	Ghana	Food and Drugs Authority	Initiated in 2013 and reframed in 2019		13	
	South Africa	South African Health Product Regulatory Authority	Initiated in 2010 and reframed in 2014	EMA	2	

The European Medicines Agency (EMA) pioneered the legal framework and regulatory approval pathway for biosimilars in 2005, paving the way for other jurisdictions around the globe (Gherghescu and Delgado-Charro, 2020). In alignment with the International Council for Harmonization (ICH) guidelines, the EMA’s “Guideline on Similar Biological Medicinal Products” states that similarity to the innovator needs to be established in terms of quality characteristics, biological activity, safety, and efficacy based on a comprehensive, head-to-head biosimilarity exercise for characterization of the quality of the DP and the approval is subjected to the totality-of-evidence presented (EMA, 1996, 1999, 2005; CHMP, 2005, 2014). The World Health Organization (WHO) adopted similar guidelines on the evaluation of Similar Biotherapeutic Products (SBPs) in 2009 to ensure better access to safe and effective SBPs worldwide through global harmonization of the regulatory framework for licensure (WHO, 2009). The WHO laid the foundation for other regulatory authorities to introduce their respective guidelines by giving to the national regulatory authorities (NRAs) the flexibility to adapt approval pathways according to their needs. Around the same time, Biologics Price Competition and Innovation Act (BPCI) were developed in the US that initiated the biosimilar approval pathway under USFDA. Since then, several countries have laid down and implemented a regulatory framework for biosimilar approval for its commercial use within their jurisdiction (Table 1). All guidance documents thus far emphasize the demonstration of biosimilarity *via* extensive structural and functional characterization followed by non-clinical, pharmacokinetic, and clinical studies. The degree of biosimilarity with respect to product quality determines the scope and breadth of the required non-clinical and clinical data, on a case-by-case basis, dependent on the product class/modality. The comparative clinical studies are encouraged to be specifically developed to rule out clinically relevant differences in safety or efficacy between the biosimilar and the innovator, in order to confirm biosimilarity (WHO, 2013).

**FIGURE 2:**
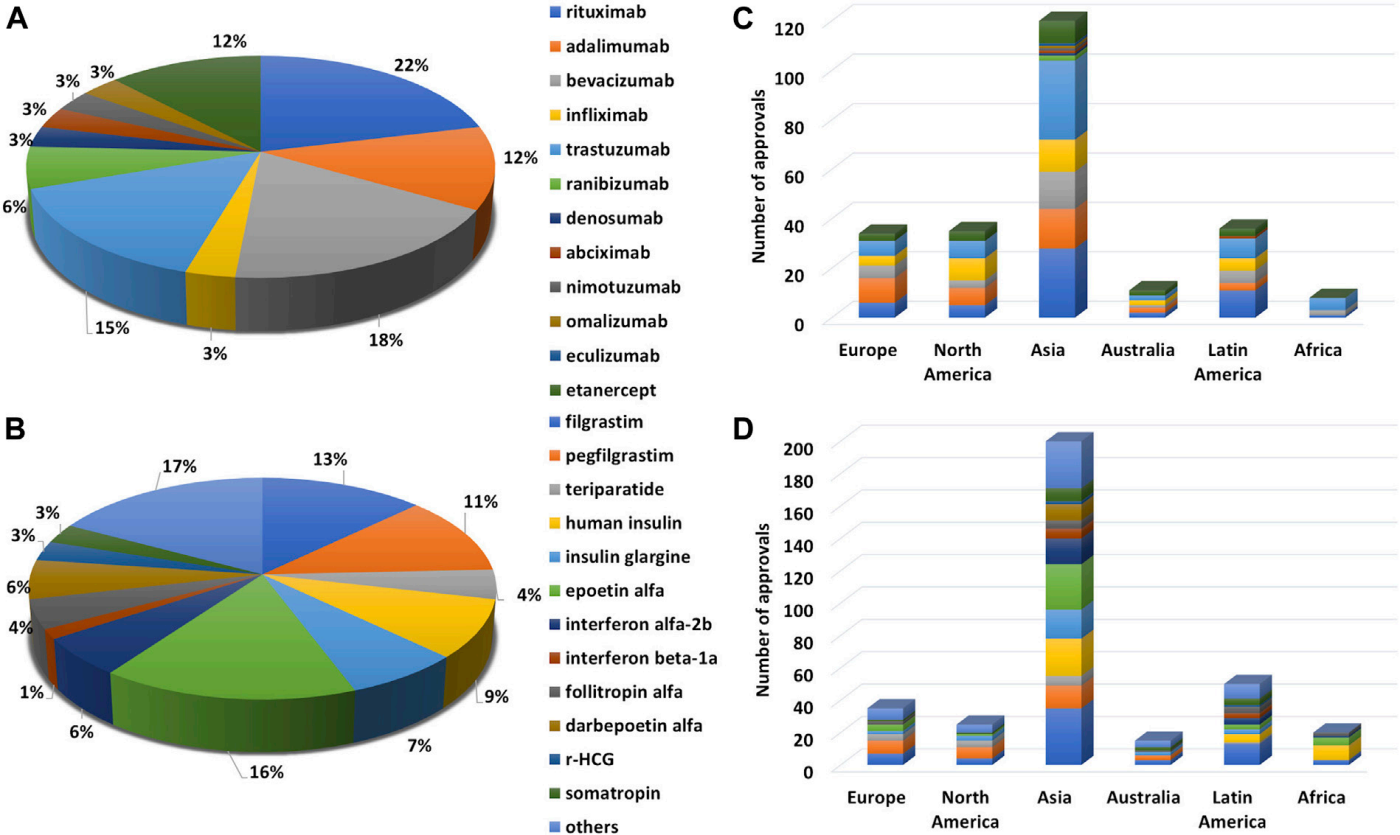
Trend in biosimilar approval for different modalities depicted as percentage of total approvals for a given biologic under **(A)** mAbs and **(B)** non-mAbs and global biosimilar approvals (in numbers) across continents i.e., Europe, North America, Asia, Australia, Latin America, and Africa for **(C)** mAbs and **(D)** non-mAbs.

The global regulatory landscape continues to evolve in response to the mushrooming biosimilar industry, with close to 600 approved biosimilars for 45 reference products in over 50 countries, to date. In addition to Table 1, a comprehensive region/country-wise list of mAb and non-mAb biosimilar approvals has been tabulated in Supplementary Table S3. Due to several factors such as clinical indications, market size, patent cliffs, and the need for affordable alternatives, mAbs constitute a major segment in the overall biosimilars portfolio. This is evidenced by a total of 249 biosimilars that currently populate the market for 12 reference products. Of these, the leading mAbs include anti-HER2 trastuzumab (60), anti-CD20 rituximab (53), anti-TNF α adalimumab (38) and infliximab (33), and anti-VEGF bevacizumab (31), and the anti-TNF Fc-fusion protein, etanercept (26) (Figure 2A). The majority of these approvals have been granted in Asia (50%) followed by Latin America (15%), Europe (14%), North America (14%), Australia (4%), and Africa (3%) (Figure 2B). Taking the rest of the modalities together as non-mAbs, a total of 348 biosimilars for 33 reference products belonging to filgrastim (65, GCSF receptor binding) followed by epoetin alfa (41, JAK-STAT receptor binding), human insulin (37, insulin receptor binding), peg-filgrastim (32, GCSF receptor binding), insulin glargine (28, insulin receptor binding), and interferon alfa-2b (21, IFN-α/β receptor binding) have been approved so far (Figure 2C). The geographic spread of these approvals is similar to that of mAbs with a maximum number of biosimilar approvals granted in Asia (58%) followed by Latin America (15%), Europe (10%), North America (7%), Africa (6%) and Australia (4%) (Figure 2D).

**FIGURE 3:**
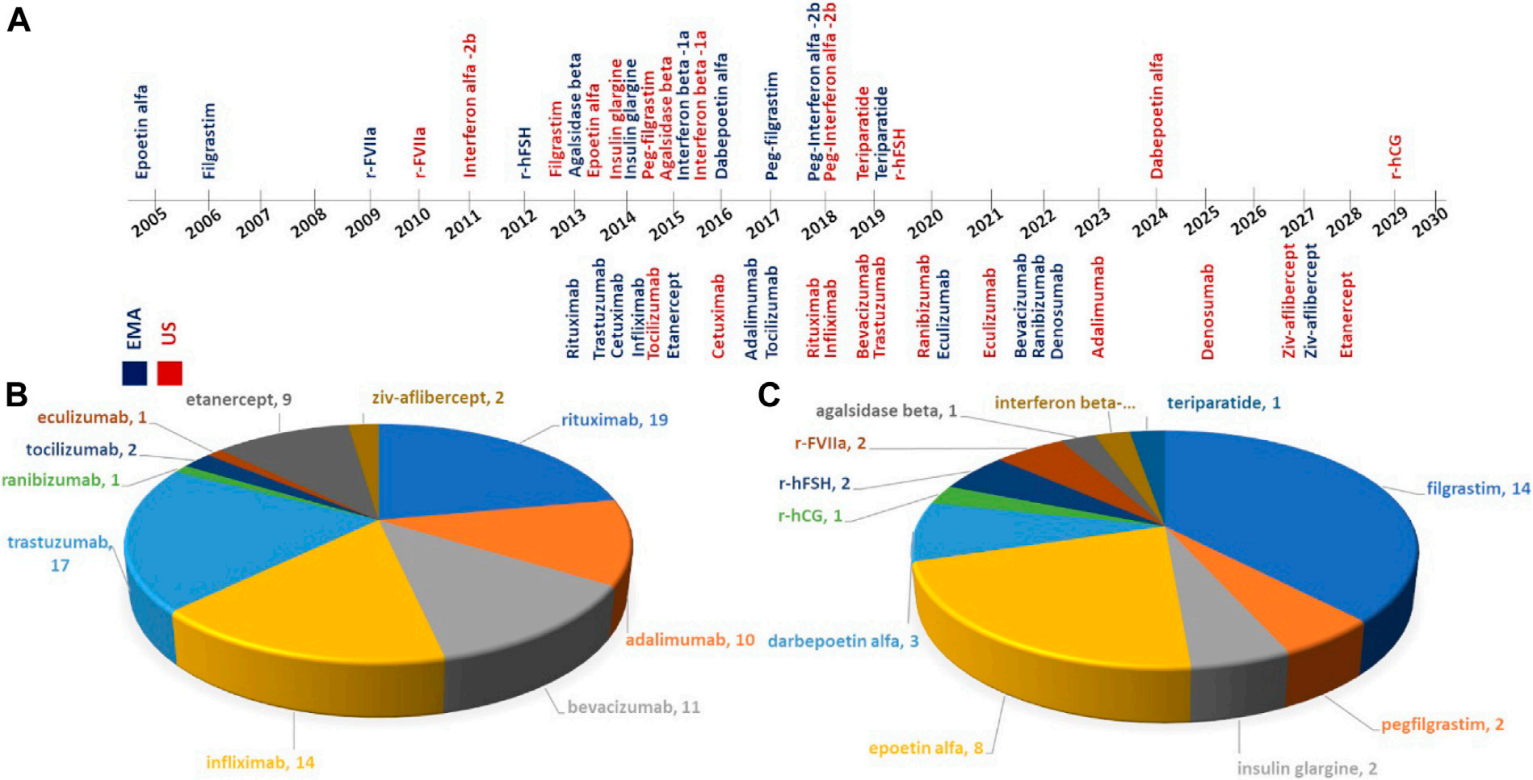
**(A)** Timeline on patent expirations of reference products in European Medicines Agency; EMA (Moorkens et al., 2020) and U.S. Food and Drug Administration; USFDA (Derbyshire and Shina, 2019), publications on analytical similarity studies for biosimilars under **(B)** mAbs, and **(C)** non-mAbs.

Recent patent expirations (2020-21) include ranibizumab (2020-US) and eculizumab (2020-US, 2021-EU). Other major biologics including blockbuster drugs nearing patent cliff in this decade include Bevacizumab (2022-EU), ranibizumab (2022-EU), Denosumab (EU-2020), adalimumab (US-2023), Denosumab (US-2025), Ziv-aflibercept (EU/US-2021 and Etanercept (2028-US) for mAb based biologics and Dabepoetin alpha (2024-US) and r-hCG (2029-US) in non-mAb biologics (Figure 3A).

The affordability of biosimilars largely depends on remissions in clinical studies granted on the basis of analytical and functional biosimilarity assessment data presented to the regulators. Despite a large number of approvals, public availability of information related to biosimilarity assessment is scattered and limited. Here, we have collected information with respect to analytical biosimilarity studies published as peer-reviewed research articles and offer a comprehensive account of the same in the following section.

### 2.1 Analytical Biosimilarity Assessment in the Published Literature

Head-to-head structural and functional biosimilarity assessment is a non-trivial, resource-intensive exercise since there is a need to use multiple methods for analytical and functional characterization. Most regulatory guidelines recommend the use of a gamut of orthogonal, high-resolution, analytical tools for qualitative and quantitative characterization of CQAs. Although several biosimilars have been approved to date, publicly available repositories of published literature on analytical biosimilarity studies remain limited (Ishii-Watabe and Kuwabara, 2019; Alsamil et al., 2020; Ratih et al., 2021; Safdar et al., 2021). In this section, we have focused on peer-reviewed analytical biosimilarity studies published so far, with a focus on analytical platforms used for characterization of each CQA. The methodology adopted for the selection of relevant publications from search engines has been detailed in Supplementary Material. The final database consisted of 116 publications presenting analytical biosimilarity studies for approved/intended biosimilars available in the public domain with details on the CQAs assessed and analytical tools used to date (Supplementary Table S4).

To the best of our knowledge, the first peer-reviewed biosimilarity study was published in 2006 where biophysical comparability of Epoetin alfa was carried out comparing the analytical profiles of Eprex^®^ (prefilled syringes) with the innovator, namely Epogen^®^. It is a recombinant human erythropoietin that stimulates red blood cell production and is approved by FDA for use in treatment of anemia due to chronic kidney disease or cancer treatments. (Deechongkit et al., 2006). The innovator product Epogen^®^ manufactured by Amgen reached patent cliff in 2005 in Europe and 2013 in US following which several biosimilars and intended copies have entered the markets. The market for the molecule continues to grow (USD9,243.12 million in 2020), and is projected to reach USD14,414.59 million by 2028. The annual sales of Epogen^®^, the innovator product was reported at 598 million USD for financial year 2020 (Amgen, 2021; Nerkar et al., 2021). Since epoetin alpha, 10 mAbs and 15 non mAbs have reached the patent cliff in either the US or EMA and the peer-reviewed biosimilarity studies have been published for most off-patent products (Figure 3A, Supplementary Table S5). About 80 analytical biosimilarity studies covering 8 off-patent mAbs (i.e., rituximab, trastuzumab, bevacizumab, infliximab, adalimumab, ranibizumab, tocilizumab, and eculizumab) and 2 Fc-fusion proteins (i.e., etanercept and ziv-aflibercept) have been published so far (till July 2021). Interestingly, there are few instances where analytical studies have been published for the parent molecule (ziv-aflibercept) yet to reach patent expiration (due date 2027) (Hermosilla et al., 2020; Shen et al., 2021). Similarly, in the case of non-mAbs, 36 analytical biosimilarity studies covering 11 non-mAbs (i.e., filgrastim, peg-filgrastim, epoetin-α, darbepoetin-α, interferon-β, recombinant activated factor VII (rFVIIa), insulin glargine, recombinant human follicle stimulating hormone (r-FSH), agalsidase-β, recombinant human chorionic gonadotropin (r-hCG), and teriparatide) are accessible in the public domain (till July 2021). The most represented molecules in these studies include rituximab (19), trastuzumab (17), filgrastim (14), infliximab (14), bevacizumab (11), adalimumab (10), and etanercept (9) (Figures 3B, C).

Broadly, the most relevant CQAs of biotherapeutic products can be categorized under primary structure, higher-order structures (HOS), glycosylation (eukaryotic hosts), product-related variants, and process-related variants. The primary structure is further divided into intact/subunit mass analysis, amino acid sequence/peptide mapping, and disulfide bridge/free sulfhydryl group; HOS into the secondary structure, tertiary structure, and conformational stability; glycosylation into oligosaccharide pattern, glycopeptide mapping, and monosaccharide/sialic acid content; product-related variants into size variants, charge variants and related proteins arising out of post-translational modifications, i.e., aggregates, fragments, C-terminal lysine loss, N-terminal pyroglutamate cyclization, methionine oxidation, asparagine deamidation, aspartate isomerization, glycation, phosphorylation, acetylation, acylation, misfolding and process-related variants into host cell proteins (HCPs) and host cell DNA (HCD). A minimum requirement of at least one technique under each of the above-mentioned categories is mandatory for assessment, except in cases where the nature of the therapeutic of interest allows for the exclusion of analyzing certain attributes. For example, biosimilars expressed in *E. coli* do not require glycosylation analysis as the expression system is not capable of performing glycosylation.

For interested readers, a comprehensive account of CQA-wise and tool-wise analytical biosimilarity studies in published literature covering orthogonal analytical tools has been tabulated in Supplementary Table S6. From published studies, most assessed CQAs were primary structure (peptide mapping/amino acid sequence covered in 76 studies), followed by product-related variants (size variants covered in 75 studies), HOS (tertiary structure covered in 63 studies), and glycosylation (58 studies). This is not surprising as biosimilarity exhibited across the structural hierarchy is indicative of preservation of the functionality and hence efficacy of the intended biosimilar.

**FIGURE 4:**
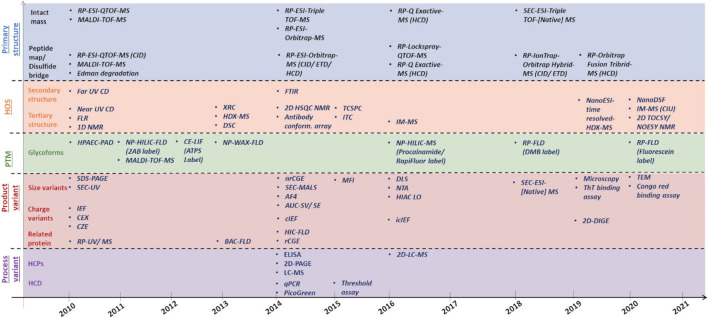
Evolutionary timeline of analytical platforms used for different CQAs i.e., primary structure, HOS, glycosylation, product-related and process-related variant with respect to analytical biosimilarity assessment.

While assessing biosimilarity, one is really looking at the structural fingerprint of the biosimilar DP/DS and comparing it to the innovator. The strength of the evidence gathered is hence directly dependent on the resolution and sensitivity of the techniques employed. Moreover, in certain instances, one technique *per* attribute may not be sufficient as the attribute in question may be multi-faceted and hence requires multiple orthogonal tools. HOS and size variants are two such examples. Hence orthogonal tools serve as a means to 1) corroborate evidence for biosimilarity and 2) map multiple facets of a complex attribute independently, with an overall aim of reducing ambiguity. Having said that, there is significant commonality in CQAs for therapeutic molecules as elements of structural identity and integrity such as primary and HOS which are applicable to all proteins. Therefore, primarily the biosimilarity platforms consist of established biophysical techniques used in protein characterization. Over time and follow-on technical advancements, orthogonal assessment means have corroborated the skeleton of analytical characterization platforms (Figure 4). Major technical developments since 2015 have been outlined and discussed below.

### 2.2 Recent Advancements and Orthogonality in Analytical Similarity Assessment

As outlined in the USFDA guidelines on the comparative analytical assessment of biosimilars, detailed analysis of CQAs should be conducted using orthogonal analytical platforms including both established biophysical tools and new technologies, since each technique has its own merits and demerits (USFDA, 2019). The development of methods and techniques for orthogonal assessment of CQAs is hence a burgeoning field and over the years, some of these methods and techniques have been adopted for routine biosimilarity studies (Supplementary Tables S6, S7 and Figure 4). In comparison to other CQAs, HOS and/or stability are the most represented with respect to publications reporting application of a new technology/technique for comparative analysis of biotherapeutics (covered in 28 studies) followed by glycan profiling (covered in 21 studies), charge variants (covered in 15 studies), peptide mapping (covered in 11 studies), size variants (covered in seven studies), and other attributes (other PTMs, glycation, disulfide bond, free sulfhydryl content, HCPs, and effector binding). Data acquisition aside, there has also been an increase in publications related to the application of advanced statistical methods for the evaluation of chromatography/spectroscopy data for comparative analysis (nine studies).

#### 2.2.1 Primary Structure

**FIGURE 5:**
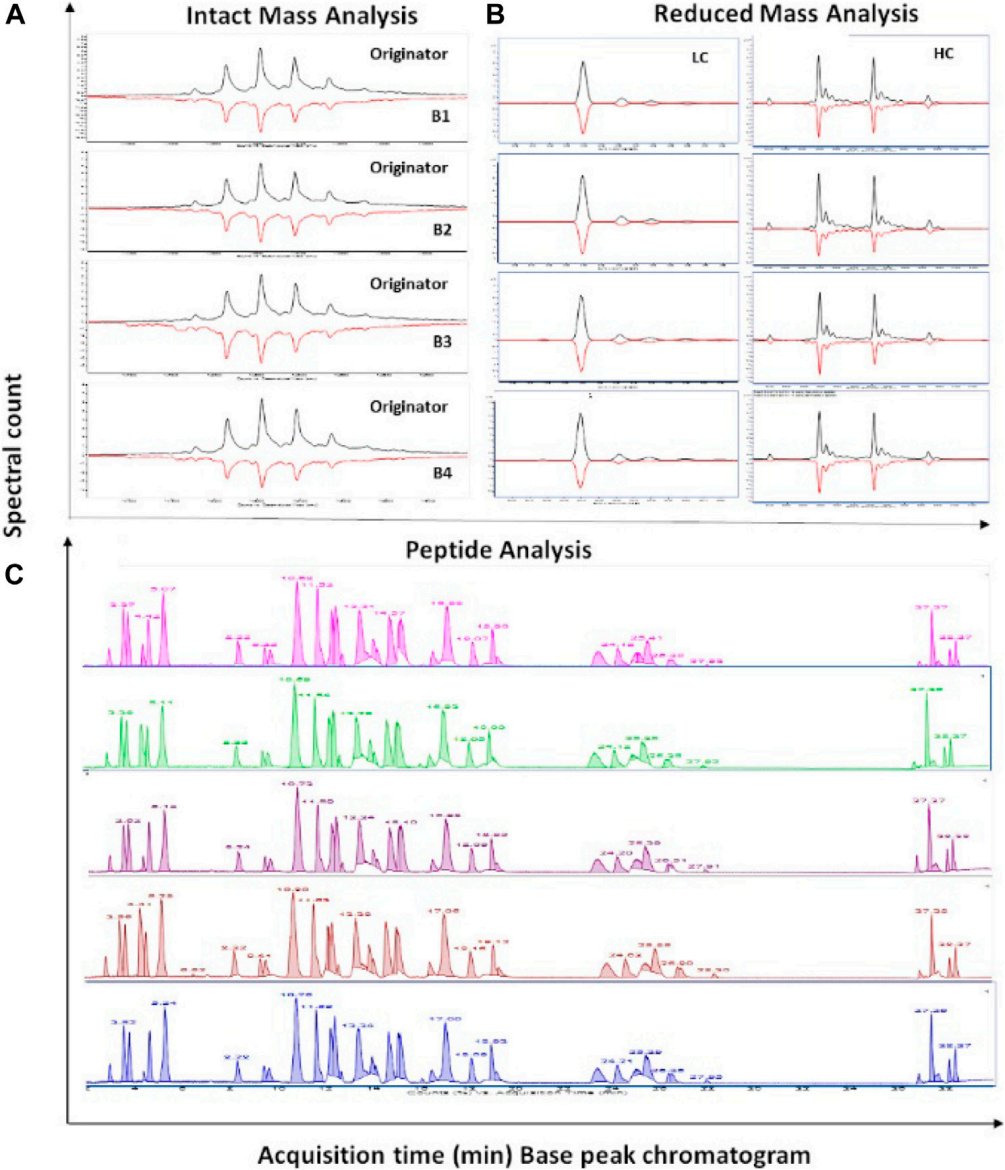
RP-LC-MS based primary structure biosimilarity assessment of trastuzumab originator and biosimilars as adapted from Joshi and Rathore, 2020. **(A)** mirror plot of Intact profile, **(B)** mirror plot of reduced profile and **(C)** stacked chromatograms of trastuzumab biosimilars compared with originator.

Confirmation of primary structure involves mapping of the amino-acid sequence (sequence identity) and measuring the exact mass (mass-identity) of the biosimilar compared to the innovator (Figure 5). Sequence identity can be established by both Edman degradation and mass spectrometry (MS). Edman degradation, a traditional tool that involves successive removal of N-terminal amino acids by chemical methods, has featured in several publications across the years (Lee et al., 2013; Crobu et al., 2014). However, MS has increasingly become the more popular technique for both sequence and mass identity due to its sensitivity, versatility, fast turnaround and enhanced resolution. Moreover, additional information related to glycosylation and other PTMs can also be obtained through MS within a single analysis (Singleton, 2014). Over the years, MS platforms that have been featured in analytical similarity assessment publications for primary structure analysis include MALDI-TOF-MS, RP-ESI-QTOF-MS, RP-ESI-IonTrap/Orbitrap/Q-Exactive-MS, LC-ESI-QTOF-[Native] MS (Figure 4, Supplementary Table S4).

For intact mass analysis, there have been recent advancements in developing LC-ESI-[Native] MS-based methods that offer an orthogonal alternative to traditional denaturing RP-ESI-MS where other LC modalities (such as SEC, HIC or IEX) are explored to determine intact mass with possible sequence variants under non-denaturing (native) conditions to identify biologically active species and monitor protein dynamics. SEC-ESI-QTOF-MS has been employed in analytical biosimilarity assessment of biosimilar candidates of Amgen’s bevacizumab (ABP 215) (Seo et al., 2018) and infliximab (ABP 710) (Saleem et al., 2020), Sun Pharma’s rituximab (SB-02) (Singh et al., 2018) and Celtrion’s infliximab (CT-P13) (Hermosilla et al., 2019) compared to the respective innovators. The advantage of SEC-MS over RP-MS is that the native structure is preserved, and so the information gained is not only a comparison on exact mass, but also of the higher-order structure of the intended biosimilar. In acknowledgment of this, a host of native MS-based analyses such as native-MS, ion mobility (IM)-MS, and time-resolved hydrogen-deuterium exchange (HDX)-MS have been employed to provide an unambiguous assessment of the structural, dynamic, and chemical biosimilarity for Apobiologix’s bevacizumab biosimilar (Brown et al., 2019) (Supplementary Table S4).

For peptide mapping, the constant evolution of peptide ion fragmentation types, such as electron-transfer dissociation (ETD) and/or Higher-energy C-trap dissociation (HCD) versus collision-induced dissociation (CID) along with the use of LC-QTOF, LC-Ion-Trap-Orbitrap and LC-Q-Exactive-Orbitrap MS has resulted in improvements in accurate identification and quantification of sequence variants and mutations. In two different studies, complete sequence coverage (100%) was obtained after trypsin/Lys-C digestion by targeted and non-targeted comparison of the tryptic map for comparing trastuzumab biosimilars (Chen et al., 2013). Also, stable isotope labeling by amino acids in cell culture (SILAC) by dimethyl labeling [i.e., 2CH2 (rituximab) and 2CD2 (RNAi-mediated rituximab)] were used to detect sequence variants along with disulfide linkages, PTMs, and mutations (Li et al., 2013). LC-QTOF has been used for the complete amino acid sequence analysis of Celtrion’s infliximab (Remsima^®^) (Jung et al., 2014), trastuzumab (CT-P6) (Lee J. et al., 2018), rituximab (CT-P10) (Lee K. H. et al., 2018), Sun Pharma’s rituximab (SB-02) (Singh et al., 2018) and Intas’s peg-filgrastim (INTP5) (Shekhawat et al., 2019) biosimilars. Similarly, in recent years LC-Ion-Trap/Orbitrap Hybrid MS has been employed for characterization of certain biosimilars such as Amgen’s bevacizumab (ABP 215) (Seo et al., 2018), rituximab (ABP 798) (Seo et al., 2020), infliximab (ABP 710) (Saleem et al., 2020), and Kyowa’s adalimumab (Hulio^®^: FKB327) (Schreiber et al., 2020) biosimilars, and LC-Q-Exactive-Orbitrap-MS was used for China’s bevacizumab (BVZ-BC) (company name not indicated in associated publication) (Yu et al., 2020) and Amgen’s eculizumab (ABP 959) (Hutterer et al., 2021) biosimilars, to determine similar amino acid sequence, PTM profiles, and disulfide linkages compared to the respective innovators.

RP-based peptide mapping has certain limitations including poor retention of small/hydrophilic peptides, limited peak capacity, and reduced sample throughput due to the need for re-equilibration between separations. This has largely been addressed through multi-dimensional LC platforms such as 2D-LC. Application of comprehensive two-dimensional liquid chromatography (2D-LC or LC × LC) as a tool for peptide mapping for trastuzumab has been demonstrated (Vanhoenacker et al., 2015). The method addresses identity, purity, and comparability of trastuzumab *via* three different combinations of LC × LC namely SCX, RP, and HILIC in the first dimension coupled with RP in the second dimension, respectively. In other studies, application of 2D-LC-MS has been demonstrated for antibody digests, with online-column digestion, followed by Orbitrap-MS for targeted peptide monitoring and chemometrics (Pérez-Robles et al., 2017; Shatat et al., 2018) (Supplementary Table S7).

Due to its sensitivity and dynamic range, more work is being done towards maximizing the capability of LC-MS-based formats through a more holistic characterization/comparability strategy including multiple levels of analysis (intact, subunit as well as peptides level). These multi-attribute methods (MAM) use a combination of high mass accuracy/high-resolution MS along with automated identification and relative quantification of the attributes (peptide mapping, glycopeptide, deamidation, and oxidation) with dedicated software. A head-to-head biosimilarity of trastuzumab and cetuximab with biosimilar candidates has been demonstrated at all levels including peptides and glycopeptides produced by enzymatic digestions (e.g., trypsin, Lys-C, Asp-N, pepsin) and analyzed on nanoLC-QTOF-MS (Beck et al., 2015). MAM formats have also been demonstrated on formats such as LC-Orbitrap-MS. With respect to biosimilarity assessment, the new peak detection feature of the method has particular applicability as it automatizes the identification of new peaks in tested samples (Rogers et al., 2015, 2018) (Supplementary Table S7). The method has since been adopted in QC to release testing and MAM consortium has been formed to harmonize best practices and generate methodologies in the facilitation of the widespread integration of the MAM platform in QC labs (Millán-Martín et al., 2020). Recently, industry-wide inter-laboratory study (28 participating labs) using pre-digested samples of the NISTmAb RM 8671 and pre-defined experimental protocol has been conducted to test the robustness of MAM methods (Mouchahoir et al., 2021) (Supplementary Table S7). A version of the methods suitable for process development has been successfully implemented in three Sanofi sites with automatization of sample preparation as well as data interpretation (Song et al., 2021).

Other orthogonal platforms for primary structure assessment include MALDI-TOF-MS which offers a unique alternative to ESI-TOF-MS by producing less complicated spectra due to multiple charging and provides a significant tolerance against salts for fast and easy sample preparation. Few studies have reported the use of MALDI-TOF-MS for peptide mapping and disulfide bridging analysis of non-innovator versions, e.g., etanercept (AVG01) (Maity et al., 2011), TNFR-hyFc fusion protein (Lee et al., 2013), filgrastim (BK0023) (Crobu et al., 2014), and filgrastim (EP2006) (Sörgel et al., 2015) (Supplementary Table S4). In one of the study, chemometrics-based “nearness similarity index” was applied, as a mathematical comparison method applicable to complex mass spectrometric data, particularly in peptide maps obtained by MALDI-TOF-MS (Pérez-Robles et al., 2017) (Supplementary Table S7). The method was applied to identify changes in long-term stability assessment of infliximab and rituximab and should be applicable in biocomparability studies.

#### 2.2.2 Higher-Order Structure

Higher-order structure (HOS) is an umbrella term for three-dimensional (3D) conformations and includes multiple levels of structural hierarchies (i.e., secondary, tertiary and, quaternary). The concept of orthogonality is perhaps most celebrated for HOS characterization, since no one tool can map HOS in its entirety, hence multiple orthogonal tools are used for each level. Here, we have highlighted the evolution of orthogonality in published biosimilarity assessment studies (Figure 4) and discussed methods and techniques developed that have shown application towards biosimilarity assessment (Supplementary Table S7).

##### 2.2.2.1 Secondary Structure

Initial biosimilarity assessment studies published on comparability of Eprex^®^ (epoetin-α), Nivestim™ and Zarzio^®^ (filgrastim) to their respective reference products showed secondary structure comparability through overlay of far ultraviolet circular dichroism (far UV CD) spectra (Deechongkit et al., 2006; Skrlin et al., 2010; Srgel et al., 2010). Later, Fourier transform infrared (FTIR) spectroscopy has also been incorporated either instead of or more commonly as an orthogonal assessment technique (Jung et al., 2014; Liu et al., 2016). Portfolio of biosimilars where CD and/or FTIR has been used for evaluation of secondary structure biosimilarity to reference product is quite diverse and includes both mAb and non-mAb products (Supplementary Table S4).

Lately, deconvolution of CD spectra (Fazel et al., 2019; Hermosilla et al., 2020) and IR absorption spectra (1700–1,600 cm^−1^) (Lee et al., 2019; Shekhawat et al., 2019) using different software (K2D2, BeStSel, OriginPro, etc.) have been successfully used to quantitate secondary conformations (i.e., alpha-helix, beta-sheet, or random coil).

##### 2.2.2.2 Tertiary Structure

Tertiary structures are 3D conformations. Near UV CD (250–300 nm) and fluorescence (FLR) spectroscopy have been traditionally the methods of choice for tertiary structure determination. In biosimilarity studies, these two techniques have been used either individually or together (Moro Pérez et al., 2019; Zhang et al., 2020). However, there have been limited studies involving the use of X-ray crystallography (XRC), a traditional technique for protein structural studies at the near-atomic level, as it works well only with proteins that can be crystallized, it requires expensive instrumentation, trained operators, and extensive time for analysis (Lerch et al., 2020). If we look at the evolution of HOS assessment in biosimilarity studies (Figure 4), the timeline is peppered with multiple orthogonal techniques such as single dimension NMR (Visser et al., 2013; Sörgel et al., 2015; Montacir et al., 2017), multi-dimension NMR (Shekhawat et al., 2019; Bor Tekdemir et al., 2020; Kovács et al., 2020), HDX-MS (Cho et al., 2016; Brokx et al., 2017; Brown et al., 2019), IM-MS (Fang et al., 2016; Montacir et al., 2017, 2018), antibody conformational array (Jung et al., 2014; Hong et al., 2017), aptamer-based enzyme linked apta-sorbent assay (Wildner et al., 2019), and small angle X-ray scattering (SAXS) (Narvekar et al., 2020) (Supplementary Table S4). Of these, NMR has emerged as the new gold standard for HOS assessment with more than 10 published biosimilarity studies published with either 1D, or 1D and 2D as a part of the analytical platform (Supplementary Table S4).

The popularity of NMR is also evident by the fact that by 2016, 15% of BLAs to USFDA included HDX-MS and NMR data for HOS characterization (USFDA, 2016). NMR is one of the high-resolution biophysical techniques for obtaining information regarding protein structure, dynamics, and flexibility at the atomic level by mapping the individual atoms linkage with each other in the 3D space. However, its application for large molecules such as mAbs has been limited until recently. ^1^H NMR has been implemented for HOS assessment, where pulsed-field gradient stimulated echo (PGSTE) experiment has been used to generate highly resolved spectra of intact mAbs in formulation buffers (Poppe et al., 2015) (Supplementary Table S7). Based on differences in the translational diffusion coefficients of molecules in the NMR sample, virtually all other undesired signals arising from water and excipients could be removed. The PROFILE method has since been implemented for similarity assessment studies with the application of chemometrics for statistical analysis of similarity.

Following 1D NMR, the applicability of 2D formats has also shown great promise in biosimilarity assessment, where two different isotopes have been used to generate each spectrum (^13^C, ^1^H, ^15^N usually) and provide a series of ^1^H or ^13^C spectra, represented as a 2D diagram reflective of the HOS. Sophisticated sample preparation (radioactive labeling) required for NMR, along with the long acquisition time has been addressed in these studies along with characterization of different heterogeneities (charge variants, size, PTMs, glycosylation) present within the formulation. Seminal work in this field with respect to method validation and standardization for characterization of mAb-based modalities has been carried out by, or in association with NIST and made available as a series of publications (Arbogast et al., 2015, 2016, 2017; Brinson and Marino, 2019; Brinson et al., 2020; Sheen et al., 2020) (Supplementary Table S7). Recent studies have shown structural elucidation of mAbs at natural abundance *via* 2D-^13^C NMR in as little as 30 min, along with evidence for high correlation of spectra from individual Fab and Fc fragments with intact mAb (Arbogast et al., 2015), as well as application of chemometrics for NMR spectral analysis and biosimilarity studies (both 1D and 2D-NMR) (Arbogast et al., 2016, 2017; Japelj et al., 2016; Chen et al., 2018; Brinson et al., 2020; Sheen et al., 2020) (Supplementary Table S7). In addition to HOS, NMR has also been shown as an orthogonal tool for the assessment of size variants in biotherapeutics (Patil et al., 2017; Joshi et al., 2021) (Supplementary Table S7). In the last couple of years, more work has been done on the identification of PTMs through NMR, although suitability for comparative analysis is yet to be demonstrated (Hinterholzer et al., 2019, 2020) (Supplementary Table S7).

Another orthogonal technique for dynamic HOS comparability is HDX-MS. It elucidates protein conformational dynamics, protein folding, and protein-ligand interactions. HDX-MS relies on the coupling of low-temperature UHPLC with the sensitivity and resolution of MS to determine the locations and rates of amide hydrogen deuterium uptake. In the last 5 years, there has been methodological advances w.r.t range of temperature required for HDX experiments, method reproducibility, significance testing, and application of HDX-MS for similarity assessments. This includes the optimization of HDX-MS methodology based on manual solid-phase extraction to allow a fast and simplified conformational analysis of proteins under pharmaceutically relevant formulation conditions as demonstrated on interferon-β-1a in-formulation samples (E. Nazari et al., 2016) (Supplementary Table S7).

Similar to the development of NMR methods for biopharmaceutical analysis, an interlaboratory method validation study to evaluate the reproducibility of HDX-MS has also been conducted (Hudgens et al., 2019) (Supplementary Table S7). The study determined the reproducibility of continuous-labeling, bottom-up HDX-MS measurements from the Fab fragment of NISTmAb reference material (PDB: 5K8A) in an inter-laboratory comparison study comprising 15 laboratories (Cummins et al., 2016) (Supplementary Table S7).

Harmonization of HDX-MS methodology for biopharmaceutical analysis has also led to studies exploring the application of statistics for fast and unbiased data analysis. For example, to eliminate subjectivity and reliably identify significant differences in HDX-MS measurements, null measurements were performed and compared the application of individual tests of significance with Bonferroni correction and globally estimated significance limit (ΔHX) to evaluate the risk (i.e., falsely classifying a difference as significant) and power (i.e., failing to classify a true difference as significant) associated with different statistical analysis approaches (Supplementary Table S7). Combining these two approaches, hybrid statistical analysis was suggested, based on volcano plots that simultaneously decreased the risk of false positives and retained superior power. However, as these methods are not directly applicable in a comparative analysis setup, the authors demonstrated adoption of a univariate two one-sided tests (TOST) equivalence testing method for biosimilarity assessment. Using this method, the group was able to statistically distinguish between 5% deglycosylated NISTmAb and its unmodified reference material (Hageman et al., 2021).

##### 2.2.2.3 Conformational Stability

Conformational stability is measured as the change in enthalpy due to variation in physical and chemical properties of a molecule as a function of temperature and/or time. Thermal denaturation by variable temperature (VT)-CD has been traditionally used to study conformational stability as well as folding/unfolding mechanisms (Levy et al., 2014; Halim et al., 2018; Hermosilla et al., 2019) (Supplementary Table S4). Over the years, differential scanning calorimetry (DSC) has gained popularity as a fast and sensitive tool to measure thermodynamic stability and thermal unfolding pattern, where conformational changes are reflected as changes in characteristic transition/melting temperatures (*T*m) of one or multiple protein domains (Visser et al., 2013; Magnenat et al., 2017; Lee et al., 2019) (Supplementary Table S4). In recent times, time-correlated single-photon counting (TCSPC) is being used as an orthogonal technique to DSC to measure fluorescence lifetime distributions by the time-resolved intensity decay of protein (López-Morales et al., 2015; Mendoza-Macedo et al., 2016; Cerutti et al., 2019) (Supplementary Table S4). Other orthogonal tools include a fast, robust, low volume technique, i.e., nano differential scanning fluorimetry (nanoDSF) based on changes in intrinsic fluorescence upon thermal denaturation in a label-free fashion (Joshi et al., 2020; Wen et al., 2020) and IM-MS with collision-induced unfolding (CIU) (Kang et al., 2020b; 2020c) with limited applications so far (Supplementary Table S4).

#### 2.2.3 Glycosylation

Glycosylation is an enzymatic PTM that occurs in proteins of eukaryotic origin (mAbs, Fc-fusion proteins, and others such as epoetin-α). These exist in multiple glycoforms and exhibit complex micro-as well as macro-heterogeneity (Higel et al., 2016). A multi-level characterization strategy is generally adopted for glycan profiling, i.e., glycoprotein, glycopeptides, and released glycan levels that employs different LC-MS approaches with adaptations in sample preparation (reduction/digestion/derivatization) and mode of chromatography [RP/hydrophilic interaction chromatography (HILIC)] to suit the analyte size (intact/reduced/glycopeptide/released glycan) (Krull et al., 2020; Duivelshof et al., 2021) (Figure 4). Glycan characterization at the intact and reduced level follows a typical LC-MS routine workflow with minimal sample preparation. Released glycan analysis on the other hand is a fairly complex exercise with multiple steps in sample preparation followed by acquisition and detection through HILIC-FLD/MS. In earlier biosimilarity assessment publications, traditional labels, i.e., 2-aminobenzoic acid (2AA), 2-aminobenzamide (2AB), or 4-amino-N-(2-diethylaminoethyl) benzamide (procainamide) have been used (Lauber et al., 2015; Reusch et al., 2015) for sample preparation. The most common label that has been used in biosimilarity studies published so far is 2AB (Supplementary Table S4). In recent studies, use of novel commercial labels such as Glycoworks™ or *Rapi*Fluor-MS™ with superior performance in terms of speed, sample preparation, fluorescence (FL) signal, and ionization efficiency have been used (Keser et al., 2018). Bioimilarity studies using RapiFluor-HILIC-FLD/MS for different biosimilar candidates include Celltrion’s infliximab (Inflectra^®^) (Fang et al., 2016; Duivelshof et al., 2021), Shanghai Henlius’s rituximab (HLX01) (Xu et al., 2019), and trastuzumab (HLX02) (Xie et al., 2020), Biocad’s rituximab (Acellbia^®^), where 32 glycoforms were detected by tandem MS, while only 13 glycoforms were detected by HILIC-FLD (Kang et al., 2020c), and China’s ziv-aflibercept (Shen et al., 2021) (Supplementary Table S4).

For quantification of individual monosaccharides, High-performance anion-exchange chromatography-pulsed amperometric detection (HPAEC-PAD) has been the preferred technique (Bruggink et al., 2005), followed by RP-FLD after labeling with 1, 2-diamino-4, 5-methylenedioxy-benzenedihydrochloride (DMB) or fluorescein (Higel et al., 2013). Biosimilarity studies using HPAEC-PAD and RP-FLD have been detailed in the Supplementary Table S4.

For most chromatography-based analysis (irrespective of the attribute), Capillary Electrophoresis (CE) is often the popular orthogonal technique of choice. Recent advances in various CE modes have made it more user-friendly and versatile for glycan analysis. However, LC remains the preferred separation technique in similarity assessments. Examples of similarity assessments with orthogonal use of CZE (8-aminopyrene-1, 3, 6-trisulfonate, APTS labeling) and laser-induced fluorescence (LIF) detection to HILIC-FLD include China’s etanercept (company name not mentioned in associated publication) (Tan et al., 2012) and Probiomed’s trastuzumab (López-Morales et al., 2015) and 10 different mAbs (Giorgetti et al., 2018). The applicability of capillary gel electrophoresis (CGE) for quantitative glycosimilarity assessment has been demonstrated for etanercept biosimilars (Borza et al., 2018) (Supplementary Table S4).

The high cost *per* analysis, long analysis time, and a need for sophisticated instrumentation as well as skill set required for data analysis have increased the need for orthogonal tools for glycan profiling. To address this, there has been an increase in the exploration of simpler glycan analysis formats utilizing spectroscopic tools such as RAMAN and FTIR wherein minimal sample preparation is required and chemometric methods are applied for data analysis. In one such study, RAMAN was used as a Process Analytical Technology (PAT) tool for monitoring glycosylation site occupancy in CHO cell cultures in real-time, indicating method developability for similarity assessment as a possibility in near future (Li et al., 2018) (Supplementary Table S7). In another recent study the utility of FTIR has been demonstrated for inter-batch or inter-sample comparison of monosaccharide profiles (Derenne et al., 2020) (Supplementary Table S7). The methodology is based on a statistical (PCA) comparison of FTIR spectra (4,000 and 600 cm^−1^) of buffer exchanged glyco-therapeutics (to remove noise from excipients). The group mapped the FTIR fingerprint of 17 mAbs and found that it is not only sensitive to large differences such as the presence or absence of several monosaccharides but also to smaller modifications of the glycan and monosaccharide content. Another spectroscopic tool proposed for glycan profiling is NMR. In a proof of concept study, a “middle-down” NMR approach was conducted for identification of domain-specific glycosylation of mAbs without cleavage of the glycan moieties *via* sub-unit analysis of denatured Fc domain (Peng et al., 2018) (Supplementary Table S7). Chemical shift assignments from commercial standard glycans were obtained at ^13^C natural abundance and allowed for unambiguous determination of the chemical structure, glycosidic linkage position, and anomeric configuration of each monosaccharide in the major N-glycan scaffolds found in mAb molecules.

Another alternative/orthogonal glycan identification technique is lectin-microarray. Lectins are naturally occurring carbohydrate-binding proteins with affinities for specific sugar groups and due to this property, have found recent commercial application in glycan profiling for biotherapeutics. In a study, the utility as well as a high degree of orthogonality of commercial GlycoScope lectin microarray kit to three standard released glycan methods (HILIC-FLD of 2AA and 2AB labeling, HPAEC-PAD) have been demonstrated in an inter-lot comparability study (Cook et al., 2015) (Supplementary Table S7). The microarray platform comprised the microarray kit (microarray slides with Cy3-labelled goat anti-human-Fc antibody, IgG calibration standard, and components to prepare antibody exposure solution and bind/wash and block solutions) along with companion software for data interpretation and analysis.

Analysis of released glycans requires specific curated libraries for accurate annotation. Here, we would like to highlight two studies dealing with glycan data management, interpretation and analysis. The first study talks about the orthogonal application for structural annotation of N-glycan in a capillary electropherogram using GUcal software (Jarvas et al., 2015) (Supplementary Table S7). The second study describes a novel software, namley MoFi, which integrates hybrid MS data (intact and glycopeptide level) to assign glycan and PTMs to deconvoluted intact protein spectra (Skala et al., 2018) (Supplementary Table S7). The software first determines all monosaccharide/PTM compositions that are consistent with the residual masses derived from a deconvoluted spectrum of an intact glycoprotein, thereafter combining these primary annotations with a site-specific glycan library, generated through peptide mapping experiments. The software is unique in its data utilization approach and is capable of adding a new dimension of information to routine intact mass analysis, thereby making it more informative. The authors acknowledge the suitability of MoFi in GMP regulated biopharmaceutical analysis and it would indeed be interesting to evaluate its performance for biosimilarity assessment. Both MoFi and GUcal are freely available online.

#### 2.2.4 Product-Related Variants and Impurities

Product-related variants and impurities corresponds to heterogeneities formed during bioprocess manufacturing, handling, and storage including size-based heterogeneities (aggregates, fragments, and sub-visible/visible particles), charge based heterogeneities (acidic and basic variants), and other product modifications (reduced, oxidized, glycated, misfolded proteins, etc.). Impurity profiling is a prerequisite during biosimilar development and specifications are set vis-à-vis the innovator for product-related variants subjected to case-by-case evaluation depending on the nature of the variant (EMA, 1999).

##### 2.2.4.1 Aggregates/Fragments

Aggregation/fragmentation occur due to protein unfolding of hydrophobic patches with environmental changes during various stages of the manufacturing process and may elicit immunogenic responses if present in significant amounts (Ratanji et al., 2014). The spectrum of aggregate size ranges between soluble aggregates to visible precipitates, depending upon exposure to various stresses (i.e., shear, thermal, chemical, freeze-thaw, etc.) and duration of exposure. Hence, its assessment involves the use of multiple orthogonal tools (Figure 4). Size exclusion chromatography (SEC)-UV is the method of choice. It can quantify soluble aggregates/fragments (1–100 nm) and offers rapid analysis, excellent resolution, robustness, and reproducibility. More recently SEC has been augmented with multi-angle light scattering (MALS) for measuring the size, i.e., molar mass and root-mean-square (RMS) radius (also called the radius of gyration, *R*_g_) of the different molecular species separated by SEC (Flores-Ortiz et al., 2014; Lee K. H. et al., 2018; Singh et al., 2018; Seo et al., 2020).

Protein loss due to stationary phase interactions and salt-induced aggregation/dissociation are common issues observed during SEC analysis. Hence, sedimentation velocity-analytical ultracentrifugation (SV-AUC), a matrix-free alternative to SEC, has been employed to quantitatively measure the size distribution. Researchers have proposed a validated AUC method and demonstrated its robustness as an orthogonal tool for similarity assessment of rituximab biosimilar product (Patil et al., 2020). However, AUC is not yet considered a technology that can be used for routine analysis, due to expensive instrumentation as well as challenging and time-intensive methods. While sodium dodecyl sulfate-polyacrylamide gel electrophoresis (SDS-PAGE) is still being used, it is qualitative in nature with limited sensitivity. CE-SDS, an automated version of SDS-PAGE in capillary format, is an orthogonal technique to SEC, particularly useful in quantifying fragments, partially reduced and non-glycosylated proteins (Shi et al., 2012; Wagner et al., 2020). Similarity studies with usage of SEC-UV/MALS, SV-AUC and CE-SDS include biosimilar candidates of Celtrion’s infliximab (Remsima^®^) (Jung et al., 2014), trastuzumab (CT-P6) (Lee J. et al., 2018) and rituximab (CT-P10: Truxima™) (Lee K. H. et al., 2018), and Amgen’s adalimumab (ABP 501) (Liu et al., 2016), bevacizumab (ABP 215) (Seo et al., 2018), infliximab (ABP 710) (Saleem et al., 2020), rituximab (ABP 798) (Seo et al., 2020) and eculizumab (ABP 959) (Hutterer et al., 2021) (Supplementary Table S4).

During SEC, insoluble aggregates/particles either elute out through the void volume or retain on the pre-column filter, hence needs to be analyzed using other techniques. These particles are categorized into sub-visible (1–100 μm) and sub-micron (100 nm-1 μm) particles depending on particle size. Sub-visible particles are analyzed by light obscuration (LO) in high accuracy (HIAC) liquid particle counter and micro-flow imaging (MFI). LO, an indirect optical method, is used to measure particle size distribution (PSD) of particles >10 μm but underestimates smaller transparent particle populations, and cannot distinguish sub-populations (Narhi et al., 2009). Hence, more recently MFI is preferably used either singly or in conjunction with LO as an orthogonal technique for result validation (Sharma et al., 2010). Sub-micron particles are analyzed by dynamic light scattering (DLS) and field flow fractionation (FFF) or asymmetrical field flow fractionation (AF4). DLS is a semi-quantitative tool that determines PSD in the 1 nm–5 μm range, but cannot measure accurate size (Ahrer et al., 2003). AF4 is a no matrix alternative tool orthogonal to SEC-UV/MALS, and DLS for aggregates >1 nm which can be coupled with various detectors and enable analysis under native conditions (Contado, 2017). Few similarity studies have used all of the above multiple orthogonal platforms, i.e., LO, MFI, DLS and FFF in conjunction with SEC-UV/MALS, AUC, CE-SDS, for aggregate analysis. Examples include Amgen’s adalimumab (ABP 501) (Liu et al., 2016), bevacizumab (ABP 215) (Seo et al., 2018), trastuzumab (ABP 980) (Hutterer et al., 2019), infliximab (ABP 710) (Saleem et al., 2020), and rituximab (ABP 798) (Seo et al., 2020) (Supplementary Table S4).

In addition to the established repertoire of tools above mentioned, DOSY-NMR has been demonstrated to be applicable for insulin and mAb-based formulations (Patil et al., 2017; Joshi et al., 2021). For mAbs, the relevance of DOSY-NMR for realistic hydrodynamic measurement has been demonstrated, where size estimations were found to be closer to computationally calculated radii of the published X-ray diffraction structures on Fab and Fc (Joshi et al., 2021) (Supplementary Table S7). Due to the highly heterogeneous nature of HMWs (in terms of shape and size), species-specific quantification is challenging (Hermosilla et al., 2020) (Supplementary Table S7). As a possible solution, researchers have developed a semi-automated electron microscopy (EM)-based method that involves semi-automated, size-based clustering of different protein species from micrographs. Demonstrating its applicability on mAbs, the method was shown to automatically select a highly heterogeneous population of aggregates for a given sample and perform a size-based classification (number of aggregates of each species vs size of radius for the species) (Kumar et al., 2020) (Supplementary Table S7). Nanoparticle tracking analysis (NTA), a non-destructive, high resolving real-time monitoring technique to measure the number-based PSD of particles >30 nm, has found limited mention in similarity studies thus far (Moreno et al., 2016; Arvinte et al., 2019) (Supplementary Table S7).

##### 2.2.4.2 Charge Variants

Charge variants are differently charged proteoforms formed in different colloidal matrices (i.e., culture media, in-process buffers, or formulation) during various stages of the manufacturing process. Charge variants are considered key-quality attributes (kQAs) and an ongoing scientific debate is prominent on its inclusion as a CQA (DBT, 2016; Singh et al., 2016). Over the years, cation exchange (CEX) chromatography has been the preferred tool for charged variant analysis as it offers rapid analysis, suitable resolution, robustness, and reproducibility (Joshi et al., 2015). Few similarity studies include Amgen’s bevacizumab (ABP 215) (Seo et al., 2018), rituximab (ABP 798) (Seo et al., 2020) and infliximab (ABP 710) (Saleem et al., 2020). Similar to other chromatography based analysis, CE serves as a rapid, high-performance alternative tool orthogonal to CEX (Moritz et al., 2015) and has been employed in biosimilarity assessments such as for Sun Pharma’s rhCG (SB005) (Thennati et al., 2018) and rituximab (SB-02) (Singh et al., 2018) (Supplementary Table S4). Further, the application of different modes of CE and hyphenation of CEX or CE with MS, have introduced more orthogonal tools to the analytical armory with respect to charge variant analysis (Gahoual et al., 2014; Haselberg et al., 2018). Capillary isoelectric focusing (cIEF), an extension to gel IEF in a capillary format, offers a pI based separation of charged species in a pH gradient in response to an electric field with higher resolution, lesser sample volume requirement, and faster sample analysis (Zhao and Chen, 2014; Suba et al., 2015). Image capillary isoelectric focusing (icIEF) allows the cIEF process to be “imaged” in real-time using whole-column imaging detection (WCID) technology. While, cIEF has been employed for charge profiling of biosimilar candidates of Amgen’s adalimumab (ABP 501) (Liu et al., 2016) and bevacizumab (ABP 215) (Seo et al., 2018), and Probiomed S.A. de C.V.’s rituximab (Kikuzubam) (Miranda-Hernandez et al., 2015), etanercept (Infinitam^®^) (Miranda-Hernández et al., 2016), trastuzumab-Probiomed (López-Morales et al., 2015) and infliximab-Probiomed (Velasco-Velázquez et al., 2017); examples of biosimilarity platforms with icIEF include Pfizer’s infliximab (PF-06438179) (Derzi et al., 2016) and adalimumab (PF-06410293) (Derzi et al., 2020), Zhejiang Hisun’s tocilizumab (HS628) (Miao et al., 2017), Samsung Bioepis’s infliximab (SB2) (Hong et al., 2017) and adalimumab (SB5) (Lee et al., 2019), and Shanghai Henlius’s rituximab (HLX01) (Xu et al., 2019), trastuzumab (HLX02) (Xie et al., 2020) and adalimumab (HLX03) (Zhang et al., 2020) (Supplementary Table S4).

Hyphenation of CEX/CE with MS either offline or *via* modifying the buffer system to use MS-compatible volatile salts (ammonium acetate/formate) has expanded the information gained by CEX/CE alone (Biacchi et al., 2015). The low flow operation of the microfluidic systems for CE-MS significantly boosts MS sensitivity and increased the dynamic range, even with sample amounts as low as 1 ng. Recently, 2 studies by Carillo et al., and Fussl et al., have demonstrated the application of commercially available microfluidic ZipChip microfluidic CE-MS technology for characterization of native charge variant profile complex mAbs such as cetuximab (Carillo et al., 2020; Fussl et al., 2020). Within the 1D-LC analysis format, there has been an increase in the development of MS-compatible CEX methods and direct online coupling of CEX to MS using volatile salt-based pH gradients, giving rise to Native CEX-MS. This has been demonstrated by multiple groups with variations in the stationary phase (weak and strong ion exchangers) and MS platforms (ESI-MS, IM-MS, and orbitrap) for trastuzumab, adalimumab, infliximab, bevacizumab, and cetuximab (Bailey et al., 2018; Füssl et al., 2018; Sankaran et al., 2018; Jaag et al., 2021; Murisier et al., 2021) (Supplementary Table S7).

Multidimensional platforms such as 2D-LC with CEX in first dimension followed by an MS-compatible second dimension, i.e., RP (desalting improves peak capacity and MS compatibility) have also been incorporated in biosimilarity (Alvarez et al., 2011; Stoll et al., 2015) (Supplementary Table S7). More such applications for similarity assessment of biosimilars have also been shown for cetuximab, trastuzumab, and infliximab (Sorensen et al., 2016).

##### 2.2.4.3 Other Product Modifications

Non-enzymatic PTMs include modifications such as oxidation, phosphorylation, sulfation, acetylation, methylation, and hydroxylation, that are formed during multiple stages of the manufacturing process. Liquid chromatography offers unparalleled selectivity towards the characterization of PTMs and the quantitation of related molecular variants and impurities. RP-HPLC with UV/FLD is the preferred technique for quantifying oxidized and reduced species (Brokx et al., 2017; Bor Tekdemir et al., 2020). It has also been efficiently coupled with MS platforms for site-specific identification and relative quantification (Lee K. H. et al., 2018; Saleem et al., 2020). Hydrophobic interaction chromatography (HIC) (Flores-Ortiz et al., 2014; Hassett et al., 2018) with UV/FLD for molecular variants, i.e., oxidized, deamidated with isomerization and/or succinimide formation, proteolytic fragments and misfolded species, and boronate affinity chromatography (BAC) (Visser et al., 2013; Singh et al., 2018) with FLD for glycated species have been less widely used for the separation of product-related variants (Supplementary Table S4). It must be noted that HIC is highly suitable to monitor oxidation, but for deamidation/isomerization, it is much less efficient than IEX.

#### 2.2.5 Process-Related Variants

Process-related variants, also called process residuals, include cell substrates, e.g., HCPs, HCD, cell culture, and downstream processing residuals. Enzyme-linked immunosorbent assay (ELISA) and real-time or quantitative PCR (qPCR using SYBR green, PicoGreen) have been consistently used as the method of choice for HCP and HCD detection and quantitation, respectively, in biosimilarity studies due to its high sensitivity, high throughput, and relative ease of use (Magalhaes et al., 2016; Xu et al., 2019; Xie et al., 2020) (Supplementary Table S4). Few publications have listed Threshold™ assays for HCD detection of filgrastim (EP2006; ≤200 pg/mg) (Sörgel et al., 2015) and adalimumab (FKB327; <2 pg/mg) (Schreiber et al., 2020) biosimilars (Supplementary Table S4).

Specific to HCP analysis, orthogonal methods are coming into picture, since identification of individual HCPs is gaining importance in addition to measuring the overall amount of the HCPs Due to the complex nature of the HCP mixture, qualitative methods such as ELISA are being replaced in favor of proteomic techniques such as 2D-gel electrophoresis and LC-MS/MS-based platforms. In two studies, three orthogonal methods, i.e., ELISA, 2D-PAGE/DIGE, and LC-MS/2D-LC-MS^E^ were used to identify, quantify and compare HCPs present in in-house filgrastim (Rathore and Bhambure, 2014) and Amgen′s adalimumab (ABP 501) (Liu et al., 2016) biosimilars with respect to the innovators (Supplementary Table S4). Due to the limited separation efficiency of LC-based systems, 2D-LC (high-pH RP/low-pH RP)-IM-MS^E^ platform have been explored for comparing HCP profiles between Celltrion’s infliximab biosimilar (Inflectra^®^) and Remicade^®^ especially significant for co-eluting peptides (Fang et al., 2016) (Supplementary Table S4). Finally, orthogonal LC-MS platforms such as CZE-ESI-MS/MS have been successful in identification of a greater number of HCPs compared to LC-MS/MS (Zhu et al., 2016; Kumar et al., 2021).

##### 2.2.5.1 Statistics in Analytical Biosimilarity Assessment

While the analytical biosimilarity platforms have seen significant evolution over the years with respect to the techniques employed and orthogonal assessment of CQAs, data analysis has remained traditional for a large part, depending on comparison of information in the form of X-Y plots and charts. Recent conversations on statistical inference of biosimilarity have opened following USFDA’s, now retracted draft guidance on “Statistical Approaches to Evaluate Analytical Similarity.” The draft guidance suggested risk and criticality-based segregation of CQAs into tiers and the application of tier-based statistical tools for ascertaining confidence in similarity for a given analytical dataset (USFDA, 2017). Application of tier-based statistical assessment has thereafter been demonstrated in recent biosimilarity assessments of biosimilar candidates for Celltrion’s trastuzumab (CT-P6) (Lee J. et al., 2018), Samsung Bioepis’s adalimumab (SB5) (Lee et al., 2019) Shanghai Henlius’s rituximab (HLX01) (Xu et al., 2019), adalimumab (HLX03) (Zhang et al., 2020), and Roche China’s bevacizumab (BVZ-BC) (Yu et al., 2020) (Supplementary Table S4). Along the same line of thought, statistical alternatives to similarity evaluation in cases where multiple reference products exist (EU-approved and US-licensed) have been proposed such as the use of simultaneous confidence approach as a possible means to reconcile the biosimilarity between the reference products as using it as a whole in comparison to the intended biosimilar (Zheng et al., 2019) (Supplementary Table S7). Studies comparing test procedures using confidence intervals for recently developed methods as well as other previously developed methods applied for demonstrating analytical biosimilarity have also been published (Burdick et al., 2017; Wang and Chow, 2017; Quiroz et al., 2019; Chen and Hsiao, 2020) (Supplementary Table S7). Comparative signature diagrams (CSDs) has been explored as an orthogonal visualization technique for analytical data sets in the form of colored contour plots (Kim et al., 2016) (Supplementary Table S7). However, these studies do not address orthogonal ways to analyze the raw data. As far as orthogonal strategies for raw data evaluation go, the application of multivariate data analysis (MVDA) techniques such as principal component analysis (PCA) has been applied to spectroscopy data from NMR and MS towards an unbiased spectral comparison of products (Arbogast et al., 2017; Chen et al., 2018; Shatat et al., 2018; Hassan et al., 2019; Brinson et al., 2020; Wang et al., 2020) (Supplementary Table S7). What remains yet unexplored, is the application of advanced statistics towards a holistic, multi-technique, multi-attribute biosimilarity assessment that also incorporates the impact of criticality of a CQA on relevance of a given data. In addition, application of statistics towards a scoring strategy for biosimilars has also yet to be demonstrated. Some examples of biosimilar scoring strategies limited to single instrument data have been published in recent times. These include a study where multivariate data analysis method based on JMP^®^ software (SAS Institute Inc.) was developed to assess the glycosylation pattern similarity of antibody candidates from different conditions and scoring was done based on specific distance between the biosimilar and the reference product (Xu et al., 2021) (Supplementary Table S7). Another, single attribute-based scoring strategy has been used to list the different glycosylation related CQAs (gCQAs) and then describe calculations for establishing two different similarity scores, namely profile similarity score and compositional similarity score. The mean of these two scores has been defined as the final Glycosimilarity Index (GI) (Szekrenyes et al., 2020) (Supplementary Table S7).

## 3 Conclusion

Biosimilars are targeted to be an affordable alternative for expensive innovator biologics. However, reaching the goal of affordability without compromising with quality is challenging and requires a collaborative effort from manufacturers and regulators, guided by subject matter experts. For emerging biosimilar manufacturers and pharmaceutical manufactures that are expanding their offerings in the biosimilar domain, there is a steep learning curve with regards to successful manufacturing of biosimilars as translational learnings from the pharmaceutical sector are limited. Therefore, in the interest of affordability, it is important to build an informative corpus of common knowledge regarding all aspects of biosimilar development. This review is a step in that direction.

**FIGURE 6:**
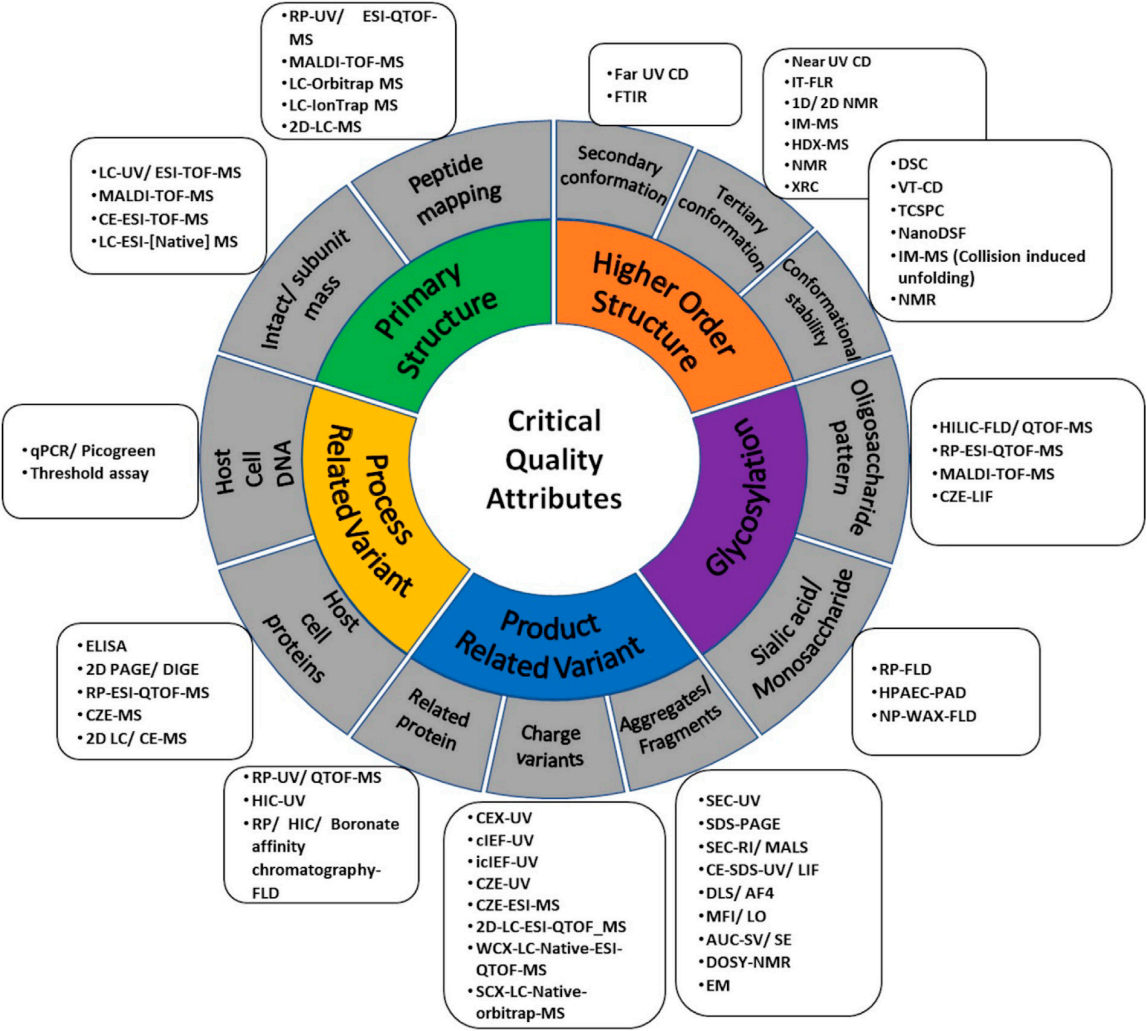
A comprehensive map of orthogonal analytical platforms for different Critical Quality Attributes (CQAs) i.e., primary structure, Higher Order Structure (HOS), glycosylation, product-related and process-related variant used in analytical similarity assessment.

Over the years, as blockbuster drugs reach patent expiry, there has been a steady increase in the number of biosimilar approvals in all the major jurisdictions, especially for mAbs. There has also been an increase in published biosimilarity assessment studies. However, it does not yet commensurate with the rate of approvals and more such assessments need to be published through peer-reviewed pathways. The number of approvals as well as published assessments is likely to go up as global guidelines on interchangeability evolve and there is more market acceptance for these products. The data presented in peer-reviewed similarity assessment studies are representative of the total data generated during a similarity exercise and thus a good indicator of the percolation of new technologies from R&D stage to incorporation in the analytical platform to increase complementarity or orthogonality. Putting together all the tools that have either been used in similarity assessments or for which application towards similarity has been indicated, a comprehensive map of analytical platform, including orthogonal tools, has been built as an outcome of the literature survey done in this review (Figure 6).

As there is a cost attached with the incorporation of any new technology in the similarity assessment platform, common/well established tools and techniques that can be applied across the CQA spectrum are likely to be preferred. So far, the evolution of similarity assessment platforms reflects a growing acceptance of hyphenated platforms (separation x detection) with MS being the prevalent end-point detector. This is followed by advancements in spectroscopy, such as the inclusion of NMR for whole molecule analysis.

Recent technological advancements with respect to the application of 2D-NMR, multidimensional chromatography, HDX-MS, and spectroscopy in the characterization of CQAs with several publications on method standardization of these techniques towards similarity assessment have populated the scholastic space. However, to facilitate affordability, future similarity assessment exercises need to be more lightweight with respect to the number of tools and more diverse and information-intensive with respect to characterization. A possible solution seems to emerge in the form of MAMs. Indeed, there is a growing trend towards establishing multi-attribute monitoring and analysis methods for process control and purity analysis. Current MAMs are MS-based and applicable on digested proteins and MAM developments in whole molecule assessments in formulation conditions *via* LC-MS or spectroscopy platforms are a welcome move for similarity assessments.

We hope that the repositories provided as tables in the main text as well as Supplementary Information acts as a useful go to source for biopharmaceutical scientists, academicians, interested regulators as well as manufacturers, for the purpose of designing analytical similarity assessment platforms, decision making on tools to invest in, common methodologies to be adopted and also for general understanding of biosimilar and similarity landscape.

## Footnotes

1 CAGR is a geometric progression ratio that provides a constant rate of return over the time period.

2 DP is the finished dosage form, for example, a tablet, capsule or solution that contains an active pharmaceutical ingredient, generally, but not necessarily, in association with inactive ingredients.
