# Supplementary material for: Analytical Similarity Assessment of Biosimilars: Global Regulatory Landscape, Recent Studies and Major Advancements in Orthogonal Platforms
Source: Front Bioeng Biotechnol. 2022 Feb 9;10:832059. doi: 10.3389/fbioe.2022.832059 (PMC8865741; doi:10.3389/fbioe.2022.832059)
Supplement: Supplementary file 1 [file DataSheet1.docx]

**Supplementary Information**

**Analytical similarity assessment of biosimilars: Global regulatory landscape, recent studies and major advancements in orthogonal platforms**

Neh Nupur^1#^, Srishti Joshi^1#^, Davy Gulliarme^2a, b^, Anurag S. Rathore^1*^

^1^Department of Chemical Engineering, IIT Delhi, Hauz Khas, New Delhi 110016 India

^2a^Institute of Pharmaceutical Sciences of Western Switzerland (ISPSO), University of Geneva, CMU-Rue Michel Servet 1, 1211 Geneva 4, Switzerland

^2b^School of Pharmaceutical Sciences, University of Geneva, CMU-Rue Michel Servet 1, 1211 Geneva 4, Switzerland

^*^Corresponding author:

Anurag S. Rathore

Department of Chemical Engineering

Indian Institute of Technology

Hauz Khas, New Delhi, 110016, India

Phone: +919650770650

asrathore@biotechcmz.com

www.biotechcmz.com

^#^N.N. and S.J. have contributed equally to this article and share first authorship.

**Methodology**

1. **Analytical similarity assessment in published literature**

To compile the list of publications from peer-reviewed journals that presented similarity assessments for approved/ intended biosimilars, Scopus® database search was performed using the advanced document search function (Search parameters: biosimilar, analytical, similarity, search within - all fields, language - English). From the initial search results, abstracts were screened for applicability, and articles on physicochemical (with or without functional) similarity assessment of approved/ intended biosimilars were included in the final database. Only original articles were selected where - the full-text article was available, an approved/ intended biosimilar was studied, and a similarity assessment exercise was presented. Final database consisted of 116 publications tabulated in Supplementary information Table S4.

1. **Recent advancements and orthogonality in analytical similarity assessment**

To cover the improvement in state-of-art analytical platforms and advancement in orthogonal tools published between 2015-2021, SCOPUS database search was performed using the advanced document search function (Search parameters: biosimilar AND comparability OR similarity, search within - all fields, language - English, date range - 2015 to 2021). A total of 1352 publications were listed in response to the above search query. From the initial search results, routine similarity assessment papers, papers pertaining to pharmacoeconomics and regulatory aspects, reviews, commentaries and no method-based research articles were removed. The content of remaining papers was screened for topic applicability and papers wherein the method described is either applied for similarity assessment or the authors have acknowledged the applicability of the method for similarity assessment were included. Publications where the method described has been used for comparability assessment (note: similarity assessment refers to biosimilarity between and originator and a biosimilar whereas comparability assessment refers to characterization of changes in biologic profile in response to process change) or the method is suitable for comparative analysis were also included in creation of the database. There have also been publications covering orthogonal methods for data analysis using chemometrics or machine learning. Such papers have also been included as these publications tackle recent concepts related to quantitative measurement through qualitative data, similarity matrices and similarity indices for either one attribute or for the whole characterization data. The final database consists of information from 98 publications. From this database, information regarding attribute characterized, technique used, method details, known orthogonality and drug substance characterized was extracted from each publication and tabulated in Supplementary information Table S7.

**Table S1: List of abbreviations**

| 1D | One Dimensional |
| --- | --- |
| 2° | Secondary |
| 2AA | 2-aminobenzoic acid |
| 2AB | 2-aminobenzamide |
| 2D-DIGE | Two-Dimensional Difference Gel Electrophoresis |
| 2D-LC | Two-Dimensional Liquid Chromatography |
| [2D-PAGE](https://www.future-science.com/doi/10.2144/000112823) | Two-Dimensional Polyacrylamide Gel Electrophoresis |
| 3° | Tertiary |
| 3D | Three-Dimensional |
| AEX/ CEX | Anion/ Cation Exchange Chromatography |
| AF4 | Asymmetrical Field Flow Fractionation |
| APTS | 8-Aminopyrene-1, 3, 6-Trisulfonate |
| ATR-FTIR | Attenuated Total Reflection-Fourier Transform Infrared Spectroscopy |
| BAC | Boronate Affinity Chromatography |
| BLA | Biologic License Application |
| BPCI | Biologics Price Competition and Innovation Act |
| CAGR | Compound Annual Growth Rate |
| CD | Circular Dichroism Spectroscopy |
| CDSCO | Central Drugs Standard Control Organization |
| CE | Capillary Electrophoresis |
| CGE | Capillary Gel Electrophoresis |
| CHO | Chinese Hamster Ovary |
| CID | Collision-Induced Dissociation |
| cIEF | Capillary Isoelectric Focusing |
| CIU | Collision-Induced Unfolding |
| CQA | Critical Quality Attributes |
| CSD | Comparative Signature Diagrams |
| CZE | Capillary Zone Electrophoresis |
| DLS | Dynamic Light Scattering |
| DMB | 1,2-Diamino- 4,5-Methylenedioxybenzene |
| DNPH GRP | Dinitrophenylhydrazine group |
| DOSY | Diffusion Ordered Spectroscopy |
| DP | Drug Product |
| DSC | Differential Scanning Calorimetry |
| EGFR | Epidermal Growth Factor Receptor |
| ELISA | Enzyme-Linked Immunosorbent Assay |
| EMA | European Medicines Agency |
| ESI | Electrospray Ionization |
| ETD | Electron-Transfer Dissociation |
| EU | European Union |
| FFF | Field Flow Fractionation |
| FLD | Fluorescence Detection |
| FSH | Follicle Stimulating Hormone |
| FITC | Fluorescein Isothiocyanate |
| FTICR | Fourier Transform Ion Cyclotron Resonance |
| GC | Gas Chromatography |
| gCQAs | Glycosylation-related Critical Quality Attributes |
| GCSF | Granulocyte Colony Stimulating Factor |
| GI- | Glycosimilarity Index |
| GMP | Good Manufacturing Practice |
| HCD | Higher-Energy C-Trap Dissociation |
| HCD | Host cell DNA |
| HCP | Host cell protein |
| HDMS/ HRMS | High Definition/ High Resolution-Mass Spectrometry |
| HDX-MS | Hydrogen-Deuterium Exchange-Mass Spectrometry |
| HER2 | Human Epidermal Growth Factor Receptor 2 |
| HESI | Heated Electrospray Ionization |
| HIC | Hydrophobic Interaction Chromatography |
| HILIC | Hydrophilic Interaction Chromatography |
| HMQC | Heteronuclear Multiple Quantum Coherence |
| HMWs | High Molecular Weight Species |
| HOS | Higher-Order Structure |
| HPAEC | High-Performance Anion-Exchange Chromatography |
| HPLC | High-Performance Liquid Chromatography |
| HSQC | Heteronuclear Single Quantum Coherence |
| iCE | Imaged Capillary Electrophoresis |
| ICH | International Council for Harmonization |
| icIEF | Imaged Capillary Isoelectric Focusing |
| IdeS | Immunoglobulin G-degrading enzyme of Streptococcus pyogenes |
| IEF | Isoelectric Focusing |
| IEX | Ion Exchange Chromatography |
| IFNAR1/ IFNAR2c | Interferon Alpha and Beta Receptor Subunit 1/2 |
| IgE/ IgG | Immunoglobulin E/ G |
| IM-MS | Ion Mobility-Mass Spectrometry |
| IP-RP-AIF-IM-MS | Ion Pair-Reversed Phase-All Ion Fragmentation-Ion Mobility-Mass Spectrometry |
| ITC | Isothermal Titration Calorimetry |
| IT-FLR | Intrinsic Fluorescence Spectroscopy |
| JAK-STAT | Janus Kinase/ Signal Transducers and Activators of Transcription |
| kQA | Key Quality Attributes |
| LC | Liquid Chromatography |
| LIF | Laser-Induced Fluorescence Detection |
| LM | Light Microscopy |
| LO HIAC | Light Obscuration in High Accuracy Liquid Particle Counter |
| mAbs | Monoclonal Antibodies |
| MALDI | Matrix Assisted Laser Desorption/ Ionization |
| MALS | Multi-Angle Light Scattering |
| MAM | Multi-Attribute Methods |
| MFDS | Ministry of Food and Drug Safety |
| MFI | Micro-Flow Imaging |
| ML | Machine Learning |
| MRM | Multiple Reaction Monitoring |
| MS^E^ | Tandem Mass Spectrometry |
| MVDA | Multivariate Data Analysis |
| nanoDSF | Nano Differential Scanning Fluorimetry |
| NIST | National Institute of Standards and Technology |
| NMPA | National Medical Products Administration |
| NMR | Nuclear Magnetic Resonance Spectroscopy |
| NOESY | Nuclear Overhauser Effect Spectroscopy |
| NP | Normal Phase Chromatography |
| NRAs | National Regulatory Authorities |
| nr/ rCE-SDS | Non-Reduced/ Reduced Capillary Electrophoresis Sodium Dodecyl Sulfate |
| NRM | Nile Red Microscopy |
| NTA | Nanoparticle Tracking Analysis |
| PAD | Pulsed Amperometric Detection |
| PAT | Process Analytical Technology |
| PCA | Principal Component Analysis |
| PD | Pharmacodynamics |
| PGSTE | Pulsed-Field Gradient Stimulated Echo |
| PHS | Public Health Service Act |
| pI | Isoelectric Point |
| PK | Pharmacokinetics |
| PMDA | Pharmaceuticals and Medical Devices Agency |
| PSD | Particle Size Distribution |
| PTH | Parathyroid Hormone |
| PTMs | Post-Translational Modifications |
| QC | Quality Control |
| QIM-MS | Quadruple Ion Neutral-Mass Spectrometry |
| qPCR | Real-Time/ Quantitative Polymerase Chain Reaction |
| QQQ | Triple Quadrupole |
| QTOF | Quadrupole Time-of-Flight |
| R&D | Research and Development |
| RANKL | Receptor Activator of Nuclear Factor Kappa-Β Ligand |
| RCGM | Review Committee on Genetic Manipulation |
| rDNA | Recombinant DNA |
| r-FVIIa | Recombinant-Human Factor VIIa |
| r-hCG | Recombinant-Human Chorionic Gonadotropin |
| r-hGH | Recombinant-Human Growth Hormone or Somatotropin |
| RI | Refractive Index |
| RMS | Root‐Mean‐Square |
| RP | Reverse Phase Chromatography |
| SAXS | Small Angle X-Ray Scattering |
| SBPs | Similar Biotherapeutic Products |
| SCX | Strong Cation Exchange Chromatography |
| SDS-PAGE | Sodium Dodecyl Sulfate-Polyacrylamide Gel Electrophoresis |
| SE/ SV-AUC | Sedimentation Equilibrium/ Sedimentation Velocity-Analytical Ultracentrifugation |
| SEC | Size Exclusion Chromatography |
| SELDI | Surface-Enhanced Laser Desorption/ Ionization |
| SH | Sulfhydryl group |
| SILAC | Stable Isotope Labeling by/ with Amino Acids in Cell Culture |
| SLS | Static Light Scattering |
| TCSPC | Time-Correlated Single-Photon Counting |
| TEM | Transmission Electron Microscopy |
| TGA | Therapeutic Goods Administration |
| ThT | Thioflavin T |
| TNFα | Tumour Necrosis Factor Alpha |
| TOCSY | Total Correlation Spectroscopy |
| TOF | Time-of-Flight |
| TOST | Two One-Sided Tests |
| UHD/ UHR | Ultra-High Definition/ Ultra-High Resolution |
| UPLC | Ultra-Performance Liquid Chromatography |
| URT | Ultrasound Resonance Technology |
| US | United States |
| USFDA | United States Food and Drug Administration |
| UV | Ultraviolet |
| [UVPD](https://assets.thermofisher.com/TFS-Assets/CMD/brochures/sn-65629-ms-uvpd-orbitrap-tribrid-sn65629-en.pdf) | Ultraviolet Photodissociation |
| VEGF | Vascular Endothelial Growth Factor |
| VT-CD | Variable Temperature-Circular Dichroism |
| WAX | Weak Anion Exchange Chromatography |
| WCID | Whole Column Imaging Detection |
| WHO | World Health Organization |
| XRC | X-Ray Crystallography |
| XRD | X-Ray Diffraction |

**Table S2: List of Definitions**

| **S. No.** | **Term** | **Definitions/ Meaning** |
| --- | --- | --- |
| 1 | Biopharmaceutical | Biological macromolecule or cellular component, such as a blood product, used as a pharmaceutical. |
| 2 | Biosimilar | Denoting or relating to a biopharmaceutical drug designed to have active properties similar to those of a drug that has previously been licensed. |
| 3 | Blockbuster drug | Drugs that generate an annual revenue of $1 billion for the company that sells it |
| 4 | Charge variant | Multiple variants of the same protein with differences in charge |
| 5 | Chemometrics | Application of statistics to the field of chemical analysis. |
| 6 | Chinese hamster ovary cells | An epithelial cell line derived from the ovary of the Chinese hamster, used in production of recombinant therapeutic proteins |
| 7 | Chromatography | A technique for the separation of a mixture by passing it in solution or suspension through a medium in which the components move at different rates |
| 8 | Critical Quality Attribute | A CQA is a physical, chemical, biological, or microbiological property or characteristic that should be within an appropriate limit, range, or distribution to ensure the desired product quality. |
| 9 | Disulfide linkage | Covalent bond between sulfur atoms that binds two peptide chains or different parts of the same peptide chain |
| 10 | Drug product | Drug product means a finished dosage form, for example, tablet, capsule, solution, etc., that contains an active drug ingredient generally, but not necessarily, in association with inactive ingredients. The term also includes a finished dosage form that does not contain an active ingredient but is intended to be used as a placebo. |
| 11 | Electrophoresis | The movement of charged particles in a fluid or gel under the influence of an electric field. |
| 12 | Innovator biologic | First drugs created containing its specific active ingredient to receive approval for use |
| 13 | Liquid chromatography | A chromatographic separation technique in which mobile phase is liquid |
| 14 | Mass spectroscopy | An analytical technique that is used to measure the mass-to-charge ratio of ions |
| 15 | Multidimensional chromatography | A chromatographic technique which is capable of delivering heightened separation performance due to sample separation through multiple separation stages |
| 16 | Orthogonal technique | An additional method that provides very different selectivity to the primary method and is used to evaluate the primary method. |
| 17 | Patent Cliff | A patent cliff refers to patent expiration of a specific product. |
| 18 | Peptide mapping | An identity test for proteins that involves enzymatic treatment of the protein resulting in small peptide fragments that are subsequently analyzed and identified through mass spectrometry |
| 19 | Post-translational modifications | Refers to the covalent and enzymatic modification of proteins following protein biosynthesis (translation) |
| 20 | Process Analytical Technology | A mechanism to design, analyze, and control pharmaceutical manufacturing processes through the measurement of critical process parameters (CPP) which affect critical quality attributes |
| 21 | Secondary structure | The local three-dimensional structure of sheets, helices, or other forms adopted by a polynucleotide or polypeptide chain, due to electrostatic attraction between neighboring residues. |
| 22 | Spectroscopy | Branch of science that deals with investigation and measurement of spectra produced when matter interacts with or emits electromagnetic radiation |
| 23 | Tertiary structure | The overall three-dimensional structure resulting from folding and covalent cross-linking of a protein or polynucleotide molecule. |

**Table S3: List of approved biosimilars, non mAbs and mAbs in major countries/ regions (Till July 2021)**

| **Non mAbs** | **filgrastim** | **peg-filgrastim** | **teriparatide** | **human insulin** | **insulin glargine** | **epoetin alfa** | **interferon**  **alfa-2b** | **interferon**  **beta-1a** | **follitropin**  **alfa** | **darbepoetin alfa** | **r-hCG** | **somatropin** | **others** |
| --- | --- | --- | --- | --- | --- | --- | --- | --- | --- | --- | --- | --- | --- |
| Target | GCSF receptor | GCSF receptor | PTH type 1 receptor | Insulin receptor | Insulin receptor | JAK-STAT  binding receptor | IFNAR1  and  IFNAR2c receptors | IFNAR1  and  IFNAR2c receptors | FSH  receptor | Erythro-poietin  receptor | Luteinizing/  Chorio-  gonadotropin hormone  receptor | dimeric  growth  hormone receptor |  |
| EU | 7 | 8 | 4 |  | 2 | 4 |  |  | 2 |  |  | 1 | 7 |
| US | 2 | 4 | 1 |  | 3 | 1 |  |  |  |  |  |  | 1 |
| Canada | 2 | 3 | 3 |  |  |  |  |  |  |  |  | 1 | 4 |
| Australia | 3 | 3 |  |  | 2 |  |  |  | 1 |  |  | 2 | 4 |
| S. Korea |  |  | 1 |  | 2 | 1 |  |  |  | 1 |  | 1 | 0 |
| Japan | 3 |  | 1 |  | 2 | 1 |  |  |  | 5 |  | 1 | 1 |
| India | 9 | 8 | 3 | 6 | 5 | 11 | 4 | 1 | 3 | 4 | 2 | 2 | 12 |
| China |  |  |  |  |  |  |  |  |  |  |  |  |  |
| Malaysia | 3 | 3 |  | 1 | 2 | 3 |  |  |  |  |  | 1 | 4 |
| Indonesia | 4 |  |  | 3 | 3 | 5 |  |  |  |  |  |  |  |
| Singapore | 2 |  |  |  | 1 |  |  |  |  |  |  | 1 |  |
| Thailand | 4 |  |  |  |  |  |  |  |  |  |  |  |  |
| Iran | 2 | 1 | 1 | 1 |  | 1 | 6 |  | 1 |  |  |  | 7 |
| Ukraine | 2 |  |  |  |  | 1 |  |  | 1 |  |  | 1 |  |
| Jordan | 2 |  |  | 1 |  | 1 |  |  |  |  |  |  |  |
| Russia | 4 |  |  | 10 | 1 | 3 | 6 | 1 |  |  |  |  | 1 |
| Brazil | 2 |  |  | 2 | 2 |  |  |  |  |  |  | 1 | 2 |
| Argentina | 7 |  | 1 | 1 |  | 2 | 2 | 1 | 3 |  | 1 |  | 7 |
| Mexico | 2 |  |  |  |  |  |  |  | 1 |  |  | 1 |  |
| Peru |  |  |  |  |  |  |  |  |  |  |  |  |  |
| Cuba | 2 |  |  | 2 |  | 1 | 2 | 2 |  |  |  | 2 |  |
| Egypt | 2 |  |  |  |  |  |  |  |  |  |  |  |  |
| Ghana |  |  |  | 7 |  | 2 | 1 |  | 2 |  |  |  |  |
| Zambia |  |  |  | 1 |  | 3 |  |  |  |  |  |  |  |
| S. Africa | 1 |  |  |  |  |  |  |  |  |  |  |  |  |

| **mAbs** | **rituximab** | **adalimumab** | **bevacizumab** | **infliximab** | **trastuzumab** | **ranibizumab** | **denosumab** | **abciximab** | **nimotuzumab** | **omalizumab** | **eculizumab** | **etanercept** |
| --- | --- | --- | --- | --- | --- | --- | --- | --- | --- | --- | --- | --- |
| Target | CD20 | TNFα | VEGF | TNFα | HER2 | VEGF-A | RANKL | Glycoprotein  IIb/ IIIa  receptor | EGFR  domain III | High-affinity  receptor  binding site  of IgE | Terminal  complement  component 5 | TNF α/  TNFβ |
| EU | 6 | 10 | 5 | 4 | 6 |  |  |  |  |  |  | 3 |
| US | 3 | 6 | 2 | 4 | 5 |  |  |  |  |  |  | 2 |
| Canada | 2 | 1 | 1 | 5 | 2 |  |  |  |  |  |  | 2 |
| Australia | 2 | 2 | 1 | 2 | 2 |  |  |  |  |  |  | 2 |
| S. Korea | 1 | 1 |  | 2 | 2 |  |  |  |  |  |  | 3 |
| Japan | 2 | 1 | 2 | 3 | 4 |  |  |  |  |  |  | 2 |
| India | 7 | 4 | 6 | 1 | 5 | 2 | 1 | 1 | 1 | 1 |  | 4 |
| China | 2 | 4 | 2 | 1 | 2 |  |  |  |  |  |  | 3 |
| Malaysia | 1 | 1 | 2 | 1 | 3 |  |  |  |  |  |  |  |
| Indonesia | 2 |  |  |  | 3 |  |  |  |  |  |  |  |
| Singapore | 1 | 1 |  | 1 |  |  |  |  |  |  |  |  |
| Thailand | 3 | 1 | 1 |  | 4 |  |  |  |  |  |  |  |
| Iran | 2 | 1 | 1 |  | 1 |  |  |  |  |  |  | 1 |
| Ukraine | 1 |  |  |  | 1 |  |  |  |  |  |  |  |
| Jordan | 2 | 1 |  | 1 | 1 |  |  |  |  |  |  |  |
| Russia | 1 | 1 | 1 | 3 | 1 |  |  |  |  |  | 1 |  |
| Brazil | 2 | 1 | 1 | 3 | 4 |  |  |  |  |  |  | 1 |
| Argentina | 2 |  | 2 |  |  |  |  |  |  |  |  |  |
| Mexico | 1 |  |  | 1 |  |  |  |  |  |  |  |  |
| Peru | 2 |  |  | 1 | 1 |  |  | 1 |  |  |  |  |
| Cuba | 2 |  | 1 |  | 2 |  |  |  |  |  |  |  |
| Egypt | 1 |  |  |  | 1 |  |  |  |  |  |  |  |
| Ghana |  |  |  |  | 1 |  |  |  |  |  |  |  |
| Zambia |  |  | 2 |  | 2 |  |  |  |  |  |  |  |
| S. Africa |  |  |  |  | 1 |  |  |  |  |  |  |  |

**Table S4: Complete list of published literature on analytical similarity assessments available in public domain (Till Aug 2021)**

| **S. No** | **Attributes** | **Primary structure** | | **Higher order structure** | | | | **Glycosylation** | | | **Product-related variants** | | | **Process-related variants** | | | **References** |
| --- | --- | --- | --- | --- | --- | --- | --- | --- | --- | --- | --- | --- | --- | --- | --- | --- | --- |
|  | **Products** | **Intact/ subunit mass** | **Peptide map** | **Disulfide bridge/ free -SH** | **2° str.** | **3° str.** | **Conformational stability** | **Oligosaccharide pattern** | **Glycopeptide** | **Monosaccharide/ sialic acid** | **Aggregates/ fragments** | **Visible/ Sub-visible particles** | **Charge variants** | **Related proteins** | **HCP** | **HCD** |  |
| 1 | adalimumab | LC-ESI-TOF-MS | RP-UPLC-UV-MS^E^ | RP-UPLC-UV-MS^E^ | FTIR | Near UV CD | DSC | HILIC-FLD-MS with 2AA |  |  | SEC-UV, MALS, AUC-SV, FFF-LS, DLS, CE-SDS | MFI, LO | CEX, cIEF |  | ELISA,2D-LC-MS^E^, 2D-DIGE | qPCR | (Liu et al., 2016a) |
| 2 | adalimumab | RP-UV/ QTOF-MS | RP-UV/ QTOF-MS | RP-UV/ QTOF-MS | Far UV CD | Near UV CD | nanoDSC |  |  |  | SEC, gel-on-a-chip’ electrophoretic method with FLD |  |  |  |  |  | (Magnenat et al., 2017) |
| 3 | adalimumab |  |  |  |  |  |  |  |  |  | SEC, MALS, AUC-SV |  | CEX |  |  |  | (Shabestari et al., 2018) |
| 4 | adalimumab |  | RP-UPLC- MS^E^ | RP-UPLC- MS^E^ | Far UV CD, FTIR | Near UV CD, HDX-MS | NanoDSC | HILIC-UPLC-FLD with 2AB | RP-UPLC- MS^E^ with PNGase F + Asp-N | IEX-UV | SEC, CE-SDS |  | icIEF, CEX |  |  |  | (Lee et al., 2019b) |
| 5 | adalimumab |  |  |  | Far UV CD |  |  |  |  |  | SDS-PAGE |  |  |  |  |  | (Tabasinezhad et al., 2019) |
| 6 | adalimumab | LC-ESI-TOF-MS | Edman degradation, RP-ESI-Ion Trap MS |  | Far UV CD, FTIR | Near UV CD | DSC | RP-MALDI-TOF-MS |  | RP with fluorescein | SDS-PAGE, SEC, FFF, CE-SDS-LIF | MFI, LO | CEX, IEF |  | ELISA | threshold assay | (Schreiber et al., 2020) |
| 7 | adalimumab | LC-UV/ MS | LC-UV/ MS^E^ | LC-UV/ MS^E^, Free thiol FLD | Far UV CD | Near UV CD, FLR | DSC | HILIC-UPLC-FLD | LC-UV/ MS^E^ | HPLC-FLD | SEC, MALS, DLS, CE-SDS | MFI | CEX, icIEF | rCE-SDS | ELISA | qPCR | (Zhang et al., 2020) |
| 8 | adalimumab |  | RP-UV/ ESI-QTOF-MS |  |  |  |  |  |  |  | SEC |  | icIEF |  |  |  | (Derzi et al., 2020) |
| 9 | adalimumab | UPLC-QTOF-MS | UPLC-QTOF-MS | UPLC-QTOF MS | Far UV CD, FTIR | Near UV CD | DSC | HILIC-FLD/ QTOF-MS with 2AA |  |  |  |  |  |  |  |  | (Kwon et al., 2021) |
| 10 | agalsidase beta | MALDI-TOF-MS | RP-UV |  | Far UV CD |  |  | HILIC with pyridylamination by 2-aminopyridine |  | RP-FLD with DMB, Sialooligosaccharide purified using SEC |  |  | cIEF |  |  |  | (Morimoto et al., 2018) |
| 11 | bevacizumab |  |  |  |  |  |  |  |  |  | SEC |  | icIEF, CZE, CEX |  |  |  | (Zhao et al., 2016) |
| 12 | bevacizumab |  | RP-UV/ ESI-QTOF-MS |  |  |  |  |  |  |  |  |  |  |  |  |  | (Peraza et al., 2018) |
| 13 | bevacizumab | SEC-ESI-TOF-MS | RP-UV/ hybrid Ion Trap-Orbitrap MS | RP-UV/ hybrid Ion Trap-Orbitrap MS | FTIR | Near UV CD | DSC | HILIC-Ion Trap MS with 2AA |  |  | SEC, MALS, DLS, FFF, AUC-SV, CE-SDS | LO, MFI | cIEF, CEX |  |  |  | (Seo et al., 2018) |
| 14 | bevacizumab |  |  |  | FTIR | FLR |  |  |  |  | 90°LS | MFI, NTA, LM, NRM, URT |  |  |  |  | (Arvinte et al., 2019a) |
| 15 | bevacizumab |  |  |  |  | FLR | TCSPC |  |  |  | 90°LS, AF4 | MFI, NTA, NRM, URT |  |  |  |  | (Arvinte et al., 2019b) |
| 16 | bevacizumab | ESI-Native MS |  |  |  | ESI-IM-MS, nanoESI-time‐resolved HDX-MS |  |  |  |  |  |  |  |  |  |  | (Brown et al., 2019) |
| 17 | bevacizumab | RP-Q Exactive MS | RP-UV/ Q Exactive MS | RP-UV/ Q Exactive MS, Ellman assay | Far UV CD | Near UV CD | DSC | HILIC-FLD with 2AB |  |  | CE-SDS | LO, MFI | cIEF, CEX |  | ELISA | qPCR | (Yu et al., 2020) |
| 18 | bevacizumab |  |  |  |  |  |  |  |  |  | SEC |  | CEX, CZE, cIEF |  |  |  | (Prakash et al., 2020) |
| 19 | bevacizumab | ESI-TOF-MS | ESI-TOF- MS^E^ |  | Far UV CD, FTIR | FLR | TCSPC | HILIC-FLD |  |  | SDS-PAGE, SEC |  | CEX |  |  |  | (Singh et al., 2021) |
| 20 | darbepoetin-α | RP-QTOF-MS | UPLC-QTOF- UV/ MS | UPLC-QTOF- UV/MS | Far UV CD | Near UV CD, FLR |  | RP-MS, HILIC-FLD-QTOF-MS with PNGase F |  | LC-UV/ FLD | SDS-PAGE, SEC |  | CZE, IEF | RP-UV/ MS |  |  | (Jeong et al., 2018) |
| 21 | darbepoetin-α | MALDI-TOF-MS | Edman degradation, RP-UV/ MS |  | Far UV CD | Near UV CD |  | HILIC-FLD-MS with 2AB | RP-HPLC-UV/ MS with PNGase F | RP-FLD, HPAEC-PAD | SDS-PAGE |  | CZE |  |  |  | (Tani et al., 2020) |
| 22 | eculizumab | RP-ESI-TOF-MS | RP-UPLC-Q Exactive-Orbitrap MS | RP-UPLC-Q Exactive-Orbitrap MS | Far UV CD | Near UV CD | DSC | HILIC-Ion Trap MS |  |  | SEC-UV-MALS, SV-AUC, nrCE-SDS | LO, MFI | cIEF, AEX | HIC, rCE-SDS |  |  | (Hutterer et al., 2021) |
| 23 | epoietin-α |  |  |  | Far UV CD | Near UV CD, FLR | VT-CD |  |  | HPAEC-PAD | SEC, AUC-SV/SE |  | CZE |  |  |  | (Deechongkit et al., 2006) |
| 24 | epoietin-α |  |  |  |  | 9G8A antibody binding assay |  |  |  |  | SDS-PAGE |  | CZE, IEF |  |  |  | (Park et al., 2009) |
| 25 | epoietin-α |  |  |  |  |  |  |  |  |  | SDS-PAGE, SEC |  | CZE |  |  |  | (Brinks et al., 2011) |
| 26 | epoietin-α |  |  |  |  |  |  |  |  |  | SDS-PAGE, SEC-UV/ MALS, AF4 |  |  |  | ELISA |  | (Halim et al., 2014) |
| 27 | epoietin-α |  |  |  |  |  |  | LC/ FTICR linear and ion trap-MS |  |  |  |  |  |  |  |  | (Hashii et al., 2014) |
| 28 | epoietin-α |  |  |  |  |  |  |  |  |  | SDS-PAGE, SEC, MALS, AF4 |  | CZE |  |  |  | (Halim et al., 2016) |
| 29 | epoietin-α | UPLC-MS | UPLC-QTOF-MS |  |  |  |  | HILIC-UPLC-FLD/ MS |  |  |  |  |  |  |  |  | (Alley et al., 2020) |
| 30 | epoietin-α and darbepoetin-α | RP-ESI-MS with with α-sialidase |  |  |  |  |  |  |  |  |  |  |  |  |  |  | (Harazono et al., 2013) |
| 31 | etanercept | MALDI-TOF-MS | RP/ MALDI-TOF-MS |  |  |  |  | MALDI-TOF MS, HILIC-FLD with 2AB |  | resorcinol method | 2D-PAGE, SEC |  | 2D-SDS-PAGE | RP |  |  | (Maity et al., 2011) |
| 32 | etanercept | RP-ESI-QTOF-MS | RP-UV/ ESI-QTOF-MS |  |  |  |  | CE-LIF with APTS |  |  |  |  | CEX |  |  |  | (Tan et al., 2012) |
| 33 | etanercept | MALDI-TOF-MS | Edman degradation, RP/ MALDI-TOF-MS |  |  |  |  | HILIC-FLD, MALDI-TOF-MS with 2AB |  | HPAEC-PAD, Weak AEX | SDS-PAGE, SEC |  | IEF | BAC-FLD |  |  | (Lee et al., 2013) |
| 34 | etanercept |  | RP-UV/ QTOF-MS |  |  |  |  |  |  |  |  |  |  |  |  |  | (Huang et al., 2016) |
| 35 | etanercept | RP-QTOF-MS | RP-UPLC-QTOF-MS |  | Far UV CD |  | TCSPC | HILIC-FLD |  |  |  |  | cIEF, CEX |  |  |  | (Miranda-Hernández et al., 2016) |
| 36 | etanercept | RP-QTOF-MS |  |  |  | HDX-MS | DSC | HILIC-FLD / QTOF MS with procainamide |  | IEX-UV | SEC, CE-SDS |  |  | HIC |  |  | (Cho et al., 2016) |
| 37 | etanercept | MALDI-TOF-MS, UPLC-QTOF-MS | UPLC-QTOF-MS |  |  | UPLC-IM-MS |  | MALDI-TOF-MS with permethylated and β-elimination | UPLC-QTOF-MS |  |  |  |  |  |  |  | (Montacir et al., 2018b) |
| 38 | etanercept |  | RP-UV/ MS, MALDI-QTOF-MS |  |  |  |  | HILIC-FLD with 2AB |  | RP-FLD with DMB | SDS-PAGE, SEC |  | AEX, IEF | HIC-FLD | ELISA |  | (Hassett et al., 2018) |
| 39 | etanercept |  | nanoLC-ESI-Orbitrap Fusion Tribrid MS with HCD |  |  |  |  | MALDI-TOF-MS, NanoLC-ESI-Orbitrap Fusion Tribrid MS with CID |  |  |  |  |  |  |  |  | (Fazel et al., 2019) |
| 40 | filgrastim | MALDI-TOF/ RP-ESI-MS | Edman degradation, RP-UV/ MS |  | Far UV CD | Near UV CD, 1D-NMR |  |  |  |  | SDS-PAGE, SEC |  | CEX | RP |  |  | (Sörgel et al., 2010) |
| 41 | filgrastim | RP-ESI-QTOF-MS | MALDI-TOF-MS, LC-ESI/ QQQ-MS | MALDI-TOF-MS | Far UV CD | FLR |  |  |  |  | SDS-PAGE, SEC |  | CEX, IEF | RP |  |  | (Skrlin et al., 2010) |
| 42 | filgrastim |  |  |  |  |  |  |  |  |  | SDS-PAGE, SEC |  | cIEF | RP |  |  | (Shaltout et al., 2014) |
| 43 | filgrastim | MALDI-TOF-MS | Edman degradation, RP/ MALDI-TOF-MS | RP/ MALDI-TOF-MS |  |  |  |  |  |  | SDS-PAGE, SEC |  |  | RP | ELISA |  | (Crobu et al., 2014) |
| 44 | filgrastim | LC-Orbitrap MS with CID/ ETD | LC-Orbitrap MS with CID/ ETD/ HCD | LC-Orbitrap MS with CID/ ETD/ HCD | Far UV CD | 2D (1H, 15N-HSQC) NMR | VT-CD |  |  |  |  |  |  |  |  |  | (Levy et al., 2014) |
| 45 | filgrastim | UPLC-HDMS | UPLC-HDMS |  | Far UV CD | FLR |  |  |  |  | SDS-PAGE, SEC |  |  | RP | ELISA, 2D PAGE, UPLC-HDMS | Picogreen assay | (Rathore and Bhambure, 2014) |
| 46 | filgrastim | MALDI-TOF-MS, RP-Orbitrap MS | Edman degradation, RP-UV/ Orbitrap-MS |  | Far UV CD | 1D NMR |  |  |  |  | SDS-PAGE, SEC | MFI | CEX, IEF | RP | ELISA | Threshold assay | (Sörgel et al., 2015) |
| 47 | filgrastim | RP-UPLC-ESI-MS | RP-UPLC-ESI-MS |  | Far UV CD |  | DSC, TCSPC |  |  |  | SDS-PAGE, SEC |  | IEF | RP |  |  | (Mendoza-Macedo et al., 2016) |
| 48 | filgrastim | RP-ESI-TOF-MS | RP-ESI-TOF-MS |  | Far UV CD | RP-FLD |  |  |  |  | SDS-PAGE, SEC |  |  | RP |  |  | (Nupur et al., 2016) |
| 49 | filgrastim |  | MS^E^ |  | Far UV CD | Near UV CD, FLR, XRC | VT-CD |  |  |  | 2D-PAGE, SEC |  | 2D-PAGE | RP | ELISA | qPCR | (Magalhaes et al., 2016) |
| 50 | filgrastim | RP-UV/ FLD/ Exactive MS with HCD |  |  |  |  |  |  |  |  |  |  |  | RP-UV/ FLD/ MS |  |  | (Hausberger et al., 2016) |
| 51 | filgrastim |  | RP-UV |  | Far UV CD | FLR | VT-CD |  |  |  | SDS-PAGE, SEC | MFI |  | RP |  |  | (Halim et al., 2018) |
| 52 | filgrastim | RP-UV, MALDI-TOF-MS, GC-MS | RP-UV, MALDI-TOF-MS | MALDI-TOF-MS |  |  |  |  |  |  |  |  |  |  |  |  | (Gianoncelli et al., 2019) |
| 53 | filgrastim | LC-QTOF-MS | LC-QTOF-MS |  | Far-UV CD | Near UV CD, 2D (1H-15N-HSQC) NMR |  |  |  |  | SDS-PAGE, SEC |  |  | RP |  |  | (Bor Tekdemir et al., 2020) |
| 54 | infliximab | LC-ESI-QTOF-MS, RP-UPLC-Triple TOF MS | RP-UV/ FLD | Ellman assay | Far UV CD, FTIR | Near UV CD, Antibody conformational array, XRC | DSC | HILIC-FLD with 2AB | LC-ESI-QTOF-MS | HPAEC-PAD | SEC, MALS, SV-AUC, CE-SDS |  | CEX, IEF |  |  |  | (Jung et al., 2014) |
| 55 | infliximab |  | RP-UPLC-QTOF-MS |  |  | HDX-MS, IM-MS |  | HILIC-UPLC-FLD-QTOF-MS with RapiFluor-MS |  |  |  |  |  |  | 2D LC (RPXRP)-QTOF-MS |  | (Fang et al., 2016) |
| 56 | infliximab | RP-UPLC-ESI-QTOF-MS | RP- UHR-UV/ ESI-QTOF-MS |  |  |  |  |  |  |  | SEC |  | icIEF |  |  |  | (Derzi et al., 2016) |
| 57 | infliximab | RP-ESI-HDMS |  |  | Far UV CD | Near UV CD |  |  |  |  |  |  | cIEF |  |  |  | (Velasco-Velázquez et al., 2017) |
| 58 | infliximab |  |  |  |  |  |  | HILIC-UPLC-FLD with 2AB |  |  |  |  |  |  |  |  | (Lee et al., 2017) |
| 59 | infliximab | LC-ESI-MS | LC-ESI- MS^E^ | LC-ESI- MS^E^ | Far UV CD | Near UV CD, FLR, HDX-MS, Antibody conformational array | DSC | HILIC-UPLC-FLD with 2AB, LC-ESI- MS^E^ with procainamide | LC-ESI- MS^E^ |  | SEC, MALS, SV-AUC, CE-SDS |  | CEX, icIEF |  |  |  | (Hong et al., 2017) |
| 60 | infliximab |  | RP-QTOF-MS |  |  |  |  |  |  |  |  |  | CEX, 2D-DIGE |  |  |  | (Beyer et al., 2019) |
| 61 | infliximab | RP-QTOF-MS | RP-UV/ ESI-MS^E^ |  | Far UV CD | Near UV CD |  | HILIC-FLD/ MS^E^ with 2AB |  |  | SDS-PAGE, SEC |  |  |  |  |  | (An et al., 2019) |
| 62 | infliximab | SEC-UPLC‑UV/ HESI-[Native] MS |  |  | Far UV CD | FLR | VT-CD |  |  |  | SEC-UPLC‑UV/ HESI-[Native] MS, DLS |  |  |  |  |  | (Hermosilla et al., 2019) |
| 63 | infliximab |  | RP-UV |  |  |  |  |  |  |  | SEC, DLS, Quantitative gel electrophoresis using TapeStation |  | CEX | RP |  |  | (Hassan et al., 2019) |
| 64 | infliximab | LC-ESI-MS | LC-UV/ ESI-MS, Edman degradation | LC-ESI-MS | Far UV CD, FTIR | Near UV CD, FLR, XRC |  | HILIC-FLD/ MS | ESI-QTOF-MS |  | SEC, CE-SDS, AUC-SE | LO HIAC | CEX, iCE | CE-SDS |  |  |  |
| 65 | infliximab |  |  |  |  | XRC |  | HILIC-FLD with 2AB |  |  | AUC-SV |  |  |  |  |  | (Lerch et al., 2020) |
| 66 | infliximab | SEC-ESI-TOF-MS | UPLC-ESI-Hybrid MS | UPLC-ESI-Hybrid MS | FTIR | Near UV CD | DSC | HILIC-FLD/ Ion trap-MS with 2AA |  |  | SEC, MALS, SV-AUC, FFF, DLS, CE-SDS | MFI, LO | CEX, cIEF | UPLC-ESI-MS |  |  | (Saleem et al., 2020) |
| 67 | infliximab, trastuzumab and bevacizumab |  |  |  |  |  |  | HILIC-ESI-QTOF-MS |  |  |  |  |  |  |  |  | (Planinc et al., 2017b) |
| 68 | insulin glargine | ESI-QTOF-MS | nanoUPLC-MS |  | Far UV CD |  |  |  |  |  | SDS-PAGE |  |  | RP |  |  | (Escobedo-Moratilla et al., 2016) |
| 69 | insulin glargine | RP-ESI-MS | RP-ESI-MS^E^ | RP-ESI-MS^E^ | Far UV CD, FTIR | Near UV CD, FLR, 1D NMR, 2D (1H, 1H-TOCSY) NMR, 2D (1H, 1H-NOESY), XRC | DSC |  |  |  | SEC, AUC-SV |  |  | RP |  |  | (Goyal et al., 2021) |
| 70 | interferon beta |  |  |  |  |  |  |  |  |  | SDS-PAGE |  | SDS-PAGE | Non denaturing RP |  |  | (Meager et al., 2011) |
| 71 | peg-filgrastim |  | RP-UPLC-UV/ ESI-MS |  | Far UV CD | HDX-MS |  |  |  |  | SDS-PAGE, SEC |  | CEX | RP |  |  | (Brokx et al., 2017) |
| 72 | peg-filgrastim | LC-MS | Edman degradation, LC-Triple TOF-MS |  | Far UV CD, FTIR | FLR, 1D NMR, 2D (1H-13C-HSQC) NMR | DSC |  |  |  | SDS-PAGE, SEC |  | CEX | RP, LC-MS |  |  | (Shekhawat et al., 2019) |
| 73 | ranibizumab and aflibercept |  |  |  | Far UV CD | FLR | DSC |  |  |  | SDS-PAGE, SEC | NTA |  |  |  |  | (Moreno et al., 2016) |
| 74 | rFVIIa | SELDI-MS |  |  |  |  |  |  |  |  | SDS-PAGE |  |  |  |  |  | (Sadeghi et al., 2014) |
| 75 | rFVIIa | MALDI-TOF-MS | nanoLC-ESI- MS^E^ with CID |  | Far UV CD, FTIR | UPLC-IM-MS, 1D NMR |  | MALDI-TOF-MS with permethylation | RP-UPLC-QTOF-MS |  |  |  |  |  |  |  | (Montacir et al., 2018a) |
| 76 | rhCG | LC-ESI-Triple TOF-MS | LC-ESI-MS |  | Far UV CD | UV spectroscopy, FLR |  | HILIC-ESI-MS with 2AB | LC-ESI-MS |  | SDS-PAGE, SEC |  | cIEF, CZE | RP |  |  | (Thennati et al., 2018) |
| 77 | r-hFSH | MALDI-TOF-MS | LC-ESI-MS |  |  | UV spectroscopy |  |  |  |  | SDS-PAGE, SEC |  | IEF | SDS-PAGE |  |  | (Winstel et al., 2017) |
| 78 | r-hFSH |  |  |  |  |  |  | HILIC-MS with CID frag |  |  |  |  |  |  |  |  | (Mastrangeli et al., 2017) |
| 79 | rituximab |  | LC-nanospray ion source-Orbitrap-MS with CID and ETD | LC-nanospray ion source-Orbitrap-MS with CID and ETD |  |  |  |  | LC-nanospray ion source-Orbitrap-MS with CID and ETD |  |  |  |  |  |  |  | (Li et al., 2013) |
| 80 | rituximab | RP-UV/ ESI-MS | RP-UV/ ESI-QTOF-MS | Ellman’s Assay, RP-UV/ ESI-QTOF-MS |  | Near UV CD, XRC, HDX-MS, 1D NMR | DSC | HILIC-FLD with 2AB |  |  |  |  |  | BAC-FLD |  |  | (Visser et al., 2013) |
| 81 | rituximab |  |  |  |  |  |  | HILIC-FLD with 2AB |  |  | SEC, AF4, CE-SDS |  | CEX |  |  |  | (Da Silva et al., 2014) |
| 82 | rituximab | RP-ESI-MS | RP-UV |  |  |  |  |  |  |  | SEC, MALS, CE-SDS |  | CEX | HIC |  |  | (Flores-Ortiz et al., 2014) |
| 83 | rituximab |  | RP-UV-QTOF-MS |  |  |  |  |  |  |  |  |  |  |  |  |  | (Ryan et al., 2014) |
| 84 | rituximab | UPLC-ESI-MS |  |  |  |  | ITC | HILIC-FLD |  |  | SEC |  | cIEF |  |  |  | (Miranda-Hernandez et al., 2015) |
| 85 | rituximab | UPLC-QTOF-MS | nanoLC-ESI- MS^E^ with CID |  | Far UV CD, FTIR | UPLC-IM-MS, 1D NMR |  | MALDI-TOF-MS | UPLC-QTOF-MS, MALDI-TOF-MS |  |  |  |  |  |  |  | (Montacir et al., 2017) |
| 86 | rituximab | LC-QTOF-MS | LC-UV/ Triple TOF-MS | LC-UV/ Triple TOF-MS, Ellman assay | Far UV CD, FTIR | Near UV CD | DSC | HILIC-FLD with 2AB | LC-ESI-MS | HPAEC-PAD, RP | SEC, MALS, SV-AUC, CE-SDS |  | CEX, IEF | LC-ESI-MS |  |  | (Lee et al., 2018b) |
| 87 | rituximab | RP-ESI-TOF-MS | RP-ESI-TOF-MS |  | Far UV CD, FTIR | Near UV CD, FLR |  | HILIC-QTOF-MS with procainamide |  |  | SDS-PAGE, SEC, DLS |  | CEX |  |  |  | (Nupur et al., 2018) |
| 88 | rituximab | RP/ SEC-Triple TOF-MS | RP-UV/ ESI-Triple TOF-MS | LC-ESI-Triple TOF-MS, Ellman Assay | Far UV CD, FTIR | Near UV CD, FLR, NMR | DSC | HILIC-ESI-Triple TOF-MS with 2AA | LC–ESI-MS |  | SDS-PAGE, SEC, MALS, CE-SDS |  | cIEF, CEX, CZE | RP, BAC |  |  | (Singh et al., 2018) |
| 89 | rituximab | LC-ESI-MS | LC-ESI-QTOF-MS |  | Far UV CD | FLR | DSC, TCSPC | HILIC-FLD with 2AB |  | HPAEC-PAD | SEC, SV-AUC, SLS |  | CEX |  |  |  | (Cerutti et al., 2019) |
| 90 | rituximab | UPLC-QTOF-MS | RP-UV/ ESI-MS, UPLC-QTOF-MS | UPLC-QTOF-MS, Measure-iT thiol assay |  | Near UV CD | NanoDSC | HILIC-FLD with RapiFluor-MS |  | RP-FLD with DMB | SEC, CE-SDS |  | icIEF, CEX |  | ELISA | qPCR | (Xu et al., 2019) |
| 91 | rituximab | LC-QTOF-MS | nanoUPLC- MS |  |  | QIM-TOF-MS, HDX-MS | CIU with IM-MS | HILIC-FLD with RapiFluor-MS |  |  | SEC |  | CEX |  |  |  | (Kang et al., 2020b) |
| 92 | rituximab | ESI-TOF-MS | LC-hybrid ion trap-Orbitrap MS | LC-hybrid ion trap-Orbitrap MS | FTIR | Near UV CD | DSC | HILIC-FLD/ ion-trap MS with 2AA |  |  | SEC, MALS, SV-AUC, FFF, DLS, CE-SDS | LO, MFI | cIEF, CEX |  |  |  | (Seo et al., 2020) |
| 93 | rituximab |  |  |  |  |  |  |  |  |  | SEC, DLS, gel electrophoresis |  |  | RP |  |  | (Trabik et al., 2020) |
| 94 | rituximab | RP-UPLC- MS^E^ | RP-UPLC- MS^E^ | RP-UPLC- MS^E^ |  |  |  | HILIC-FLD/ QTOF-MS, CE-LIF |  | RP-UPLC with DMB | SEC, CE-SDS |  | cIEF, icIEF, CEX |  |  |  | (Wang et al., 2021) |
| 95 | rituximab |  |  |  |  |  |  | MALDI-TOF-MS, UPLC-FLD with 2AB |  | UPLC-FLD with DMB | SDS-PAGE |  |  |  |  |  | (Kaur et al., 2021) |
| 96 | rituximab, bevacizumab, and trastuzumab | RP-Q Exactive-MS |  |  | Far UV CD | FLR, QIM-TOF-MS | CIU with IM-MS |  |  |  | SDS-PAGE, SEC |  | CEX |  |  |  | (Kang et al., 2020a) |
| 97 | teriparatide | LC-ESI-HRMS | LC- MS^E^ |  | Far UV CD | 2D (1H-15N-HMQC) NMR |  |  |  |  | SDS-PAGE, SEC |  | IEX | RP |  |  | (Kovács et al., 2020) |
| 98 | tocilizumab | RP-UPLC-QTOF-MS | RP-UPLC-QTOF-MS | Ellman Assay | Far UV CD | Near UV CD | DSC | HILIC-FLD with 2AB |  |  | SEC, MALS, CE-SDS |  | icIEF, CEX |  |  |  | (Miao et al., 2017) |
| 99 | tocilizumab |  |  |  |  | FLR |  |  |  |  | SEC, DLS |  | CEX |  |  |  | (Navas et al., 2020) |
| 100 | trastuzumab | RP-ESI-MS | RP-ESI-MS |  |  |  |  | MALDI-TOF-MS, HILIC-FLD with 2AB |  |  |  |  |  |  |  |  | (Xie et al., 2010) |
| 101 | trastuzumab |  | nanoLC-Ion Trap-Orbitrap with CID and ETD |  |  |  |  |  |  |  |  |  |  |  |  |  | (Chen et al., 2013) |
| 102 | trastuzumab |  | RP-UHD-QTOF-MS |  |  |  |  |  |  |  |  |  |  |  |  |  | (Hurst et al., 2014) |
| 103 | trastuzumab | RP-UPLC-QTOF-MS | RP-UPLC-QTOF-MS |  | Far UV CD | Near UV CD | DSC, TCSPC | HILIC-FLD with 2AB, CE-LIF with APTS |  |  | SEC, nrCE-SDS |  | CEX, cIEF | rCE-SDS |  |  | (López-Morales et al., 2015) |
| 104 | trastuzumab |  |  |  |  |  | ITC | HILIC-UPLC-FLD |  |  | SEC |  | CEX, cIEF |  |  |  | (Miranda-Hernández et al., 2015) |
| 105 | trastuzumab | RP-UPLC-QTOF-MS with lockspray ion source | RP-UPLC-QTOF-MS with lockspray ion source |  |  |  | DSC |  |  |  | SEC, CE-SDS |  |  | RP |  |  | (Chen et al., 2016) |
| 106 | trastuzumab |  |  |  |  |  |  | HILIC-FLD-MS with 2AB |  |  | SEC, CE-SDS |  | icIEF |  |  |  | (Kim et al., 2017) |
| 107 | trastuzumab |  | RP-UPLC-UV/ ESI-Triple TOF-MS | Ellman assay, RP-UPLC-ESI-Triple TOF-MS | Far UV CD, FTIR | Near UV CD | DSC | HILIC-FLD with 2AB | LC-MS | acid hydrolysis+RP-FLD | SEC, MALS, AUC-SV, nrCE-SDS |  | CEX, IEF | LC-ESI-QTOF-MS, rCE-SDS |  |  | (Lee et al., 2018a) |
| 108 | trastuzumab |  |  |  | Far UV CD | Near UV CD, FLR | VT-CD |  |  |  |  |  |  |  |  |  | (Pérez et al., 2019) |
| 109 | trastuzumab |  |  |  |  |  |  | HILIC-UPLC-FLD with 2AB |  |  |  |  |  |  |  |  | (Lee et al., 2019a) |
| 110 | trastuzumab | RP-ESI-TOF-MS | RP-UPLC-UV/ MS |  | FTIR | Near UV CD | DSC | HILIC-UPLC-FLD with 2AB |  |  | SEC, MALS, SV‑AUC | LO, MFI |  |  |  |  | (Hutterer et al., 2019) |
| 111 | trastuzumab | RP-UPLC-QTOF-MS | RP-UPLC-QTOF-MS, acid hydrolysis method+RP-FLD | RP-UPLC-QTOF-MS, Measure-iT thiol assay | Far UV CD, FTIR | FLR, 1D NMR | DSC | HILIC-UPLC-FLD with RapiFluor-MS | RP-UPLC-QTOF-MS | RP-HPLC with DMB, acid hydrolysis+AEX | SEC, MALS, SV-AUC, DLS, CE-SDS | MFI | CEX, cIEF |  | ELISA | qPCR | (Xie et al., 2020) |
| 112 | trastuzumab |  |  |  |  | FLR, UV spectroscopy |  |  |  |  | SEC, DLS | Turbidimetry at 350nm | CEX |  |  |  | (Guyader et al., 2020) |
| 113 | trastuzumab | ESI-TOF-MS | ESI-TOF-MS |  | Far UV CD, FTIR | Near UV CD, FLR |  |  |  |  | SDS-PAGE, SEC, DLS |  | CEX |  |  |  | (Joshi and Rathore, 2020) |
| 114 | trastuzumab & rituximab |  |  |  |  | FLR | NanoDSF, DSC, VT-CD |  |  |  |  |  |  |  |  |  | (Joshi et al., 2020) |
| 115 | ziv-aflibercept |  |  |  | Far UV CD | FLR |  |  |  |  | SEC, THT binding assay, Congo red binding assay, TEM, DLS |  |  |  |  |  | (Hermosilla et al., 2020) |
| 116 | ziv-aflibercept |  | Q-Exactive MS |  |  |  |  | HILIC-FLD with RapiFluor MS | Q-Exactive MS | RP-FLD with DMB |  |  |  |  |  |  | (Shen et al., 2021) |

**Table S5: Patent expiration of various rDNA-based products (mAbs/ non mAbs) in European Union (EU) and United States (US)**

| **MABS** | **Innovator (Manufacturer, Approval year)** | **EMA** | **USFDA** |
| --- | --- | --- | --- |
| rituximab | Rituxan (Roche, 1997) | 2013 | 2018 |
| adalimumab | Humira (Abbvie, 2002) | 2017 | 2023 |
| bevacizumab | Avastin (Roche, 2004) | 2022 | 2019 |
| infliximab | Remicade (Janssen, 1998) | 2015 | 2018 |
| trastuzumab | Herceptin (Roche, 1998) | 2014 | 2019 |
| cetuximab | Erbitux (Eli Lilly, 2004) | 2014 | 2016 |
| ranibizumab | Lucentis (Genentech, 2006) | 2022 | 2020 |
| denosumab | Prolia (Amgen, 2010) | 2022 | 2025 |
| abciximab | ReoPro (Janssen, 1993) | NA | 2015 |
| omalizumab | Xoliar (Genentech, 2003) | 2017 | 2017 |
| tocilizumab | Actemra (Genentech, 2009) | 2017 | 2015 |
| etanercept | Enbrel (Amgen, 1998) | 2015 | 2028 |
| ziv-aflibercept | Eylea (Regeneron, 2011) | 2027 | 2027 |
| **NON MABS** |  |  |  |
| filgrastim | Neupogen (Amgen, 1991) | 2006 | 2013 |
| pegfilgrastim | Neulasta (Amgen, 2002) | 2017 | 2015 |
| teriparatide | Forteo (Eli Lilly, 2002) | 2019 | 2019 |
| human insulin | Humulin (Eli Lilly, 1992) | NA | 2000 |
| insulin glargine | Lantus (Sanofi, 2000) | 2014 | 2014 |
| insulin lispro | Humalog (Eli Lilly, 1996) | 2013 | 2013 |
| insulin aspart | Novolog (Novo Nordisk, 2000) | 2011 | 2014 |
| epoetin alfa | Epogen (Amgen, 1989) | 2004 | 2013 |
| darbepoetin alfa | Aranesp (Amgen, 2001) | 2016 | 2024 |
| interferon alfa-2b | Interon A (Biogen, 1986) | 2002 | 2011 |
| peg-interferon alfa-2b | Pegintron (Merck, 2000) | 2018 | 2018 |
| interferon beta-1a | Avonex (Biogen, 2003) | 2015 | 2015 |
| follitropin alfa | Gonal-f (Merck, 1995) | 2009 | 2019 |
| r-hCG | Ovidrel (Merck, 2000) | NA | 2029 |
| r-FVIIa | Novoseven (Novo Nordisk, 1996) | 2009 | 2010 |
| somatropin | Humatrope (Eli Lilly, 1986) | NA | 1999 |
| agalsidase beta | Fabrazyme (Sanofi, 2001) | 2013 | 2015 |
| enoxaparin sodium | Lovenox (Sanofi, 1993) | 2012 | 2004 |

**Table S6: CQA-wise summary of orthogonal platforms in published literature for analytical similarity assessment**

| **Attributes** | **Analysis** | **Criticality** | **Analytical tools** | **Most common tool** | **Publications (with orthogonal tools)** |
| --- | --- | --- | --- | --- | --- |
| **Primary structure/ identity** | Intact mass | CQA | RP-UV/ ESI-MS (CID^1^/ ETD^2^/ HCD^3^)^α^  MALDI-TOF-MS^β^  LC-HESI-[Native] MS^γ^ | RP-ESI-MS | (Sörgel et al., 2010)^α, β^, (Maity et al., 2011)^β^, (Lee et al., 2013)^β^, (Crobu et al., 2014)^β^, (Levy et al., 2014)^2α^, (Sörgel et al., 2015)^α, β^, (Hausberger et al., 2016)^3α^, (Winstel et al., 2017)^β^, (Morimoto et al., 2018)^β^, (Montacir et al., 2018a)^β^, (Singh et al., 2018)^α, β^, (Seo et al., 2018)^γ^, (Montacir et al., 2018b)^β^, (Gianoncelli et al., 2019)^β^, (Brown et al., 2019)^γ^, (Hermosilla et al., 2019)^γ^, (Tani et al., 2020)^β^, (Saleem et al., 2020)^γ^ |
|  | Peptide mapping/ Amino acid sequence | CQA | Reduced RP-ESI-MS (CID^1^/ ETD^2^/ HCD^3^)^α^  Reduced RP-UV^β^  MALDI-TOF-MS^γ^  Edman degradation^δ^ | Reduced RP-ESI-MS | (Sörgel et al., 2010)^α, β, δ^, (Skrlin et al., 2010)^α, γ^, (Maity et al., 2011)^γ^, (Tan et al., 2012)^α, β^, (Lee et al., 2013)^γ, δ^, (Visser et al., 2013)^α, β^, (Crobu et al., 2014)^γ, δ^, (Ryan et al., 2014)^α, β^, (Sörgel et al., 2015)^α, β^, (Huang et al., 2016)^α, β^, (Derzi et al., 2016)^α, β^, (Liu et al., 2016a)^α, β^, (Brokx et al., 2017)^α, β^, (Magnenat et al., 2017)^α, β^, (Jeong et al., 2018)^α, β^, (Lee et al., 2018b)^α, β^, (Singh et al., 2018)^α, β^, (Peraza et al., 2018)^α, β^, (Seo et al., 2018)^α, β^, (Hassett et al., 2018)^α, β, γ^, (Lee et al., 2018a)^α, β^, (Gianoncelli et al., 2019)^α, β, γ^, (Shekhawat et al., 2019)^α, δ^, (Xu et al., 2019)^α, β^, (Fazel et al., 2019)^3α^, (An et al., 2019)^α, β^, (McClellan et al., 2019)^α, β, δ^, (Hutterer et al., 2019)^α, β^, (Tani et al., 2020)^α, β, δ^, (Yu et al., 2020)^α, β^, (Schreiber et al., 2020)^α, δ^, (Derzi et al., 2020)^α, β^ |
| **Higher order structure** | Disulfide bridging | CQA | Non reduced RP-ESI-MS (CID^1^/ ETD^2^/ HCD^3^)^α^  Non reduced RP-UV^β^  MALDI-TOF-MS^γ^ | Non reduced RP-ESI-MS | (Skrlin et al., 2010)^γ^, (Li et al., 2013)^2α^, (Visser et al., 2013)^α, β^, (Crobu et al., 2014)^γ^, (Levy et al., 2014)^3α^, (Liu et al., 2016a)^α, β^, (Magnenat et al., 2017)^α, β^, (Jeong et al., 2018)^α, β^, (Lee et al., 2018b)^α, β^, (Seo et al., 2018)^α, β^, (Gianoncelli et al., 2019)^γ^, (Yu et al., 2020)^α, β^, (Zhang et al., 2020)^α, β^ |
|  | Secondary structure | CQA | Far UV CD^α^  FTIR^β^ | Far UV CD | (Jung et al., 2014)^α, β^, (Montacir et al., 2017)^α, β^, (Montacir et al., 2018a)^α, β^, (Lee et al., 2018a)^α, β^, (Lee et al., 2018b)^α, β^, (Nupur et al., 2018)^α, β^, (Singh et al., 2018)^α, β^, (Shekhawat et al., 2019)^α, β^, (McClellan et al., 2019)^α, β^, (Lee et al., 2019b)^α, β^, (Schreiber et al., 2020)^α, β^, (Xie et al., 2020)^α, β^, (Joshi and Rathore, 2020)^α, β^, (Goyal et al., 2021)^α, β^, (Singh et al., 2021)^α, β^, (Kwon et al., 2021)^α, β^ |
|  | Tertiary structure | CQA | Near UV CD^α^  FLR^β^  1D NMR^γ^  HDX-MS^δ^  IM-MS^ε^  XRC^ζ^  2D NMR^η^  Antibody conformational array^κ^  UV spectroscopy^μ^ | Near UV CD | (Deechongkit et al., 2006)^α, β^, (Sörgel et al., 2010)^α, γ^, (Visser et al., 2013)^α, γ, δ, ζ^, (Levy et al., 2014)^η^, (Jung et al., 2014)^α, ζ, κ^, (Sörgel et al., 2015)^γ^, (Magalhaes et al., 2016)^α, β, ζ^, (Cho et al., 2016)^δ^, (Fang et al., 2016)^δ, ε^, (Winstel et al., 2017)^μ^, (Brokx et al., 2017)^δ^, (Montacir et al., 2017)^γ, ε^, (Hong et al., 2017)^α, β, δ, κ^, (Jeong et al., 2018)^α, β^, (Thennati et al., 2018)^β, μ^, (Montacir et al., 2018a)^γ, ε^, (Nupur et al., 2018)^α, β^, (Singh et al., 2018)^α, β, γ^, (Montacir et al., 2018b)^ε^, (Shekhawat et al., 2019)^β, γ, η^, (Brown et al., 2019)^δ, ε^, (McClellan et al., 2019)^α, β, ζ^, (Lee et al., 2019b)^α, δ^, (Pérez et al., 2019)^α, β^, (Bor Tekdemir et al., 2020)^α, η^, (Kovács et al., 2020)^η^, (Kang et al., 2020b)^δ, ε^, (Lerch et al., 2020)^ζ^, (Zhang et al., 2020)^α, β^, (Kang et al., 2020a)^β, ε^ , (Xie et al., 2020)^β, γ^, (Guyader et al., 2020)^β, μ^, (Joshi and Rathore, 2020)^α, β^, (Goyal et al., 2021)^α, β, η^ |
|  | Conformational stability | CQA | DSC/ NanoDSC^α^  TCSPC^β^  VT-CD^γ^  ITC^δ^  IM-MS (CIU)^ε^  NanoDSF^ζ^ | DSC | (Levy et al., 2014)^γ^, (Magalhaes et al., 2016)^γ^, (Halim et al., 2018)^γ^, (Hermosilla et al., 2019)^γ^, (Pérez et al., 2019), (Cerutti et al., 2019)^α, β^, (Arvinte et al., 2019a)^β^, (Arvinte et al., 2019b)^β^, (Kang et al., 2020a)^ε^, (Kang et al., 2020b)^ε^, (Joshi et al., 2020)^α, γ, ζ^, (Singh et al., 2021)^β^ |
| **Glycosylation** | Oligosaccharide pattern/ monosaccharide/ sialic acid content | CQA | NP-HILIC-FLD (2AA^1^/ 2AB^2^/ procainamide^3^)^α^  NP-HILIC-MS (2AA^1^/ 2AB^2^/ procainamide^3^/ RapiFluor^4^)^β^  MALDI-TOF-MS (permethylation and beta elimination)^γ^  CZE-LIF (APTS)^δ^  RP-FLD (DMB)^ε^  HPAEC-PAD^ζ^  NP-WAX-FLD^η^ | NP-HILIC-FLD (2AB) | (Xie et al., 2010)^2α, γ^, (Maity et al., 2011)^2α, γ^, (Tan et al., 2012)^δ^, (Lee et al., 2013)^2α, γ, ζ, η^, (Jung et al., 2014)^2α, ζ^, (López-Morales et al., 2015)^2α, δ^, (Cho et al., 2016)^3α, β, η^, (Fang et al., 2016) ^α, 4β^, (Liu et al., 2016a)^1α, β^, (Mastrangeli et al., 2017)^β^, (Hong et al., 2017)^2α, 3β^, (Kim et al., 2017)^2α, β^, (Planinc et al., 2017b)^β^, (Montacir et al., 2017)^γ^, (Jeong et al., 2018)^α, β^, (Thennati et al., 2018)^2α, β^, (Morimoto et al., 2018)^α, ε^ , (Hassett et al., 2018)^2α, ε^ , (Lee et al., 2018a)^2α, ε^ , (Lee et al., 2018b)^2α, ε, ζ^, (Nupur et al., 2018)^3β^, (Singh et al., 2018)^1α, β^, (Seo et al., 2018)^1α, β^, (Montacir et al., 2018a)^γ^, (Montacir et al., 2018b)^γ^, (An et al., 2019)^2α, β^, (McClellan et al., 2019)^α, β^, (Xu et al., 2019)^α, 4β, ε^, (Fazel et al., 2019)^γ^, (Cerutti et al., 2019)^2α, ζ^, (Lee et al., 2019b)^2α, η^, (Alley et al., 2020)^α, β^, (Tani et al., 2020)^2α, β, ε, ζ^, (Seo et al., 2020)^1α, β^, (Saleem et al., 2020)^1α, β^, (Kang et al., 2020b)^α, 4β^, (Xie et al., 2020)^α, 4β, ε , η^, (Schreiber et al., 2020)^γ, ε^, (Zhang et al., 2020)^α, ε^, (Wang et al., 2021)^α, β, δ, ε^, (Kwon et al., 2021)^1α, β^, (Shen et al., 2021)^α, 4β, ε^, (Kaur et al., 2021)^2α, γ, ε^, (Hutterer et al., 2021)^α, β^ |
| **Product-related variant/ Purity** | Aggregates/ fragments (sub-visible and visible particles) | CQA | SEC-UV^α^  Gel electrophoresis/ SDS-PAGE^β^  SEC-MALS^γ^  Non reduced CGE^δ^  SV/ SE-AUC^ε^  SLS/ DLS^ζ^  MFI^η^  FFF/ AF4^κ^  HIAC LO^μ^  NTA^ν^  Optical microscopy^ρ^  TEM^τ^ | SEC-UV | (Halim et al., 2014)^α, γ, κ^, (Da Silva et al., 2014)^α, δ, κ^, (Flores-Ortiz et al., 2014)^α, γ, δ^, (Jung et al., 2014)^α, γ, δ, ε^, (López-Morales et al., 2015)^α, δ^, (Sörgel et al., 2015)^α, η^, (Halim et al., 2016)^α, γ, κ^, (Cho et al., 2016)^α, δ^, (Liu et al., 2016a)^α, γ, δ, ε, ζ, η, κ, μ^, (Chen et al., 2016)^α, δ^, (Moreno et al., 2016)^α, ν^, (Hong et al., 2017)^α, γ, δ, ε^, (Kim et al., 2017)^α, δ^, (Miao et al., 2017)^α, γ, δ^, (Lee et al., 2018a)^α, γ, δ, ε^, (Lee et al., 2018b)^α, γ, δ, ε^, (Nupur et al., 2018)^α, ζ^, (Singh et al., 2018)^α, γ, δ^, (Halim et al., 2018)^α, η^, (Seo et al., 2018)^α, γ, δ, ε, ζ, η, κ, μ^, (Shabestari et al., 2018)^α, γ, ε^, (Xu et al., 2019)^α, δ^, (Arvinte et al., 2019a)^ζ, η, ν, ρ^, (Arvinte et al., 2019b)^ζ, η, κ, ν, ρ^, (McClellan et al., 2019)^α, δ, ε, μ, ν^, (Hutterer et al., 2019)^α, γ, ε, η, μ^, (Lee et al., 2019b)^α, δ^, (Cerutti et al., 2019)^α, ε, ζ^, (Hermosilla et al., 2019)^α, ζ^, (Hassan et al., 2019)^α, ζ^, (Seo et al., 2020)^α, γ, δ, ε, ζ, η, κ, μ^, (Yu et al., 2020)^α, δ, η, μ^, (Trabik et al., 2020)^α, ζ^, (Saleem et al., 2020)^α, γ, δ, ε, ζ, η, κ, μ^, (Schreiber et al., 2020)^α, δ, η, κ, μ^, (Zhang et al., 2020)^α, γ, δ, ζ, η^, (Xie et al., 2020)^α, γ, δ, ε, ζ, η^, (Guyader et al., 2020)^α, ζ^, (Joshi and Rathore, 2020)^α, ζ^, (Navas et al., 2020)^α, ζ^, (Hermosilla et al., 2020)^α, ζ, τ^, (Wang et al., 2021)^α, δ^, (Goyal et al., 2021)^α, ε^, (Hutterer et al., 2021)^α, γ, ε, η, μ^ |
|  | Charge variant | kQA | IEX-UV (CEX/ AEX)^α^  IEF^β^  cIEF^γ^  icIEF^δ^  CZE^ε^  2D-PAGE/ DIGE^ζ^ | CEX-UV | (López-Morales et al., 2015)^α, γ^, (Miranda-Hernández et al., 2015)^α, γ^, (Miranda-Hernández et al., 2016)^α, γ^, (Liu et al., 2016a)^α, γ^, (Zhao et al., 2016)^α, δ, ε^, (Hong et al., 2017)^α, δ^, (Miao et al., 2017)^α, δ^, (Singh et al., 2018)^α, γ, ε^, (Seo et al., 2018)^α, γ^, (Thennati et al., 2018)^α, ε^, (Xu et al., 2019)^α, δ^, (Lee et al., 2019b)^α, δ^, (McClellan et al., 2019)^α, δ^, (Beyer et al., 2019)^α, ζ^, (Seo et al., 2020)^α, γ^, (Yu et al., 2020)^α, γ^, (Prakash et al., 2020)^α, γ, ε^, (Saleem et al., 2020)^α, γ^, (Xie et al., 2020)^α, γ^, (Zhang et al., 2020)^α, δ^, (Wang et al., 2021)^α, γ, δ^, (Hutterer et al., 2021)^α, γ^ |
|  | Related protein | CQA | RP-UV^α^/ ESI-MS^β^  Reduced CGE^γ^  HIC^δ^  BAC^ε^ | RP-UV | (López-Morales et al., 2015)^α, γ^, (Hausberger et al., 2016)^α, β^, (Jeong et al., 2018)^α, β^, (Lee et al., 2018a)^α, β, γ^, (Lee et al., 2018b)^α, β^, (Shekhawat et al., 2019)^α, β^, (McClellan et al., 2019)^α, γ^, (Saleem et al., 2020)^α, β^, (Zhang et al., 2020)^α, γ^, (Hutterer et al., 2021)^α, γ^ |
| **Process-related variants** | Host Cell Protein | kQA | ELISA^α^  LC^β^/ 2D LC-ESI-MS^γ^  2D-PAGE/ DIGE^δ^ | ELISA | (Rathore and Bhambure, 2014)^α, β, δ^, (Liu et al., 2016a)^α, γ, δ^, (Fang et al., 2016)^α, γ^ |
|  | Host Cell DNA | kQA |  |  |  |

**Table S7: CQA-wise methodological advancements and orthogonal tools applicable to similarity assessment exercise (since 2015)**

| **Quality attributes** | **Analytical tools** | **Methodologies** | **Orthogonal tools** | **Drug products** | **References** |
| --- | --- | --- | --- | --- | --- |
| Peptide mapping, paired glycoforms | nanoLC-MS/MS, IM-MS | Integrated top-down, middle down, bottom up, naive and IM-MS |  | Cetuximab, trastuzumab | (Beck et al., 2015) |
| Peptide mapping, glycopeptide, oxidation, deamidation, C-terminal lysine | 2D-LC-MS | Comprehensive (SCX x RP, RP x RP, HILIC x RP) | LC-MS | trastuzumab | (Vanhoenacker et al., 2015) |
| Peptide mapping, glycopeptide, deamidation, oxidation | LC-Orbitrap MS, Pinpoint software | MAM, LC-MS/MS followed by Pinpoint workbook formation for automated MS/MS analysis, relative quantification | HILIC-FLD, rCE-SDS | IgG1, anti-streptavidin IgG2 | (Rogers et al., 2015) |
| Peptide mapping | Chemometrics | Nearness Similarity Index, PCA, Manova | Coefficient of determination, cosine of the angle, Bray-Curtis distance and nearness index | Rituximab, Infliximab | (Pérez-Robles et al., 2017) |
| Peptide mapping, N-terminus, oxidation, deamidation | nLC-ion trap MS | integrated bottom-up and top-down LC-MS | RP-MS | r-hGH | (Wang et al., 2017) |
| Peptide mapping, paired glycoforms, reduced glycoforms, N-terminus, pyroE | LC- hybrid quadrupole orbitrap MS/MS | bottom up, middle up, intact and reduced analysis |  | anti-interleukin 8 IgG1 | (Farrell et al., 2018) |
| Peptide mapping, paired glycoform, C-terminal lysine loss | LC-QTOF-MS | middle up analysis |  | Infliximab | (Tsuda et al., 2018) |
| Peptide mapping | Chemometrics (RP-UV data) | PCA | visual assessment | r-hGH | (Shatat et al., 2018) |
| Peptide mapping, deamidation, oxidation, glycopeptide | LC-Orbitrap MS/MS | LC-MS/MS followed by targetted peptide monitoring, relative quantification, interlab-study | HILIC-FLD, RP-MS | NISTmAb | (Millán-Martín et al., 2020) |
| Peptide mapping, paired glycoforms, oxidation, deamidation | LC-orbitrap MS/MS | Multiple parallel protease digestion-RP-MS | LC-MS | Trastuzumab, Rituximab | (Pradhan et al., 2021) |
| Peptide mapping, deamidation, oxidation, isomerization, pyroE, glycopeptide | Automated digestion (STARline workstation)-LC-hybrid quadrupole orbitrap MS/MS | LC-MS/MS followed by targetted peptide monitoring, relative quantification, intersite-study | HILIC-FLD, RP-MS | IgG1, IgG4, multispecific, mAb fragment | (Song et al., 2021) |
| Charge variant, paired glycoform | IdeS-CZE-ESI-MS | offline CE-MS, intact and middle up analysis |  | cetuximab | (Biacchi et al., 2015) |
| Charge variant | cIEF | optimal resolution between pI 7-9 | CEX | multiple mAbs | (Suba et al., 2015) |
| Charge variant, paired glycoforms, reduced glycoforms, oxidation, c-terminal lysine, disulphide bonds | 2D selective LC x LC-ESI-TOF-MS | Intact and middle up (with and without reduction) analysis, CEX x RP | CEX-MS | Rituximab | (Stoll et al., 2015) |
| Charge variant | CZE | two phase four step method | CEX | mAb (Janssen) | (Suba et al., 2016) |
| Charge variant, paired glycoform, reduced glycoforms | 2D comprehensive LC x LC-ESI-TOF-MS | middle up analysis, CEX x RP-MS |  | Cetuximab, trastuzumab, infliximab | (Sorensen et al., 2016) |
| Charge variant | CEX | Salt based gradient |  | pertuzumab, adalimumab, belimumab, bevacizumab, denosumab, infliximab, ofatumuab, palivizumab, rituximab, trastuzumab | (Goyon et al., 2017) |
| Charge variant. paired glycoform, deamidation | 2D-heart cut-CZE-CZE-MS | Heart cutting, intact analysis | CEX-MS | trastuzumab | (Jooß et al., 2017) |
| Charge variant, paired glycoforms, C-terminal lysine forms, HOS | WCX-native-ESI-QTOF-MS-IM-MS | pH gradient CEX | cIEF, CEX, HILIC-ESI-MS-CID-MS/MS | Humanized IgG1k | (Sankaran et al., 2018) |
| Charge variant, paired glycoforms, C-terminal lysine forms, HOS | SCX-native-Orbitrap MS | pH gradient CEX | cIEF, CEX, HILIC-ESI-MS-CID-MS/MS | Trastuzumab, Adalimumab, infliximab, bevacizumab, cetuximab | (Füssl et al., 2018) |
| Charge variant, paired glycoform, isoaspartate formation | WCX-orbitrap MS | pH gradient native intact analysis followed by peptide mapping, Sliding Window algorithm |  | trastuzumab | (Bailey et al., 2018) |
| Charge variant, paired glycoforms, fragments, succinimide, deamidation, pyroGlu, C-terminal lysine | ZipChip-CE-ESI-MS | pH gradient Native Intact analysis | CEX-MS | Rituximab, trastuzumab, bevacizumab | (Carillo et al., 2020) |
| charge variant, paired glycoforms | ZipChip-CE-ESI-MS | pH gradient native intact analysis | CEX-MS | Cetuximab | (Fussl et al., 2020) |
| Charge variant | 2D-SCX-RP-ESI-QTOF-MS | Multiple heart cutting, comprehensive, ternary salt-mediated pH-gradient for SCX, intact and middle up analysis | CEX-MS | NISTmab, IgG1k | (Jaag et al., 2021) |
| Charge variant | CEX-MS data analysis | Sliding window algorithmic spectral deconvolution of charge variants |  | trastuzumab, adalimumab | (Millán-Martín et al., 2021) |
| HOS | NMR | 2D 13C, natural abundance, intact, subunit analysis | CD, FTIR | NISTmab | (Arbogast et al., 2015) |
| HOS | NMR | 1D analysis, logarithmic scale for PROFILE analysis | CD, FTIR | epoetin alpha, IgG1 | (Poppe et al., 2015) |
| HOS | Protein Conformational Array-ELISA |  |  | Rituximab | (Davies et al., 2015) |
| HOS | IM-MS | Native analysis | NMR, HDX-MS | anti TNFa | (Ferguson and Gucinski-Ruth, 2016) |
| HOS | NMR + Chemometrics | 1H-15N HSQC and 1H-1H NOESY, PCA |  | Filgrastim, rituximab | (Japelj et al., 2016) |
| HOS | NMR + Chemometrics | 2D 1H-15N, CCSD, PCA | CD, FTIR | Filgrastim | (Ghasriani et al., 2016) |
| HOS | NMR | 2D 1H-15N, intact, sub unit analysis, statistical analysis | CD, FTIR | NISTmAb, IgG1k | (Arbogast et al., 2016) |
| HOS | HDX-MS |  |  | angiotensin II, insulin, Interferon-b-1a | (E. Nazari et al., 2016) |
| HOS/ Stability | CSD |  |  |  | (Kim et al., 2016a) |
| HOS | NMR + Chemometrics | 2D 1H-13C methyl correlated, PCA | CD, FTIR | NISTmab | (Arbogast et al., 2017) |
| HOS | HT-Protein Conformational Array |  | SEC, thermal shift assay | IgG1, IgG2 | (Song et al., 2018) |
| HOS | NMR + Chemometrics | 2D 1H-13C methyl correlated, SIEERA filter, PCA | CD, FTIR | NISTmab | (Arbogast et al., 2017) |
| HOS | NMR + Chemometrics | 1D 1H NMR, 2D 1H-13C NMR, PCA, 3-way Tucker3/graph invariant (GI), mahalanobis distance |  |  | (Chen et al., 2018) |
| HOS | NMR | 2D J-correlated NMR, 1Hn-1Hα correlations | CD, FTIR | Filgrastim | (Brinson and Marino, 2019) |
| HOS | HDX-MS | Fab analysis, Interlab study |  | NISTmab | (Hudgens et al., 2019) |
| HOS | HDX-MS + statistics | hybrid significance testing, volcano plot analysis |  | maltose binding protein, infliximab | (Hageman and Weis, 2019) |
| HOS, oxidation, disulfide scrambling | RP-MS | MRM, radical footprinting |  | Bevacizumab, Denosumab | (Yi et al., 2019) |
| HOS | Aptamer based ELISA | mAb specific aptamers |  | Rituximab | (Wildner et al., 2019) |
| HOS | XRC |  | NMR | infliximab | (Lerch et al., 2020) |
| HOS | NMR | 1D-PROFILE, 2D 13C, interlab comparison study, correlation analysis | CD, FTIR | NISTmab | (Elliott et al., 2020) |
| HOS | CD + Chemometrics | far and near UV-CD, weighted spectral difference, correlation coefficient, and area of overlap |  | trastuzumab | (Fang et al., 2020) |
| HOS | NMR + Chemometrics | 2D-NMR, chemometric outlier classification, interlab study |  | NISTmAb | (Sheen et al., 2020) |
| HOS | NMR + Chemometrics | 2D-NMR, PCA, automated classification |  | NISTmab | (Brinson et al., 2020) |
| HOS | Receptor binding HDX MS |  |  | filgrastim, pegfilgrastim | (Treuheit et al., 2020) |
| Size, HOS | SAXS |  | SEC, AUC, DLS, CD, FLD | Rituximab | (Narvekar et al., 2020) |
| HOS/ Stability | NanoDSF |  | DSC | mAbs, BiTE® molecules, cytokines | (Wen et al., 2020) |
| HOS | NanoDSF |  | DSC | trastuzumab, rituximab | (Joshi et al., 2020) |
| HOS | NMR + Chemometrics | 1D 1H NMR, PCA, mahalanobis distance | CD, FTIR | insulin | (Wang et al., 2020) |
| HOS | HDX-MS + statistics | statistical equivalence testing |  | maltose binding protein, infliximab | (Hageman et al., 2021) |
| Size | SEC |  | phosphate buffer (pH 6.8), 0.35 ml/min, isocratic elution | Infliximab, pertuzumab | (Goyon et al., 2017) |
| Size | NMR | DOSY-NMR | DLS | Insulin | (Patil et al., 2017) |
| Size |  | 1D- phosphate buffer (separation), 2D-100 mM ammonium acetate buffer (desalting), isocratic |  | pembrolizumab, ipilimumab, bevacizumab | (Ehkirch et al., 2018) |
| Size | TEM | Semi-automated, size-based clustering | SEC, DLS | anti-CD20 mAb | (Kumar et al., 2020) |
| Size | SV-AUC |  | SEC, DLS | Rituximab | (Patil et al., 2020) |
| Size | nrCE-SDS | non reducing intact analysis | SEC | 26 FDA and EMA approved mAbs, NISTmAB | (Wagner et al., 2020) |
| Size | NMR | DOSY-NMR | DLS | trastuzumab | (Joshi et al., 2021a) |
| Glycan | Lectin Microarray | Partial digestion/degradation | LC-FLD | IgG | (Cook et al., 2015) |
| Glycan monosaccharide | LC-MS | MRM using HILIC-MS/MS, underivatized monosaccharide analysis, | HPAEC-PAD | NISTmAb, IgG (human serum), IgG3, RNAse B | (Lowenthal et al., 2015) |
| Glycan | Spectroscopy + ML | Fluorescence (intrinsic, extrinsic), turbidity, k-nearest neighbour, support vector machine, linear discriminant analysis, quadratic discriminant analysis, Naïve Bayse, Decision Tree, random forest, AdaBoosted DTs, | HILIC | IgG | (Kim et al., 2016b) |
| Paired glycoforms, glycopeptides | Hybird MS + biosimilarity score | high-resolution native mass spectrometry and middle-down proteomics, quantitative similarity assessment |  | erythropoietin, human plasma properdin | (Yang et al., 2016) |
| Glycan | LC-MS | IdeS-RPLC-MS, middle down analysis | Free glycan analysis | IgG1 biosimilar candidate | (Liu et al., 2016b) |
| Paired glycoforms, reduced glycoforms | LC-MS | IdeS/ EndoS/ EndoS2-RPC-MS, middle up analysis |  | trastuzumab | (Upton et al., 2016) |
| Intact glycan, released glycan, glycopeptide | LC-MS | HILIC-ESI-QTOF, mixed-mode, HPC and RPC |  | adalimumab, entanercept | (Largy et al., 2017) |
| Glycan | LC-MS | derivatization, HILIC-UPLC-FLD/MS |  | NISTmAb | (Hilliard et al., 2017) |
| Released glycan, glycopeptide | LC-MS | middle up analysis, HILIC-MS | HILIC-FLD | Infliximab, Trastuzumab, Cetuximab | (D’Atri et al., 2017) |
| Released Glycan | LC-MS, chemometrics | HILIC-MS-PCA/ SIMCA, soft independent modelling by class analogy |  | trastuzumab, human plasma IgG | (Planinc et al., 2017a) |
| Major glycan monosaccharides | NMR | 2D 1H–13C NMR, middle down analysis | HILIC-FLD | rituximab, infliximab, bevacizumab, etanercept, adalimumab | (Peng et al., 2018) |
| Glycopeptide | CE-ESI-MS | Relative quantification | HILIC-FLD | adalimumab, infliximab, trastuzumab, palivizumab, natalizumab, rituximab, nivolumab, panitumumab | (Giorgetti et al., 2018) |
| Released glycan | CGE | quantitative glycosimilarity | HILIC-FLD | etanercept | (Borza et al., 2018) |
| Glycan | MoFi: glycan annotation | Hybrid data analysis (intact and glycoprotein |  | rituxumab, edo-trastuzumab-emtansine, human erythropoetin | (Skala et al., 2018) |
| Intact glycan | MS | Native-orbitrap MS |  | erythropoetin | (Čaval et al., 2018) |
| Glycan | Data interpretation | GUcal (structural annotation of CE data) |  |  | (Jarvas et al., 2018) |
| Paired glycoforms, reduced glycoforms | LC-MS | HILIC-ESI-QTOF-MS, middle up analysis | RPLC-MS | etanercept | (D’Atri et al., 2018) |
| Glycan, monosaccharide profile | FTIR, chemometrics | buffer exchange, ATR-FTIR, PCA | HILIC-FLD | 14 therapeutic mAbs, alpha1-acid glycoprotein, ribonuclease B, avidin, fetuin | (Derenne et al., 2020) |
| Glycan | Glycosimilarity index |  |  | IgG1k | (Szekrenyes et al., 2020) |
| Paired glycoforms/ Identity | LC-MS | RP-ESI-IM-QTOF-MS, intact and de-glycosylated analysis |  | rituximab | (Perdomo-Abúndez et al., 2020) |
| Glycan | Multivariate statistics | JMP based distance between originator and biosimilar |  | IgG1 | (Xu et al., 2021) |
| Oxidation | IP-RP-UV, IP-RP-Orbitrap MS, IP-RP-AIF-MS | middle down analysis in reduced conditions via 3 strategies, absolute quantification | peptide mapping | Rituximab, adalimumab, etanercept | (Regl et al., 2017) |
| Glycation | LC-MS | BAC-MS, quantitative analysis |  | mAb | (Viski et al., 2016) |
| Glycation | MS-MS | Sodium borohydride derivatization-CID-MS/MS |  | mAb | (Saleem et al., 2015) |
| HCP | LC-MS | protein A depletion, multifactorial LC-MS/MS |  | NISTmAb | (Johnson et al., 2020) |
| Disulphide | LC-MS | LC-Orbitrap MS with UVPD dissociation |  | Rituximab | (Bonner et al., 2018) |
| Disulphide | LC-EC-MS | Bottom-up workflow, FTICR-MS |  | Ribonuclease B, ß-lactoglobulin | (Switzar et al., 2016) |
| Free -SH content | SoloVPE | Ellman reagent derivatization, VIS detection (412 nm) |  | mAbs | (Zhang and Qi, 2021) |
| Effector binding | LC | FcRγ affinity chromatography |  | trastuzumab | (Xie et al., 2020) |
| Carbonylation | RP-ESI-MS/MS | FTC derivatization of intact mAb followed by peptide mapping | DNPH, GRP derivatization | Trastuzumab | (Joshi et al., 2021b) |
| Statistics | Statistics: Tolerance interval |  |  |  | (Chen and Hsiao, 2020) |
|  | Fiducial inference based simultaneous confidence interval method |  |  |  | (Zheng et al., 2019) |
|  | Confidence interval |  |  |  | (Quiroz et al., 2019) |
|  | statistical analysis |  |  |  | (Lee et al., 2018a) |
|  | Tolerance interval and biosimilarity index |  |  |  | (Chen et al., 2017) |
|  | Equivalence acceptance criterion |  |  |  | (Wang and Chow, 2017) |
|  | Exact test-based approach |  |  |  | (Dong et al., 2017) |
|  |  |  |  |  | (Burdick et al., 2017) |
|  | CSD data visualization |  |  |  | (Kim et al., 2016a) |

**References**

Alley, W., Tao, L., Shion, H., Yu, Y. Q., Rao, C., and Chen, W. (2020). UPLC-MS assessment on the structural similarity of recombinant human erythropoietin (rhEPO) analogues from manufacturers in China for attribute monitoring. *Talanta* 220. doi:10.1016/j.talanta.2020.121335.

An, Q., Zheng, Y., Zhao, Y., Liu, T., Guo, H., Zhang, D., et al. (2019). Physicochemical characterization and phase i study of CMAB008, an infliximab biosimilar produced by a different expression system. *Drug Des. Devel. Ther.* 13, 791–805. doi:10.2147/DDDT.S170913.

Arbogast, L. W., Brinson, R. G., Formolo, T., Hoopes, J. T., and Marino, J. P. (2016). 2D 1HN, 15N Correlated NMR Methods at Natural Abundance for Obtaining Structural Maps and Statistical Comparability of Monoclonal Antibodies. *Pharm. Res.* 33, 462–475. doi:10.1007/s11095-015-1802-3.

Arbogast, L. W., Brinson, R. G., and Marino, J. P. (2015). Mapping Monoclonal Antibody Structure by 2D 13C NMR at Natural Abundance. *Anal. Chem.* 87, 3556–3561. doi:10.1021/ac504804m.

Arbogast, L. W., Delaglio, F., Schiel, J. E., and Marino, J. P. (2017). Multivariate Analysis of Two-Dimensional 1H, 13C Methyl NMR Spectra of Monoclonal Antibody Therapeutics To Facilitate Assessment of Higher Order Structure. *Anal. Chem.* 89, 11839–11845. doi:10.1021/acs.analchem.7b03571.

Arvinte, T., Palais, C., Poirier, E., Cudd, A., Rajendran, S., Brokx, S., et al. (2019a). Part 1: Physicochemical characterization of bevacizumab in undiluted 25 mg/mL drug product solutions: Comparison of originator with a biosimilar candidate. *J. Pharm. Biomed. Anal.* 175. doi:10.1016/j.jpba.2019.06.039.

Arvinte, T., Palais, C., Poirier, E., Cudd, A., Rajendran, S., Brokx, S., et al. (2019b). Part 2: Physicochemical characterization of bevacizumab in 2 mg/mL antibody solutions as used in human i.v. administration: Comparison of originator with a biosimilar candidate. *J. Pharm. Biomed. Anal.* 176. doi:10.1016/j.jpba.2019.112802.

Bailey, A. O., Han, G., Phung, W., Gazis, P., Sutton, J., Josephs, J. L., et al. (2018). Charge variant native mass spectrometry benefits mass precision and dynamic range of monoclonal antibody intact mass analysis. *MAbs* 10, 1214–1225. doi:10.1080/19420862.2018.1521131.

Beck, A., Debaene, F., Diemer, H., Wagner-Rousset, E., Colas, O., Dorsselaer, A. Van, et al. (2015). Cutting-edge mass spectrometry characterization of originator, biosimilar and biobetter antibodies. *J. Mass Spectrom.* 50, 285–297. doi:10.1002/jms.3554.

Beyer, B., Walch, N., Jungbauer, A., and Lingg, N. (2019). How Similar Is Biosimilar? A Comparison of Infliximab Therapeutics in Regard to Charge Variant Profile and Antigen Binding Affinity. *Biotechnol. J.* 14. doi:10.1002/biot.201800340.

Biacchi, M., Gahoual, R., Said, N., Beck, A., Leize-Wagner, E., and François, Y. N. (2015). Glycoform Separation and Characterization of Cetuximab Variants by Middle-up Off-Line Capillary Zone Electrophoresis-UV/Electrospray Ionization-MS. *Anal. Chem.* 87, 6240–6250. doi:10.1021/acs.analchem.5b00928.

Bonner, J., Talbert, L. E., Akkawi, N., and Julian, R. R. (2018). Simplified identification of disulfide, trisulfide, and thioether pairs with 213 nm UVPD. *Analyst* 143, 5176–5184. doi:10.1039/c8an01582a.

Bor Tekdemir, Z., Seckin, A. I., Kacar, T., Yilmaz, E., and Bekiroglu, S. (2020). Evaluation of Structural, Biological, and Functional Similarity of Biosimilar Granulocyte Colony Stimulating Factor to its Reference Product. *Pharm. Res.* 37. doi:10.1007/s11095-020-02932-7.

Borza, B., Szigeti, M., Szekrenyes, A., Hajba, L., and Guttman, A. (2018). Glycosimilarity assessment of biotherapeutics 1: Quantitative comparison of the N-glycosylation of the innovator and a biosimilar version of etanercept. *J. Pharm. Biomed. Anal.* 153, 182–185. doi:10.1016/j.jpba.2018.02.021.

Brinks, V., Hawe, A., Basmeleh, A. H. H., Joachin-Rodriguez, L., Haselberg, R., Somsen, G. W., et al. (2011). Quality of original and biosimilar epoetin products. *Pharm. Res.* 28, 386–393. doi:10.1007/s11095-010-0288-2.

Brinson, R. G., Elliott, K. W., Arbogast, L. W., Sheen, D. A., Giddens, J. P., Marino, J. P., et al. (2020). Principal component analysis for automated classification of 2D spectra and interferograms of protein therapeutics: influence of noise, reconstruction details, and data preparation. *J. Biomol. NMR* 74, 643–656. doi:10.1007/s10858-020-00332-y.

Brinson, R. G., and Marino, J. P. (2019). 2D J-correlated proton NMR experiments for structural fingerprinting of biotherapeutics. *J. Magn. Reson.* 307, 106581. doi:10.1016/J.JMR.2019.106581.

Brokx, S., Scrocchi, L., Shah, N., and Dowd, J. (2017). A demonstration of analytical similarity comparing a proposed biosimilar pegfilgrastim and reference pegfilgrastim. *Biologicals* 48, 28–38. doi:10.1016/j.biologicals.2017.06.001.

Brown, K. A., Rajendran, S., Dowd, J., and Wilson, D. J. (2019). Rapid characterization of structural and functional similarity for a candidate bevacizumab (Avastin) biosimilar using a multipronged mass-spectrometry-based approach. *Drug Test. Anal.* 11, 1207–1217. doi:10.1002/dta.2609.

Burdick, R., Coffey, T., Gutka, H., Gratzl, G., Conlon, H. D., Huang, C. T., et al. (2017). Statistical Approaches to Assess Biosimilarity from Analytical Data. *AAPS J.* 19, 4–14. doi:10.1208/s12248-016-9968-0.

Carillo, S., Jakes, C., and Bones, J. (2020). In-depth analysis of monoclonal antibodies using microfluidic capillary electrophoresis and native mass spectrometry. *J. Pharm. Biomed. Anal.* 185, 113218. doi:10.1016/j.jpba.2020.113218.

Čaval, T., Tian, W., Yang, Z., Clausen, H., and Heck, A. J. R. (2018). Direct quality control of glycoengineered erythropoietin variants. *Nat. Commun.* 9, 1–8. doi:10.1038/s41467-018-05536-3.

Cerutti, M. L., Pesce, A., Bès, C., and Seigelchifer, M. (2019). Physicochemical and Biological Characterization of RTXM83, a New Rituximab Biosimilar. *BioDrugs* 33, 307–319. doi:10.1007/s40259-019-00349-2.

Chen, C., and Hsiao, C.-F. (2020). Use of tolerance intervals for assessing biosimilarity. *Stat. Med.* 39, 3806–3822. doi:10.1002/SIM.8695.

Chen, C. T., Tsou, H. H., Hsiao, C. F., Lai, Y. H., Chang, W. J., and Liu, J. T. (2017). A Tolerance Interval Approach to Assessing the Biosimilarity of Follow-On Biologics. *Stat. Biopharm. Res.* 9, 286–292. doi:10.1080/19466315.2017.1323669.

Chen, K., Park, J., Li, F., Patil, S. M., and Keire, D. A. (2018). Chemometric Methods to Quantify 1D and 2D NMR Spectral Differences Among Similar Protein Therapeutics. *AAPS PharmSciTech* 19, 1011–1019. doi:10.1208/s12249-017-0911-1.

Chen, L., Wang, L., Shion, H., Yu, C., Yu, Y. Q., Zhu, L., et al. (2016). In-depth structural characterization of Kadcyla® (ado-trastuzumab emtansine) and its biosimilar candidate. *MAbs* 8, 1210–1223. doi:10.1080/19420862.2016.1204502.

Chen, S. H. L., Wu, S. L., Huang, L. J., Huang, J. B., and Chen, S. H. L. (2013). A global comparability approach for biosimilar monoclonal antibodies using LC-tandem MS based proteomics. *J. Pharm. Biomed. Anal.* 80, 126–135. doi:10.1016/j.jpba.2013.02.040.

Cho, I. H., Lee, N., Song, D., Jung, S. Y., Bou-Assaf, G., Sosic, Z., et al. (2016). Evaluation of the structural, physicochemical, and biological characteristics of SB4, a biosimilar of etanercept. *MAbs* 8, 1136–1155. doi:10.1080/19420862.2016.1193659.

Cook, M. C., Kaldas, S. J., Muradia, G., Rosu-Myles, M., and Kunkel, J. P. (2015). Comparison of orthogonal chromatographic and lectin-affinity microarray methods for glycan profiling of a therapeutic monoclonal antibody. *J. Chromatogr. B Anal. Technol. Biomed. Life Sci.* 997, 162–178. doi:10.1016/j.jchromb.2015.05.035.

Crobu, D., Spinetti, G., Schrepfer, R., Tonon, G., Jotti, G. S., Onali, P., et al. (2014). Preclinical and clinical phase I studies of a new recombinant Filgrastim (BK0023) in comparison with Neupogen®. *BMC Pharmacol. Toxicol.* 15, 1–13. doi:10.1186/2050-6511-15-7.

D’Atri, V., Fekete, S., Beck, A., Lauber, M., and Guillarme, D. (2017). Hydrophilic Interaction Chromatography Hyphenated with Mass Spectrometry: A Powerful Analytical Tool for the Comparison of Originator and Biosimilar Therapeutic Monoclonal Antibodies at the Middle-up Level of Analysis. *Anal. Chem.* 89, 2086–2092. doi:10.1021/acs.analchem.6b04726.

D’Atri, V., Nováková, L., Fekete, S., Stoll, D., Lauber, M., Beck, A., et al. (2018). Orthogonal Middle-up Approaches for Characterization of the Glycan Heterogeneity of Etanercept by Hydrophilic Interaction Chromatography Coupled to High-Resolution Mass Spectrometry. *Anal. Chem.* 91, 873–880. doi:10.1021/ACS.ANALCHEM.8B03584.

Da Silva, A., Kronthaler, U., Koppenburg, V., Fink, M., Meyer, I., Papandrikopoulou, A., et al. (2014). Target-directed development and preclinical characterization of the proposed biosimilar rituximab GP2013. *Leuk. Lymphoma* 55, 1609–1617. doi:10.3109/10428194.2013.843090.

Davies, M., Wang, G., Fu, G., and Wang, X. (2015). mAb Higher Order Structure Analysis with Protein Conformational Array ELISA. *Br. J. Pharm. Res.* 7, 401–412. doi:10.9734/bjpr/2015/18952.

Deechongkit, S., Aoki, K. H., Park, S. S., and Kerwin, B. A. (2006). Biophysical comparability of the same protein from different manufacturers: A case study using Epoetin alfa from Epogen® and Eprex®. *J. Pharm. Sci.* 95, 1931–1943. doi:10.1002/jps.20649.

Derenne, A., Derfoufi, K. M., Cowper, B., Delporte, C., and Goormaghtigh, E. (2020). FTIR spectroscopy as an analytical tool to compare glycosylation in therapeutic monoclonal antibodies. *Anal. Chim. Acta* 1112, 62–71. doi:10.1016/j.aca.2020.03.038.

Derzi, M., Johnson, T. R., Shoieb, A. M., Conlon, H. D., Sharpe, P., Saati, A., et al. (2016). Nonclinical Evaluation of PF-06438179: A Potential Biosimilar to Remicade® (Infliximab). *Adv. Ther.* 33, 1964–1982. doi:10.1007/s12325-016-0403-9.

Derzi, M., Shoieb, A. M., Ripp, S. L., Finch, G. L., Lorello, L. G., O’Neil, S. P., et al. (2020). Comparative nonclinical assessments of the biosimilar PF-06410293 and originator adalimumab. *Regul. Toxicol. Pharmacol.* 112. doi:10.1016/j.yrtph.2020.104587.

Dong, X. C., Bian, Y., Tsong, Y., and Wang, T. (2017). Exact test-based approach for equivalence test with parameter margin. *J. Biopharm. Stat.* 27, 317–330. doi:10.1080/10543406.2016.1265546.

E. Nazari, Z., van de Weert, M., Bou-Assaf, G., Houde, D., Weiskopf, A., and D. Rand, K. (2016). Rapid Conformational Analysis of Protein Drugs in Formulation by Hydrogen/Deuterium Exchange Mass Spectrometry. *J. Pharm. Sci.* 105, 3269–3277. doi:10.1016/j.xphs.2016.07.006.

Ehkirch, A., Goyon, A., Hernandez-Alba, O., Rouviere, F., D’Atri, V., Dreyfus, C., et al. (2018). A Novel Online Four-Dimensional SEC×SEC-IM×MS Methodology for Characterization of Monoclonal Antibody Size Variants. *Anal. Chem.* 90, 13929–13937. doi:10.1021/acs.analchem.8b03333.

Elliott, K. W., Ghasriani, H., Wikström, M., Giddens, J. P., Aubin, Y., Delaglio, F., et al. (2020). Comparative Analysis of One-Dimensional Protein Fingerprint by Line Shape Enhancement and Two-Dimensional 1H,13C Methyl NMR Methods for Characterization of the Higher Order Structure of IgG1 Monoclonal Antibodies. *Anal. Chem.* 92, 6366–6373. doi:10.1021/acs.analchem.9b05385.

Escobedo-Moratilla, A., Kuri-Breña Romero De Terreros, F., Pérez-Urizar, J., and Barba De La Rosa, A. P. (2016). Analytical and Biological Characterization of a Noninnovator Insulin Glargine and the Originator Drug Product. *J. Diabetes Sci. Technol.* 10, 616–617. doi:10.1177/1932296815606914.

Fang, J., Doneanu, C., Alley, W. R., Yu, Y. Q., Beck, A., and Chen, W. (2016). Advanced assessment of the physicochemical characteristics of Remicade® and Inflectra® by sensitive LC/MS techniques. *MAbs* 8, 1021–1034. doi:10.1080/19420862.2016.1193661.

Fang, J., Li, H., Wu, S., and Dong, F. (2020). Higher-Order Structure Comparison of a Proposed Biosimilar and the Innovator Biotherapeutic Trastuzumab using Circular Dichroism Coupled with Statistical Analysis. *J. Appl. Spectrosc.* 87, 938–945. doi:10.1007/s10812-020-01092-1.

Farrell, A., Carillo, S., Scheffler, K., Cook, K., and Bones, J. (2018). Monoclonal antibody sequence assessment using a hybrid quadrupole-Orbitrap mass spectrometer. *Anal. Methods* 10, 3100–3109. doi:10.1039/c8ay00582f.

Fazel, R., Guan, Y., Vaziri, B., Krisp, C., Heikaus, L., Saadati, A., et al. (2019). Structural and in vitro functional comparability analysis of altebrel^TM^, a proposed etanercept biosimilar: Focus on primary sequence and glycosylation. *Pharmaceuticals* 12. doi:10.3390/ph12010014.

Ferguson, C. N., and Gucinski-Ruth, A. C. (2016). Evaluation of Ion Mobility-Mass Spectrometry for Comparative Analysis of Monoclonal Antibodies. *J. Am. Soc. Mass Spectrom.* 27, 822–833. doi:10.1007/s13361-016-1369-1.

Flores-Ortiz, L. F., Campos-García, V. R., Perdomo-Abúndez, F. C., Pérez, N. O., and Medina-Rivero, E. (2014). Physicochemical properties of Rituximab. *J. Liq. Chromatogr. Relat. Technol.* 37, 1438–1452. doi:10.1080/10826076.2013.794738.

Füssl, F., Cook, K., Scheffler, K., Farrell, A., Mittermayr, S., and Bones, J. (2018). Charge Variant Analysis of Monoclonal Antibodies Using Direct Coupled pH Gradient Cation Exchange Chromatography to High-Resolution Native Mass Spectrometry. *Anal. Chem.* 90, 4669–4676. doi:10.1021/acs.analchem.7b05241.

Fussl, F., Trappe, A., Carillo, S., Jakes, C., and Bones, J. (2020). Comparative Elucidation of Cetuximab Heterogeneity on the Intact Protein Level by Cation Exchange Chromatography and Capillary Electrophoresis Coupled to Mass Spectrometry. *Anal. Chem.* 92, 5431–5438. doi:10.1021/acs.analchem.0c00185.

Ghasriani, H., Hodgson, D. J., Brinson, R. G., McEwen, I., Buhse, L. F., Kozlowski, S., et al. (2016). Precision and robustness of 2D-NMR for structure assessment of filgrastim biosimilars. *Nat. Biotechnol.* 34, 139–141. doi:10.1038/nbt.3474.

Gianoncelli, A., Bertuzzi, M., Guarienti, M., Vezzoli, S., Bonini, S. A., Mastinu, A., et al. (2019). Parallelism of Chemicostructural Properties between Filgrastim Originator and Three of Its Biosimilar Drugs. *J. Chem.* 2019. doi:10.1155/2019/2751461.

Giorgetti, J., D’Atri, V., Canonge, J., Lechner, A., Guillarme, D., Colas, O., et al. (2018). Monoclonal antibody N-glycosylation profiling using capillary electrophoresis – Mass spectrometry: Assessment and method validation. *Talanta* 178, 530–537. doi:10.1016/j.talanta.2017.09.083.

Goyal, P., Pai, H. V., Kodali, P., Vats, B., Vajpai, N., Annegowda, S., et al. (2021). Physicochemical and functional characterization of MYL-1501D, a proposed biosimilar to insulin glargine. *PLoS One* 16, e0253168. doi:10.1371/JOURNAL.PONE.0253168.

Goyon, A., D’Atri, V., Bobaly, B., Wagner-Rousset, E., Beck, A., Fekete, S., et al. (2017). Protocols for the analytical characterization of therapeutic monoclonal antibodies. I – Non-denaturing chromatographic techniques. *J. Chromatogr. B Anal. Technol. Biomed. Life Sci.* 1058, 73–84. doi:10.1016/j.jchromb.2017.05.010.

Guyader, G. Le, Vieillard, V., and Paul, M. (2020). Physicochemical stability study of MYL-1401O, a biosimilar of trastuzumab, following a transient temperature excursion. *J. Oncol. Pharm. Pract.* doi:10.1177/1078155220940410.

Hageman, T. S., and Weis, D. D. (2019). Reliable Identification of Significant Differences in Differential Hydrogen Exchange-Mass Spectrometry Measurements Using a Hybrid Significance Testing Approach. *Anal. Chem* 91, 8016. doi:10.1021/acs.analchem.9b01325.

Hageman, T. S., Wrigley, M. S., and Weis, D. D. (2021). Statistical Equivalence Testing of Higher-Order Protein Structures with Differential Hydrogen Exchange-Mass Spectrometry (HX-MS). *Anal. Chem.*, acs.analchem.0c05279. doi:10.1021/acs.analchem.0c05279.

Halim, L. A., Brinks, V., Jiskoot, W., Romeijn, S., Haselberg, R., Burns, C., et al. (2016). Quality and Batch-to-Batch Consistency of Original and Biosimilar Epoetin Products. *J. Pharm. Sci.* 105, 542–550. doi:10.1016/j.xphs.2015.10.019.

Halim, L. A., Brinks, V., Jiskoot, W., Romeijn, S., Praditpornsilpa, K., Assawamakin, A., et al. (2014). How bio-questionable are the different recombinant human erythropoietin copy products in Thailand? *Pharm. Res.* 31, 1210–1218. doi:10.1007/s11095-013-1243-9.

Halim, L. A., Márquez, M., Maas-Bakker, R. F., Castañeda-Hernández, G., Jiskoot, W., and Schellekens, H. (2018). Quality Comparison of Biosimilar and Copy Filgrastim Products with the Innovator Product. *Pharm. Res.* 35. doi:10.1007/s11095-018-2491-5.

Harazono, A., Hashii, N., Kuribayashi, R., Nakazawa, S., and Kawasaki, N. (2013). Mass spectrometric glycoform profiling of the innovator and biosimilar erythropoietin and darbepoetin by LC/ESI-MS. *J. Pharm. Biomed. Anal.* 83, 65–74. doi:10.1016/j.jpba.2013.04.031.

Hashii, N., Harazono, A., Kuribayashi, R., Takakura, D., and Kawasaki, N. (2014). Characterization of N-glycan heterogeneities of erythropoietin products by liquid chromatography/mass spectrometry and multivariate analysis. *Rapid Commun. Mass Spectrom.* 28, 921–932. doi:10.1002/rcm.6858.

Hassan, L. A., Shatat, S. M., Eltanany, B. M., Al-Ghobashy, M. A., and Abbas, S. S. (2019). Stability and biosimilarity assessment of infliximab using an orthogonal testing protocol and statistically-guided interpretation of peptide mapping. *Anal. Methods* 11, 3198–3211. doi:10.1039/c9ay00903e.

Hassett, B., Scheinberg, M., Castañeda-Hernández, G., Li, M., Rao, U. R. K., Singh, E., et al. (2018). Variability of intended copies for etanercept (Enbrel®): Data on multiple batches of seven products. *MAbs* 10, 166–176. doi:10.1080/19420862.2017.1387346.

Hausberger, A., Lamanna, W. C., Hartinger, M., Seidl, A., Toll, H., and Holzmann, J. (2016). Identification of Low-Level Product-Related Variants in Filgrastim Products Presently Available in Highly Regulated Markets. *BioDrugs* 30, 233–242. doi:10.1007/s40259-016-0169-2.

Hermosilla, J., Pérez-Robles, R., Salmerón-García, A., Casares, S., Cabeza, J., Bones, J., et al. (2020). Comprehensive biophysical and functional study of ziv-aflibercept: characterization and forced degradation. *Sci. Rep.* 10, 1–13. doi:10.1038/s41598-020-59465-7.

Hermosilla, J., Sánchez-Martín, R., Pérez-Robles, R., Salmerón-García, A., Casares, S., Cabeza, J., et al. (2019). Comparative Stability Studies of Different Infliximab and Biosimilar CT-P13 Clinical Solutions by Combined Use of Physicochemical Analytical Techniques and Enzyme-Linked Immunosorbent Assay (ELISA). *BioDrugs* 33, 193–205. doi:10.1007/s40259-019-00342-9.

Hilliard, M., Jr., W. R. A., McManus, C. A., Yu, Y. Q., Hallinan, S., Gebler, J., et al. (2017). Glycan characterization of the NIST RM monoclonal antibody using a total analytical solution: From sample preparation to data analysis. *https://doi.org/10.1080/19420862.2017.1377381* 9, 1349–1359. doi:10.1080/19420862.2017.1377381.

Hong, J., Lee, Y., Lee, C., Eo, S., Kim, S., Lee, N., et al. (2017). Physicochemical and biological characterization of SB2, a biosimilar of Remicade® (infliximab). *MAbs* 9, 364–382. doi:10.1080/19420862.2016.1264550.

Huang, L. J., Chiang, C. W., Lee, Y. W., Wang, T. F., Fong, C. C., and Chen, S. H. (2016). Characterization and comparability of stress-induced oxidation and deamidation on vulnerable sites of etanercept products. *J. Chromatogr. B Anal. Technol. Biomed. Life Sci.* 1032, 189–197. doi:10.1016/j.jchromb.2016.05.007.

Hudgens, J. W., Gallagher, E. S., Karageorgos, I., Anderson, K. W., Filliben, J. J., Huang, R. Y.-C., et al. (2019). Interlaboratory Comparison of Hydrogen–Deuterium Exchange Mass Spectrometry Measurements of the Fab Fragment of NISTmAb. *Anal. Chem.* 91, 7336–7345. doi:10.1021/ACS.ANALCHEM.9B01100.

Hurst, S., Ryan, A. M., Ng, C. K., McNally, J. M., Lorello, L. G., Finch, G. L., et al. (2014). Comparative nonclinical assessments of the proposed biosimilar PF-05280014 and trastuzumab (Herceptin®). *BioDrugs* 28, 451–459. doi:10.1007/s40259-014-0103-4.

Hutterer, K. M., Ip, A., Kuhns, S., Cao, S., Wikström, M., and Liu, J. (2021). Analytical Similarity Assessment of ABP 959 in Comparison with Eculizumab Reference Product. *BioDrugs 2021*, 1–15. doi:10.1007/S40259-021-00492-9.

Hutterer, K. M., Polozova, A., Kuhns, S., McBride, H. J., Cao, X., and Liu, J. (2019). Assessing Analytical and Functional Similarity of Proposed Amgen Biosimilar ABP 980 to Trastuzumab. *BioDrugs* 33, 321–333. doi:10.1007/s40259-019-00350-9.

Jaag, S., Shirokikh, M., and Lämmerhofer, M. (2021). Charge variant analysis of protein-based biopharmaceuticals using two-dimensional liquid chromatography hyphenated to mass spectrometry. *J. Chromatogr. A* 1636, 461786. doi:10.1016/j.chroma.2020.461786.

Japelj, B., Ilc, G., Marušič, J., Senčar, J., Kuzman, D., and Plavec, J. (2016). Biosimilar structural comparability assessment by NMR: From small proteins to monoclonal antibodies. *Sci. Rep.* 6, 1–12. doi:10.1038/srep32201.

Jarvas, G., Szigeti, M., and Guttman, A. (2018). Structural identification of N-linked carbohydrates using the GUcal application: A tutorial. *J. Proteomics* 171, 107–115. doi:10.1016/J.JPROT.2017.08.017.

Jeong, Y. R., Jeong, R. U., Son, J. H., Kwon, J. C., Jung, S., Song, M. A., et al. (2018). Comprehensive Physicochemical and Biological Characterization of the Proposed Biosimilar Darbepoetin Alfa, LBDE, and Its Originator Darbepoetin Alfa, NESP®. *BioDrugs* 32, 153–168. doi:10.1007/s40259-018-0272-7.

Johnson, R. O. B., Greer, T., Cejkov, M., Zheng, X., and Li, N. (2020). Combination of FAIMS, Protein A Depletion, and Native Digest Conditions Enables Deep Proteomic Profiling of Host Cell Proteins in Monoclonal Antibodies. *Anal. Chem.* 92, 10478–10484. doi:10.1021/acs.analchem.0c01175.

Jooß, K., Hühner, J., Kiessig, S., Moritz, B., and Neusüß, C. (2017). Two-dimensional capillary zone electrophoresis–mass spectrometry for the characterization of intact monoclonal antibody charge variants, including deamidation products. *Anal. Bioanal. Chem.* 409, 6057–6067. doi:10.1007/s00216-017-0542-0.

Joshi, S., Khatri, L. R., Kumar, A., and Rathore, A. S. (2021a). Monitoring size and oligomeric-state distribution of therapeutic mAbs by NMR and DLS: Trastuzumab as a case study. *J. Pharm. Biomed. Anal.* 195, 113841. doi:10.1016/j.jpba.2020.113841.

Joshi, S., Kumari, S., and Rathore, A. S. (2021b). Identification and characterization of carbonylation sites in trastuzumab biosimilars. *Int. J. Biol. Macromol.* 169, 95–102. doi:10.1016/j.ijbiomac.2020.12.095.

Joshi, S., Maharana, C., and Rathore, A. S. (2020). An application of Nano Differential Scanning Fluorimetry for Higher Order Structure assessment between mAb originator and biosimilars: Trastuzumab and Rituximab as case studies. *J. Pharm. Biomed. Anal.* 186. doi:10.1016/j.jpba.2020.113270.

Joshi, S., and Rathore, A. S. (2020). Assessment of Structural and Functional Comparability of Biosimilar Products: Trastuzumab as a Case Study. *BioDrugs* 34, 209–223. doi:10.1007/s40259-020-00404-3.

Jung, S. K., Lee, K. H., Jeon, J. W., Lee, J. W., Kwon, B. O., Kim, Y. J., et al. (2014). Physicochemical characterization of Remsima®. *MAbs* 6, 1163–1177. doi:10.4161/mabs.32221.

Kang, J., Halseth, T., Vallejo, D., Najafabadi, Z. I., Sen, K. I., Ford, M., et al. (2020a). Assessment of biosimilarity under native and heat-stressed conditions: rituximab, bevacizumab, and trastuzumab originators and biosimilars. *Anal. Bioanal. Chem.* 412, 763–775. doi:10.1007/s00216-019-02298-9.

Kang, J., Kim, S. Y., Vallejo, D., Hageman, T. S., White, D. R., Benet, A., et al. (2020b). Multifaceted assessment of rituximab biosimilarity: The impact of glycan microheterogeneity on Fc function. *Eur. J. Pharm. Biopharm.* 146, 111–124. doi:10.1016/j.ejpb.2019.12.003.

Kaur, T., Shukla, B. N., Yadav, V. K., Kulkarni, M. J., and Rao, A. (2021). Comparison of glycoprofiles of rituximab versions licensed for sale in India and an analytical approach for quality assessment. *J. Proteomics* 244, 104267. doi:10.1016/J.JPROT.2021.104267.

Kim, J. H., Joshi, S. B., Esfandiary, R., Iyer, V., Bishop, S. M., Volkin, D. B., et al. (2016a). Improved Comparative Signature Diagrams to Evaluate Similarity of Storage Stability Profiles of Different IgG1 mAbs. *J. Pharm. Sci.* 105, 1028–1035. doi:10.1016/j.xphs.2016.01.008.

Kim, J. H., Joshi, S. B., Tolbert, T. J., Middaugh, C. R., Volkin, D. B., and Smalter Hall, A. (2016b). Biosimilarity Assessments of Model IgG1-Fc Glycoforms Using a Machine Learning Approach. *J. Pharm. Sci.* 105, 602–612. doi:10.1016/j.xphs.2015.10.013.

Kim, S., Song, J., Park, S., Ham, S., Paek, K., Kang, M., et al. (2017). Drifts in ADCC-related quality attributes of Herceptin®: Impact on development of a trastuzumab biosimilar. *MAbs* 9, 704–714. doi:10.1080/19420862.2017.1305530.

Kovács, P., Schäfer, T., Háda, V., Hevér, H., Klingelhöfer, S., Nebel, M., et al. (2020). Comparative Physicochemical and Biological Characterisation of the Similar Biological Medicinal Product Teriparatide and Its Reference Medicinal Product. *BioDrugs* 34, 65–75. doi:10.1007/s40259-019-00386-x.

Kumar, M., Pant, A., Bansal, R., Pandey, A., Gomes, J., Khare, K., et al. (2020). Electron microscopy-based semi-automated characterization of aggregation in monoclonal antibody products. *Comput. Struct. Biotechnol. J.* 18, 1458–1465. doi:10.1016/j.csbj.2020.06.009.

Kwon, J.-C., Kwon, O. H., Jeong, R. U., Kim, N., Song, S., Choi, I., et al. (2021). Physicochemical and biological similarity assessment of LBAL, a biosimilar to adalimumab reference product (Humira®). *https://doi.org/10.1080/19768354.2021.1943709* 25, 182–194. doi:10.1080/19768354.2021.1943709.

Largy, E., Cantais, F., Van Vyncht, G., Beck, A., and Delobel, A. (2017). Orthogonal liquid chromatography–mass spectrometry methods for the comprehensive characterization of therapeutic glycoproteins, from released glycans to intact protein level. *J. Chromatogr. A* 1498, 128–146. doi:10.1016/J.CHROMA.2017.02.072.

Lee, C., Jeong, M., Lee, J. A. J., Seo, S., Cho, S. C., Zhang, W., et al. (2017). Glycosylation profile and biological activity of Remicade® compared with Flixabi® and Remsima®. *MAbs* 9, 968–977. doi:10.1080/19420862.2017.1337620.

Lee, J. H., Paek, K., Moon, J. H., Ham, S., Song, J., and Kim, S. (2019a). Biological Characterization of SB3, a Trastuzumab Biosimilar, and the Influence of Changes in Reference Product Characteristics on the Similarity Assessment. *BioDrugs* 33, 411–422. doi:10.1007/s40259-019-00362-5.

Lee, J. H., Yeo, J., Park, H. S., Sung, G., Lee, S. H., Yang, S. H., et al. (2013). Biochemical characterization of a new recombinant TNF receptor-hyFc fusion protein expressed in CHO cells. *Protein Expr. Purif.* 87, 17–26. doi:10.1016/j.pep.2012.09.001.

Lee, J., Kang, H. A., Bae, J. S., Kim, K. D., Lee, K. H., Lim, K. J., et al. (2018a). Evaluation of analytical similarity between trastuzumab biosimilar CT-P6 and reference product using statistical analyses. *MAbs* 10, 547–571. doi:10.1080/19420862.2018.1440170.

Lee, K. H., Lee, J., Bae, J. S., Kim, Y. J., Kang, H. A., Kim, S. H., et al. (2018b). Analytical similarity assessment of rituximab biosimilar CT-P10 to reference medicinal product. *MAbs* 10, 380–396. doi:10.1080/19420862.2018.1433976.

Lee, N., Lee, J. A. J., Yang, H., Baek, S., Kim, S., Kim, S., et al. (2019b). Evaluation of similar quality attribute characteristics in SB5 and reference product of adalimumab. *MAbs* 11, 129–144. doi:10.1080/19420862.2018.1530920.

Lerch, T. F., Sharpe, P., Mayclin, S. J., Edwards, T. E., Polleck, S., Rouse, J. C., et al. (2020). Crystal Structures of PF-06438179/GP1111, an Infliximab Biosimilar. *BioDrugs* 34, 77–87. doi:10.1007/s40259-019-00390-1.

Levy, M. J., Gucinski, A. C., Sommers, C. D., Ghasriani, H., Wang, B., Keire, D. A., et al. (2014). Analytical techniques and bioactivity assays to compare the structure and function of filgrastim (granulocyte-colony stimulating factor) therapeutics from different manufacturers Analysis of Biological Therapeutic Agents and Biosimilars. *Anal. Bioanal. Chem.* 406, 6559–6567. doi:10.1007/s00216-013-7469-x.

Li, C., Rossomando, A., Wu, S. L., and Karger, B. L. (2013). Comparability analysis of anti-CD20 commercial (rituximab) and RNAi-mediated fucosylated antibodies by two LC-MS approaches. *MAbs* 5, 565–575. doi:10.4161/mabs.24814.

Liu, J., Eris, T., Li, C., Cao, S., and Kuhns, S. (2016a). Assessing Analytical Similarity of Proposed Amgen Biosimilar ABP 501 to Adalimumab. *BioDrugs* 30, 321–338. doi:10.1007/s40259-016-0184-3.

Liu, T., Guo, H., Zhu, L., Zheng, Y., Xu, J., Guo, Q., et al. (2016b). Fast Characterization of Fc-Containing Proteins by Middle-Down Mass Spectrometry Following IdeS Digestion. *Chromatographia* 79, 1491–1505. doi:10.1007/s10337-016-3173-2.

López-Morales, C. A., Miranda-Hernández, M. P., Juárez-Bayardo, L. C., Ramírez-Ibáñez, N. D., Romero-Díaz, A. J., Piña-Lara, N., et al. (2015). Physicochemical and biological characterization of a biosimilar trastuzumab. *Biomed Res. Int.* 2015. doi:10.1155/2015/427235.

Lowenthal, M. S., Kilpatrick, E. L., and Phinney, K. W. (2015). Separation of monosaccharides hydrolyzed from glycoproteins without the need for derivatization. *Anal. Bioanal. Chem. 2015 40718* 407, 5453–5462. doi:10.1007/S00216-015-8717-Z.

Magalhaes, V., Mantovani, M., Caruso, C., Facchini, F., Pascon, R., and Cagnacci, P. (2016). Physicochemical and biological comparison of the first Brazilian biosimilar filgrastim with its reference product. *Biosimilars* Volume 6, 45–60. doi:10.2147/bs.s107898.

Magnenat, L., Palmese, A., Fremaux, C., D’Amici, F., Terlizzese, M., Rossi, M., et al. (2017). Demonstration of physicochemical and functional similarity between the proposed biosimilar adalimumab MSB11022 and Humira®. *MAbs* 9, 127–139. doi:10.1080/19420862.2016.1259046.

Maity, S., Ullanat, R., Lahiri, S., Shekar, S., Sodhan, G., Vyas, A., et al. (2011). A non-innovator version of etanercept for treatment of arthritis. *Biologicals* 39, 384–395. doi:10.1016/j.biologicals.2011.08.014.

Mastrangeli, R., Satwekar, A., Cutillo, F., Ciampolillo, C., Palinsky, W., and Longobardi, S. (2017). In-vivo biological activity and glycosylation analysis of a biosimilar recombinant human follicle-stimulating hormone product (Bemfola) compared with its reference medicinal product (GONAL-f). *PLoS One* 12. doi:10.1371/journal.pone.0184139.

McClellan, J. E., Conlon, H. D., Bolt, M. W., Kalfayan, V., Palaparthy, R., Rehman, M. I., et al. (2019). The ‘totality-of-the-evidence’ approach in the development of PF-06438179/GP1111, an infliximab biosimilar, and in support of its use in all indications of the reference product. *Therap. Adv. Gastroenterol.* 12. doi:10.1177/1756284819852535.

Meager, A., Dolman, C., Dilger, P., Bird, C., Giovannoni, G., Schellekens, H., et al. (2011). An assessment of biological potency and molecular characteristics of different innovator and noninnovator interferon-beta products. *J. Interf. Cytokine Res.* 31, 383–392. doi:10.1089/jir.2010.0113.

Mendoza-Macedo, K., Romero-Díaz, A. J., Miranda-Hernández, M. P., Campos-García, V. R., Ramírez-Ibañez, N. D., Juárez-Bayardo, L. C., et al. (2016). Characterization and comparability of biosimilars: A filgrastim case of study and regulatory perspectives for Latin America. *Electron. J. Biotechnol.* 24, 63–69. doi:10.1016/j.ejbt.2016.10.003.

Miao, S., Fan, L., Zhao, L., Ding, D., Liu, X., Wang, H., et al. (2017). Physicochemical and Biological Characterization of the Proposed Biosimilar Tocilizumab. *Biomed Res. Int.* 2017. doi:10.1155/2017/4926168.

Millán-Martín, S., Carillo, S., Füssl, F., Sutton, J., Gazis, P., Cook, K., et al. (2021). Optimisation of the use of sliding window deconvolution for comprehensive characterisation of trastuzumab and adalimumab charge variants by native high resolution mass spectrometry. *Eur. J. Pharm. Biopharm.* 158, 83–95. doi:10.1016/j.ejpb.2020.11.006.

Millán-Martín, S., Jakes, C., Carillo, S., Buchanan, T., Guender, M., Kristensen, D. B., et al. (2020). Inter-laboratory study of an optimised peptide mapping workflow using automated trypsin digestion for monitoring monoclonal antibody product quality attributes. *Anal. Bioanal. Chem.* 412, 6833–6848. doi:10.1007/s00216-020-02809-z.

Miranda-Hernández, M. P., López-Morales, C. A., Perdomo-Abúndez, F. C., Salazar-Flores, R. D., Ramírez-Ibanez, N. D., Pérez, N. O., et al. (2016). New alternatives for autoimmune disease treatments: Physicochemical and clinical comparability of biosimilar etanercept. *J. Immunol. Res.* 2016. doi:10.1155/2016/9697080.

Miranda-Hernández, M. P., López-Morales, C. A., Piña-Lara, N., Perdomo-Abúndez, F. C., Pérez, N. O., Revilla-Beltri, J., et al. (2015). Pharmacokinetic Comparability of a Biosimilar Trastuzumab Anticipated from Its Physicochemical and Biological Characterization. *Biomed Res. Int.* 2015. doi:10.1155/2015/874916.

Miranda-Hernandez, M. P., Lopez-Morales, C. A., Ramirez-Ibanez, N. D., Pinã-Lara, N., Pérez, N. O., Molina-Perez, A., et al. (2015). Assessment of physicochemical properties of rituximab related to its immunomodulatory activity. *J. Immunol. Res.* 2015. doi:10.1155/2015/910763.

Montacir, O., Montacir, H., Eravci, M., Springer, A., Hinderlich, S., Mahboudi, F., et al. (2018a). Bioengineering of rFVIIa biopharmaceutical and structure characterization for biosimilarity assessment. *Bioengineering* 5. doi:10.3390/bioengineering5010007.

Montacir, O., Montacir, H., Eravci, M., Springer, A., Hinderlich, S., Saadati, A., et al. (2017). Comparability study of Rituximab originator and follow-on biopharmaceutical. *J. Pharm. Biomed. Anal.* 140, 239–251. doi:10.1016/j.jpba.2017.03.029.

Montacir, O., Montacir, H., Springer, A., Hinderlich, S., Mahboudi, F., Saadati, A., et al. (2018b). Physicochemical Characterization, Glycosylation Pattern and Biosimilarity Assessment of the Fusion Protein Etanercept. *Protein J.* 37, 164–179. doi:10.1007/s10930-018-9757-y.

Moreno, M. R., Tabitha, T. S., Nirmal, J., Radhakrishnan, K., Yee, C. H., Lim, S., et al. (2016). Study of stability and biophysical characterization of ranibizumab and aflibercept. *Eur. J. Pharm. Biopharm.* 108, 156–167. doi:10.1016/j.ejpb.2016.09.003.

Morimoto, H., Ito, Y., Yoden, E., Horie, M., Tanaka, N., Komurasaki, Y., et al. (2018). Non-clinical evaluation of JR-051 as a biosimilar to agalsidase beta for the treatment of Fabry disease. *Mol. Genet. Metab.* 125, 153–160. doi:10.1016/j.ymgme.2018.07.009.

Narvekar, A., Gawali, S. L., Hassan, P. A., Jain, R., and Dandekar, P. (2020). pH dependent aggregation and conformation changes of rituximab using SAXS and its comparison with the standard regulatory approach of biophysical characterization. *Int. J. Biol. Macromol.* 164, 3084–3097. doi:10.1016/j.ijbiomac.2020.08.148.

Navas, N., Hermosilla, J., Torrente-López, A., Hernández-Jiménez, J., Cabeza, J., Pérez-Robles, R., et al. (2020). Use of subcutaneous tocilizumab to prepare intravenous solutions for COVID-19 emergency shortage: Comparative analytical study of physicochemical quality attributes. *J. Pharm. Anal.* 10, 532–545. doi:10.1016/j.jpha.2020.06.003.

Nupur, N., Chhabra, N., Dash, R., and Rathore, A. S. (2018). Assessment of structural and functional similarity of biosimilar products: Rituximab as a case study. *MAbs* 10, 143–158. doi:10.1080/19420862.2017.1402996.

Nupur, N., Singh, S. K., Narula, G., and Rathore, A. S. (2016). Assessing analytical comparability of biosimilars: GCSF as a case study. *J. Chromatogr. B Anal. Technol. Biomed. Life Sci.* 1032, 165–171. doi:10.1016/j.jchromb.2016.05.027.

Park, S. S., Park, J., Ko, J., Chen, L., Meriage, D., Crouse-Zeineddini, J., et al. (2009). Biochemical assessment of erythropoietin products from Asia versus US epoetin alfa manufactured by Amgen. *J. Pharm. Sci.* 98, 1688–1699. doi:10.1002/jps.21546.

Patil, S. M., Keire, D. A., and Chen, K. (2017). Comparison of NMR and Dynamic Light Scattering for Measuring Diffusion Coefficients of Formulated Insulin: Implications for Particle Size Distribution Measurements in Drug Products. *AAPS J.* 19, 1760–1766. doi:10.1208/s12248-017-0127-z.

Patil, S. M., Nguyen, J., Keire, D. A., and Chen, K. (2020). Sedimentation Velocity Analytical Ultracentrifugation Analysis of Marketed Rituximab Drug Product Size Distribution. *Pharm. Res. 2020 3712* 37, 1–14. doi:10.1007/S11095-020-02961-2.

Peng, J., Patil, S. M., Keire, D. A., and Chen, K. (2018). Chemical Structure and Composition of Major Glycans Covalently Linked to Therapeutic Monoclonal Antibodies by Middle-Down Nuclear Magnetic Resonance. *Anal. Chem.* 90, 11016–11024. doi:10.1021/acs.analchem.8b02637.

Peraza, M. A., Rule, K. E., Shiue, M. H. I., Finch, G. L., Thibault, S., Brown, P. R., et al. (2018). Nonclinical assessments of the potential biosimilar PF-06439535 and bevacizumab. *Regul. Toxicol. Pharmacol.* 95, 236–243. doi:10.1016/j.yrtph.2018.03.020.

Perdomo-Abúndez, F. C., Vallejo-Castillo, L., Vázquez-Leyva, S., López-Morales, C. A., Velasco-Velázquez, M., Pavón, L., et al. (2020). Development and validation of a mass spectrometric method to determine the identity of rituximab based on its microheterogeneity profile. *J. Chromatogr. B Anal. Technol. Biomed. Life Sci.* 1139, 121885. doi:10.1016/j.jchromb.2019.121885.

Pérez-Robles, R., Navas, N., Medina-Rodríguez, S., and Cuadros-Rodríguez, L. (2017). Method for the comparison of complex matrix assisted laser desorption ionization-time of flight mass spectra. Stability of therapeutical monoclonal antibodies. *Chemom. Intell. Lab. Syst.* 170, 58–67. doi:10.1016/j.chemolab.2017.09.008.

Pérez, L. M., Rodríguez Taño, A. de la C., Martín Márquez, L. R., Gómez Pérez, J. A., Garay, A. V., and Santana, R. B. (2019). Conformational characterization of a novel anti-HER2 candidate antibody. *PLoS One* 14. doi:10.1371/journal.pone.0215442.

Planinc, A., Dejaegher, B., Heyden, Y. Vander, Viaene, J., Van Praet, S., Rappez, F., et al. (2017a). LC-MS analysis combined with principal component analysis and soft independent modelling by class analogy for a better detection of changes in N-glycosylation profiles of therapeutic glycoproteins. *Anal. Bioanal. Chem.* 409, 477–485. doi:10.1007/s00216-016-9683-9.

Planinc, A., Dejaegher, B., Vander Heyden, Y., Viaene, J., Van Praet, S., Rappez, F., et al. (2017b). Batch-to-batch N-glycosylation study of infliximab, trastuzumab and bevacizumab, and stability study of bevacizumab. *Eur. J. Hosp. Pharm.*  24, 286–292. doi:10.1136/ejhpharm-2016-001022.

Poppe, L., Jordan, J. B., Rogers, G., and Schnier, P. D. (2015). On the analytical superiority of 1D NMR for fingerprinting the higher order structure of protein therapeutics compared to multidimensional NMR methods. *Anal. Chem.* 87, 5539–5545. doi:10.1021/acs.analchem.5b00950.

Pradhan, G., Sneha, J. M., Sonwane, B. P., Santhakumari, B., Rao, A., and Kulkarni, M. J. (2021). Multiple-parallel-protease digestion coupled with high-resolution mass spectrometry: An approach towards comprehensive peptide mapping of therapeutic mAbs. *J. Proteomics* 232, 104053. doi:10.1016/J.JPROT.2020.104053.

Prakash, A., Mishra, N. N., Vaish, U., Sharma, S., Anand, A., Mahajan, R. V., et al. (2020). Comparative analytical profiling of bevacizumab biosimilars marketed in India: a national control laboratory study. *3 Biotech* 10, 516. doi:10.1007/s13205-020-02506-9.

Quiroz, J., Montes, R., Shi, H., and Roychoudhury, S. (2019). A comparative study of confidence intervals to assess biosimilarity from analytical data. *Pharm. Stat.* 18, 316–328. doi:10.1002/pst.1925.

Rathore, A. S., and Bhambure, R. (2014). Establishing analytical comparability for “biosimilars”: Filgrastim as a case study. *Anal. Bioanal. Chem.* 406, 6569–6576. doi:10.1007/s00216-014-7887-4.

Regl, C., Wohlschlager, T., Holzmann, J., and Huber, C. G. (2017). A Generic HPLC Method for Absolute Quantification of Oxidation in Monoclonal Antibodies and Fc-Fusion Proteins Using UV and MS Detection. *Anal. Chem.* 89, 8391–8398. doi:10.1021/acs.analchem.7b01755.

Rogers, R. S., Nightlinger, N. S., Livingston, B., Campbell, P., Bailey, R., and Balland, A. (2015). Development of a quantitative mass spectrometry multi-attribute method for characterization, quality control testing and disposition of biologics. *MAbs* 7, 881–890. doi:10.1080/19420862.2015.1069454.

Ryan, A. M., Sokolowski, S. A., Ng, C. K., Shirai, N., Collinge, M., Shen, A. C., et al. (2014). Comparative nonclinical assessments of the proposed biosimilar PF-05280586 and rituximab (MabThera®). *Toxicol. Pathol.* 42, 1069–1081. doi:10.1177/0192623313520351.

Sadeghi, N., Kahn, D., Syed, D., Iqbal, O., Abro, S., Eshraghi, R., et al. (2014). Comparative biochemical and functional studies on a branded human recombinant factor viia and a biosimilar equivalent product. *Clin. Appl. Thromb.* 20, 565–572. doi:10.1177/1076029614527496.

Saleem, R. A., Affholter, B. R., Deng, S., Campbell, P. C., Matthies, K., Eakin, C. M., et al. (2015). A chemical and computational approach to comprehensive glycation characterization on antibodies. *MAbs* 7, 1–13. doi:10.1080/19420862.2015.1046663.

Saleem, R., Cantin, G., Wikström, M., Bolton, G., Kuhns, S., McBride, H. J., et al. (2020). Analytical and Functional Similarity Assessment of ABP 710, a Biosimilar to Infliximab Reference Product. *Pharm. Res.* 37, 1–23. doi:10.1007/s11095-020-02816-w.

Sankaran, P. K., Kabadi, P. G., Honnappa, C. G., Subbarao, M., Pai, H. V., Adhikary, L., et al. (2018). Identification and quantification of product-related quality attributes in bio-therapeutic monoclonal antibody via a simple, and robust cation-exchange HPLC method compatible with direct online detection of UV and native ESI-QTOF-MS analysis. *J. Chromatogr. B Anal. Technol. Biomed. Life Sci.* 1102–1103, 83–95. doi:10.1016/j.jchromb.2018.10.019.

Schreiber, S., Yamamoto, K., Muniz, R., and Iwura, T. (2020). Physicochemical analysis and biological characterization of FKB327 as a biosimilar to adalimumab. *Pharmacol. Res. Perspect.* 8. doi:10.1002/prp2.604.

Seo, N., Huang, Z., Kuhns, S., Sweet, H., Cao, S., Wikström, M., et al. (2020). Analytical and functional similarity of biosimilar ABP 798 with rituximab reference product. *Biologicals* 68, 79–91. doi:10.1016/j.biologicals.2020.08.002.

Seo, N., Polozova, A., Zhang, M., Yates, Z., Cao, S., Li, H., et al. (2018). Analytical and functional similarity of Amgen biosimilar ABP 215 to bevacizumab. *MAbs* 10, 678–691. doi:10.1080/19420862.2018.1452580.

Shabestari, A. B., Mojtaba Mostafavi, S., and Malekzadeh, H. (2018). Force Degradation Comparative Study on Biosimilar Adalimumab and Humira. Available at: www.revhipertension.com.

Shaltout, E. L., Al-Ghobashy, M. A., Fathalla, F. A., and Salem, M. Y. (2014). Chromatographic and electrophoretic assessment of Filgrastim biosimilars in pharmaceutical formulations. *J. Pharm. Biomed. Anal.* 97, 72–80. doi:10.1016/j.jpba.2014.04.019.

Shatat, S. M., Eltanany, B. M., Mohamed, A. A., Al-Ghobashy, M. A., Fathalla, F. A., and Abbas, S. S. (2018). Coupling of on-column trypsin digestion–peptide mapping and principal component analysis for stability and biosimilarity assessment of recombinant human growth hormone. *J. Chromatogr. B Anal. Technol. Biomed. Life Sci.* 1072, 105–115. doi:10.1016/j.jchromb.2017.11.007.

Sheen, D. A., Shen, V. K., Brinson, R. G., Arbogast, L. W., Marino, J. P., and Delaglio, F. (2020). Chemometric outlier classification of 2D-NMR spectra to enable higher order structure characterization of protein therapeutics. *Chemom. Intell. Lab. Syst.* 199, 103973. doi:10.1016/J.CHEMOLAB.2020.103973.

Shekhawat, R., Shah, C. K., Patel, A., Srinivasan, S., Kapoor, P., Patel, S., et al. (2019). Structural similarity, characterization of Poly Ethylene Glycol linkage and identification of product related variants in biosimilar pegfilgrastim. *PLoS One* 14. doi:10.1371/journal.pone.0212622.

Shen, Z., Wang, Y., Xu, H., Zhang, Q., Sha, C., Sun, B., et al. (2021). Analytical comparability assessment on glycosylation of ziv-aflibercept and the biosimilar candidate. *Int. J. Biol. Macromol.* 180, 494–509. doi:10.1016/j.ijbiomac.2021.03.020.

Singh, S. K., Kumar, D., Malani, H., and Rathore, A. S. (2021). LC–MS based case-by-case analysis of the impact of acidic and basic charge variants of bevacizumab on stability and biological activity. *Sci. Rep.* 11. doi:10.1038/s41598-020-79541-2.

Singh, S. K., Pokalwar, S., Bose, S., Gupta, S., Almal, S., and Ranbhor, R. S. (2018). Structural and functional comparability study of anti-CD20 monoclonal antibody with reference product. *Biol. Targets Ther.* 12, 159–170. doi:10.2147/BTT.S187744.

Skala, W., Wohlschlager, T., Senn, S., Huber, G. E., and Huber, C. G. (2018). MoFi: A Software Tool for Annotating Glycoprotein Mass Spectra by Integrating Hybrid Data from the Intact Protein and Glycopeptide Level. *Anal. Chem.* 90, 5728–5736. doi:10.1021/acs.analchem.8b00019.

Skrlin, A., Radic, I., Vuletic, M., Schwinke, D., Runac, D., Kusalic, T., et al. (2010). Comparison of the physicochemical properties of a biosimilar filgrastim with those of reference filgrastim. *Biologicals* 38, 557–566. doi:10.1016/j.biologicals.2010.05.002.

Song, Y. E., Dubois, H., Hoffmann, M., D́Eri, S., Fromentin, Y., Wiesner, J., et al. (2021). Automated mass spectrometry multi-attribute method analyses for process development and characterization of mAbs. *J. Chromatogr. B Anal. Technol. Biomed. Life Sci.* 1166, 122540. doi:10.1016/j.jchromb.2021.122540.

Song, Y., Yu, D., Mayani, M., Mussa, N., and Li, Z. J. (2018). Monoclonal antibody higher order structure analysis by high throughput protein conformational array. *https://doi.org/10.1080/19420862.2017.1421880* 10, 397–405. doi:10.1080/19420862.2017.1421880.

Sorensen, M., Harmes, D. C., Stoll, D. R., Staples, G. O., Fekete, S., Guillarme, D., et al. (2016). Comparison of originator and biosimilar therapeutic monoclonal antibodies using comprehensive two-dimensional liquid chromatography coupled with time-of-flight mass spectrometry. *MAbs* 8, 1224–1234. doi:10.1080/19420862.2016.1203497.

Sörgel, F., Lerch, H., and Lauber, T. (2010). Physicochemical and Biologic Comparability of a Biosimilar Granulocyte Colony-Stimulating Factor with Its Reference Product. *Biodrugs*.

Sörgel, F., Schwebig, A., Holzmann, J., Prasch, S., Singh, P., and Kinzig, M. (2015). Comparability of biosimilar filgrastim with originator filgrastim: Protein characterization, pharmacodynamics, and pharmacokinetics. *BioDrugs* 29, 123–131. doi:10.1007/s40259-015-0124-7.

Stoll, D. R., Harmes, D. C., Danforth, J., Wagner, E., Guillarme, D., Fekete, S., et al. (2015). Direct Identification of Rituximab Main Isoforms and Subunit Analysis by Online Selective Comprehensive Two-Dimensional Liquid Chromatography-Mass Spectrometry. *Anal. Chem.* 87, 8307–8315. doi:10.1021/acs.analchem.5b01578.

Suba, D., Urbányi, Z., and Salgó, A. (2015). Capillary isoelectric focusing method development and validation for investigation of recombinant therapeutic monoclonal antibody. *J. Pharm. Biomed. Anal.* 114, 53–61. doi:10.1016/j.jpba.2015.04.037.

Suba, D., Urbányi, Z., and Salgó, A. (2016). Method development and qualification of capillary zone electrophoresis for investigation of therapeutic monoclonal antibody quality. *J. Chromatogr. B Anal. Technol. Biomed. Life Sci.* 1032, 224–229. doi:10.1016/j.jchromb.2016.07.026.

Switzar, L., Nicolardi, S., Rutten, J. W., Oberstein, S. A. J. L., Aartsma-Rus, A., and Van Der Burgt, Y. E. M. (2016). In-Depth Characterization of Protein Disulfide Bonds by Online Liquid Chromatography-Electrochemistry-Mass Spectrometry. *J. Am. Soc. Mass Spectrom.* 27, 50–58. doi:10.1007/s13361-015-1258-z.

Szekrenyes, A., Szigeti, M., Dvorakova, V., Jarvas, G., and Guttman, A. (2020). Quantitative comparison of the N-glycosylation of therapeutic glycoproteins using the Glycosimilarity Index. A tutorial. *TrAC - Trends Anal. Chem.* 122, 115728. doi:10.1016/j.trac.2019.115728.

Tabasinezhad, M., Mahboudi, F., Wenzel, W., Rahimi, H., Walther, T. H., Blattner, C., et al. (2019). The transient production of anti-TNF-α antibody Adalimumab and a comparison of its characterization to the biosimilar Cinorra. *Protein Expr. Purif.* 155, 59–65. doi:10.1016/j.pep.2018.11.006.

Tan, Q., Guo, Q., Fang, C., Wang, C., Li, B., Wang, H., et al. (2012). Characterization and comparison of commercially available TNF receptor 2-Fc fusion protein products. *MAbs* 4, 761–774. doi:10.4161/mabs.22276.

Tani, J., Ito, Y., Tatemichi, S., Yamakami, M., Fukui, T., Hatano, Y., et al. (2020). Physicochemical and biological evaluation of JR-131 as a biosimilar to a long-acting erythropoiesis-stimulating agent darbepoetin alfa. *PLoS One* 15. doi:10.1371/journal.pone.0231830.

Thennati, R., Singh, S. K., Nage, N., Patel, Y., Bose, S. K., Burade, V., et al. (2018). Analytical characterization of recombinant hCG and comparative studies with reference product. *Biol. Targets Ther.* 12, 23–35. doi:10.2147/BTT.S141203.

Trabik, Y. A., Moenes, E. M., Al-Ghobashy, M. A., Nebsen, M., and Ayad, M. F. (2020). Analytical comparability study of anti-CD20 monoclonal antibodies rituximab and obinutuzumab using a stability-indicating orthogonal testing protocol: Effect of structural optimization and glycoengineering. *J. Chromatogr. B Anal. Technol. Biomed. Life Sci.* 1159. doi:10.1016/j.jchromb.2020.122359.

Treuheit, N. A., Crawford, N. F., Maki, S., Payne, J., and Allen, J. (2020). Receptor-binding hydrogen–deuterium exchange mass spectrometry as an additional measurement of biosimilarity. *J. Pharm. Investig.* 50, 413–423. doi:10.1007/s40005-019-00465-9.

Tsuda, M., Otani, Y., Yonezawa, A., Masui, S., Ikemi, Y., Denda, M., et al. (2018). Analysis of Glycoforms and Amino Acids in Infliximab and a Biosimilar Product Using New Method with LC/TOF-MS. *Biol. Pharm. Bull.* 41, 1716–1721. doi:10.1248/bpb.b18-00491.

Upton, R., Bell, L., Guy, C., Caldwell, P., Estdale, S., Barran, P. E., et al. (2016). Orthogonal Assessment of Biotherapeutic Glycosylation: A Case Study Correlating N-Glycan Core Afucosylation of Herceptin with Mechanism of Action. *Anal. Chem.* 88, 10259–10265. doi:10.1021/acs.analchem.6b02994.

Vanhoenacker, G., Vandenheede, I., David, F., Sandra, P., and Sandra, K. (2015). Comprehensive two-dimensional liquid chromatography of therapeutic monoclonal antibody digests. *Anal. Bioanal. Chem.* 407, 355–366. doi:10.1007/s00216-014-8299-1.

Velasco-Velázquez, M. A., Salinas-Jazmín, N., Hisaki-Itaya, E., Cobos-Puc, L., Xolalpa, W., González, G., et al. (2017). Extensive preclinical evaluation of an infliximab biosimilar candidate. *Eur. J. Pharm. Sci.* 102, 35–45. doi:10.1016/j.ejps.2017.01.038.

Viski, K., Gengeliczki, Z., Lenkey, K., and Baranyáné Ganzler, K. (2016). Parallel development of chromatographic and mass-spectrometric methods for quantitative analysis of glycation on an IgG1 monoclonal antibody. *J. Chromatogr. B Anal. Technol. Biomed. Life Sci.* 1032, 198–204. doi:10.1016/j.jchromb.2016.04.043.

Visser, J., Feuerstein, I., Stangler, T., Schmiederer, T., Fritsch, C., and Schiestl, M. (2013). Physicochemical and functional comparability between the proposed biosimilar rituximab GP2013 and originator rituximab. *BioDrugs* 27, 495–507. doi:10.1007/s40259-013-0036-3.

Wagner, E., Colas, O., Chenu, S., Goyon, A., Murisier, A., Cianferani, S., et al. (2020). Determination of size variants by CE-SDS for approved therapeutic antibodies: Key implications of subclasses and light chain specificities. *J. Pharm. Biomed. Anal.* 184, 113166. doi:10.1016/j.jpba.2020.113166.

Wang, D., Park, J., Patil, S. M., Smith, C. J., Leazer, J. L., Keire, D. A., et al. (2020). An NMR-Based Similarity Metric for Higher Order Structure Quality Assessment Among U.S. Marketed Insulin Therapeutics. *J. Pharm. Sci.* 109, 1519–1528. doi:10.1016/j.xphs.2020.01.002.

Wang, H., Wu, L., Wang, C., Xu, J., Yin, H., Guo, H., et al. (2021). Biosimilar or Not: Physicochemical and Biological Characterization of MabThera and Its Two Biosimilar Candidates. *ACS Pharmacol. Transl. Sci.* 2021, 801. doi:10.1021/acsptsci.0c00225.

Wang, T., and Chow, S. C. (2017). On the establishment of equivalence acceptance criterion in analytical similarity assessment. *J. Biopharm. Stat.* 27, 206–212. doi:10.1080/10543406.2016.1265539.

Wang, Y. A., Wu, D., Auclair, J. R., Salisbury, J. P., Sarin, R., Tang, Y., et al. (2017). Integrated Bottom-Up and Top-Down Liquid Chromatography-Mass Spectrometry for Characterization of Recombinant Human Growth Hormone Degradation Products. *Anal. Chem.* 89, 12771–12777. doi:10.1021/acs.analchem.7b03026.

Wen, J., Lord, H., Knutson, N., and Wikström, M. (2020). Nano differential scanning fluorimetry for comparability studies of therapeutic proteins. *Anal. Biochem.* 593, 113581. doi:10.1016/j.ab.2020.113581.

Wildner, S., Huber, S., Regl, C., Huber, C. G., Lohrig, U., and Gadermaier, G. (2019). Aptamers as quality control tool for production, storage and biosimilarity of the anti-CD20 biopharmaceutical rituximab. *Sci. Reports 2019 91* 9, 1–14. doi:10.1038/s41598-018-37624-1.

Winstel, R., Wieland, J., Gertz, B., Mueller, A., and Allgaier, H. (2017). Manufacturing of Recombinant Human Follicle-Stimulating Hormone Ovaleap® (XM17), Comparability with Gonal-f®, and Performance/Consistency. *Drugs R D* 17, 305–312. doi:10.1007/s40268-017-0182-z.

Xie, H., Chakraborty, A., Ahn, J., Yu, Y. Q., Dakshinamoorthy, D. P., Gilar, M., et al. (2010). Rapid comparison of a candidate biosimilar to an innovator monoclonal antibody with advanced liquid chromatography and mass spectrometry technologies. *MAbs* 2, 379–394. doi:10.4161/mabs.11986.

Xie, L., Zhang, E., Xu, Y., Gao, W., Wang, L., Xie, M. H., et al. (2020). Demonstrating Analytical Similarity of Trastuzumab Biosimilar HLX02 to Herceptin® with a Panel of Sensitive and Orthogonal Methods Including a Novel FcγRIIIa Affinity Chromatography Technology. *BioDrugs* 34, 363–379. doi:10.1007/s40259-020-00407-0.

Xu, J., Shao, Z., Han, X., Huang, Y., Zou, X., and Shen, Y. (2021). Similarity assessment by multivariate statistics method based on the distance between biosimilar and originator. *Bioresour. Bioprocess.* 8, 24. doi:10.1186/s40643-021-00378-2.

Xu, Y., Xie, L., Zhang, E., Gao, W., Wang, L., Cao, Y., et al. (2019). Physicochemical and functional assessments demonstrating analytical similarity between rituximab biosimilar HLX01 and the MabThera®. *MAbs* 11, 606–620. doi:10.1080/19420862.2019.1578147.

Yang, Y., Liu, F., Franc, V., Halim, L. A., Schellekens, H., and Heck, A. J. R. (2016). Hybrid mass spectrometry approaches in glycoprotein analysis and their usage in scoring biosimilarity. *Nat. Commun.* 7, 1–10. doi:10.1038/ncomms13397.

Yi, X., Liu, W., and Yu, F. (2019). A simple MS method to characterize the higher order structures of antibody therapeutics. *Eur. J. Pharm. Sci.* 131, 111–118. doi:10.1016/j.ejps.2019.01.038.

Yu, C., Zhang, F., Xu, G., Wu, G., Wang, W., Liu, C., et al. (2020). Analytical Similarity of a Proposed Biosimilar BVZ-BC to Bevacizumab. *Anal. Chem.* 92, 3161–3170. doi:10.1021/acs.analchem.9b04871.

Zhang, E., Xie, L., Qin, P., Lu, L., Xu, Y., Gao, W., et al. (2020). Quality by Design–Based Assessment for Analytical Similarity of Adalimumab Biosimilar HLX03 to Humira®. *AAPS J.* 22. doi:10.1208/s12248-020-00454-z.

Zhang, Y., and Qi, P. (2021). Determination of Free Sulfhydryl Contents for Proteins Including Monoclonal Antibodies by Use of SoloVPE. *J. Pharm. Biomed. Anal.*, 114092. doi:10.1016/j.jpba.2021.114092.

Zhao, Y. Y., Wang, N., Liu, W. H., Tao, W. J., Liu, L. L., and Shen, Z. D. (2016). Charge variants of an avastin biosimilar isolation, characterization, in vitro properties and pharmacokinetics in rat. *PLoS One* 11. doi:10.1371/journal.pone.0151874.

Zheng, J., Yin, D., Yuan, M., and Chow, S. C. (2019). Simultaneous confidence interval methods for analytical similarity assessment. *J. Biopharm. Stat.* 29, 920–940. doi:10.1080/10543406.2019.1657142.
